# Supplementary material for: A Xanthomonas transcription activator-like effector is trapped in nonhost plants for immunity
Source: Plant Commun. 2021 Oct 14;3(1):100249. doi: 10.1016/j.xplc.2021.100249 (PMC8760140; doi:10.1016/j.xplc.2021.100249)
Supplement: Document S1. Supplemental methods, Supplemental Figures 1–7, and Supplemental Tables 1–3 [file mmc1.pdf]

Plant Communications, Volume *3*

Supplemental information

A *Xanthomonas* transcription activator-like effector is trapped in non-host plants for immunity

Fazal Haq, Xiameng Xu, Wenxiu Ma, Syed Mashab Ali Shah, Linlin Liu, Bo Zhu, Lifang Zou, and Gongyou Chen

Supplemental Information

The following Supplemental Information is available for this article:

Supplemental Methods

Methods S1. DNA manipulation and plasmid construction.

DNA gel extraction and plasmid miniprep kits were purchased from Axygen (Beijing, China). DNA polymerases, restriction endonucleases, and molecular weight markers were obtained from TaKaRa (Dalian, China). DNA ligase was purchased from Thermo Fisher Scientific (USA), and Ni-NTA purification resin was provided by Shanghai Yisheng Biotechnology Co., Ltd. Reverse transcription PCR and fluorescent quantitative PCR kits were purchased from TransGen Biotech (Beijing) Co., Ltd. Primers were designed with Primer Premier 5 Design Program (Premier Biosoft International, Palo Alto, CA, USA) and were synthesized by Generay (Shanghai, China). The constructs were confirmed by Sanger sequencing, which was a service provided by Biosune (Shanghai, China).

Cloning *Xoo* TALEs and *avrXa10* derivatives. For transient expression assays in *N. benthamiana*, *Xoo* TALEs were cloned in binary vector pHB (Mao et al., 2005), which contains the 35S promoter and a flag epitope upstream of the polylinker. The N- and C-terminal ends of *avrXa10* were amplified from plasmid pZW-avrXa10 (Zhu et al., 1998) using the primer sets avrXa10-F/avrXa10-N-R and avrXa10-C-F/avrXa10-R with *Hin*dIII/*Sph*I and *Sph*I/*Xba*I sites (Table S3), respectively. The amplified fragments were purified, sequenced and digested with *Hin*dIII/*Sph*I (N-terminus) and *Sph*I/*Xba*I (C-terminus). The two digested fragments were ligated together and cloned into pHB digested with *Hin*dIII and *Xba*I. The resulting plasmid was named pHB-AvrXa10ΔCRR and contained the N and C-terminal regions of AvrXa10, but lacked the *Sph*I fragment containing the central repeat region (CRR). The central repeat region (CRR) of AvrXa10 was digested from plasmid pZW-avrXa10 with *Sph*I and cloned into *Sph*I-digested pHB-avrXa10ΔCRR plasmid, resulting in pHB-AvrXa10 (pHB with full-length *avrXa10*). Clones containing the 17 *Xoo tal* genes in pBluescript (unpublished data) were digested with *Bam*HI to release the CRR regions and portions of the N and C-terminal ends (N+CRR+C fragments). The pHB-AvrXa10 construct was digested with *Bam*HI to release the CRR and flanking regions, and the N+CRR+C fragments from the 17 *tal* genes were individually inserted into the *Bam*HI site, giving rise to 17 *tal* genes in pHB (Table S2).

To construct the AD mutant of *avrXa10*, pHB-avrXa10 was digested with *Sal*I (unique site located after the C-terminal *Sph*I site in *avrXa10*) and *Xba*I to release the AD domain along with two nuclear localization signal (NLS) motifs. The NLS motifs (~176 bp upstream of the AD domain) were amplified from pHB-avrXa10 using primers avrXa10-C(ΔAD)-F/avrXa10-C(ΔAD)-R; a stop codon was included in the latter primer (Table S3). The PCR product was sequenced, digested with *Sal*I and *Xba*I, and cloned into *Sal*I/*Xba*I-digested pHB-avrXa10, resulting in pHB-avrXa10ΔAD.

Methods S2. Transient expression assays in tobacco and rice.

The binary vector pHB was used to clone and express TALEs in *N. benthamiana* (Mao et al., 2005). pHB contains the CaMV 35S promoter and a flag-tag upstream of the polylinker, and *tal* genes were cloned into the vector polylinker. pHB constructs were transformed into *Agrobacterium* strain EHA105 by freeze-thaw method; and *Agrobacterium* transformants were cultured in LB medium to OD_{600}=1.5. Cultures were harvested by centrifugation, washed and resuspended in infiltration buffer (10 mM $MgCl_2$, 0.2 mM acetosyringone and 200 mM MES, pH 5.6) to a final concentration of OD_{600}=1.0. Buffer-supplemented *Agrobacterium* strains were incubated at room temperature for 1 h and then infiltrated (OD_{600} = 1.0) into *N. benthamiana* leaves with needleless syringes for transient expression assays.

Transient expression assays in rice. For transient expression in rice protoplasts, pRTVcHA was used, which contains the maize ubiquitin (Ubi) 1 promoter and a C-terminal HA-tag epitope (He et al., 2018). The full-length coding sequence (CDS) of *Nb20731g* was amplified from tobacco cDNA using primer pairs 20731g-F/20731g-R (Table S3). The full-length CDS of *LOC_Os03g41110*, *LOC_Os09g26210*, *LOC_Os08g44190*, *LOC_Os03g13600* was amplified from rice (Nipponbare) cDNA using primer pairs Os41110-F/Os41110-R, Os26210-F/Os26210-R, Os44190-F/Os44190-R, Os13600-F/Os13600-R, respectively (Table S3). The PCR product was purified, sequenced and cloned into the *Bam*HI/*Not*I sites of pRTVcHA, giving rise to pRTVcHA-20731g. The full-length CDS of rice gene *Xa10* was amplified from pB-Xa10 (Table S2) using primer pairs Xa10-F/Xa10-R (Table S3). The PCR product was purified, sequenced and cloned into the *Bam*HI/*Not*I sites of pRTVcHA, giving rise to pRTVcHA-Xa10.

Methods S3. Western blot assays.

In planta expression of the TALE and dTALE proteins cloned in pHB vector was confirmed by western blotting using the flag-tag epitope. Briefly, infiltrated leaf samples were collected at 48 hpi and macerated in protein lysis buffer (50 mM Tris-MES, 0.5 M sucrose, 1 mM $MgCl_2$, 10 mM EDTA, 5 mM DTT and protease inhibitor cocktail, pH 8.0). The protein samples were separated on 8% SDS-PAGE and transferred to PVDF membranes for immunoblotting using anti-FLAG serum (Transgene, Beijing, China) as described previously (Haq et al., 2020).

Methods S4. AvrXa10 delivery into plant cells via *Xanthomonas*

The Plasmid pHZW-AvrXa10 (Table S2), which contains a FLAG-tag epitope in the *c*-terminus of AvrXa10, was electroporated (2.5 kv, 4 ms) into *Xanthomonas axonopodis* pv. *glycines* (*Xag*) strain ATCC43911. The expression of AvrXa10 was confirmed by western blot using mouse anti-flag antibody as described previously (Haq et al., 2020). The strain ATCC43911 containing AvrXa10 or empty vector pHM1 were cultured overnight in NA liquid medium. The bacterial cells were collected via centrifugation, washed twice with 10mM $MgCl_2$ and, resuspended in 10mM $MgCl_2$ to OD_{600} = 0.2. The suspensions were infiltrated into *N. benthamiana* leaves with needleless syringe. Infiltration of simply 10mM buffer served as a mock. The leaf phenotype was photographed at 24hpi. This experiments were repeated three times with similar results.

Methods S5. RNA isolation, RT-PCR and qRT-PCR.

Total RNA was extracted from inoculated leaf tissue using RNAiso plus reagent (Takara, China). The quality of RNA was checked with the NanoDrop spectrophotometer (Eppendorf) and then reverse transcribed using EasyScript® One Step gDNA Removal and cDNA Synthesis Supermix (TransGen). Semi-quantitative expression was carried out by RT-PCR using 2x Taq PCR StarMix (TaKaRa). PCR amplification conditions were as follows: 98˚C for 15 s, 60˚C for 30 s, and 72˚C for 12 s; amplification continued for a total of 28 cycles. Real-time quantitative PCR (RT-qPCR) was performed using TransStart® Tip Green qPCR SuperMix (TransGen) and the ABI 7500 quantitative PCR system. *NbEF1α* was used as an internal control. Primers used for qRT-PCR are listed in Table S3.

Methods S6. Protein expression and purification.

The construct pET30a-pthXo1 was used to construct pET30a-avrXa10. In a previous report (Ma et al., 2018) a multi-step process was used to clone *pthXo1* in pET30a. For cloning avrXa10 in pET30a, the central portion of *pthXo1* in pET30a-pthXo1 was replaced with avrXa10 at conserved *Not*I and *Sal*I sites to give rise pET30a-avrXa10 (His-AvrXa10). The *Sal*I site is conserved in TALE genes containing the C-terminal flag sequence. The generated construct was then introduced into *E. coli* BL21 (DE3). The bacteria were grown in LB medium containing 25 μg/ml kanamycin at 37℃ to an OD_{600} of 0.5. The expression of His-AvrXa10 was induced by adding 0.5mM IPTG (isopropyl-b-D-thiogalactopyranoside) and incubating at 16℃ for 14 h. Bacterial cells were incubated on ice for 20 minutes to stop growth and then harvested by centrifugation at 5000 rpm for 10 min. The cells were washed one time in PBS and resuspended in 10 mM PBS (pH 7.5) supplemented with cocktail and phenylmethylsulfonyl fluoride (PMSF). The cells were sonicated for 10-20 min and then centrifuged at 8000 rpm for 20 min at 4℃. Proteins were purified from the supernatant using Ni-NTA His Resin (Shanghai Yisheng Biotechnology Co., Ltd.) according to the manufacturer's instructions.

Methods S7. Subcellular localization of NbZnFP1.

The subcellular localization of NbZnFP1 was investigated. NbZnFP1 was amplified using primers NbZnFP1-YFP-F/ NbZnFP1-YFP-R and cloned in the YFP vector with *Kpn*I and *Sma*I sites, resulting in construct NbZnFP1-YFP. *Agrobacterium* strain GV3101 containing the construct YFP-NbZnFP1 or YFP (empty vector) were transiently expressed in *Nb* leaves. At 2 dpi, Nb leaves were visualized with a Leica confocal microscope; the excitation wavelength for YFP was 488 nm, and 520–550 nm was used for emission.

110 **Supplemental Figures**

111

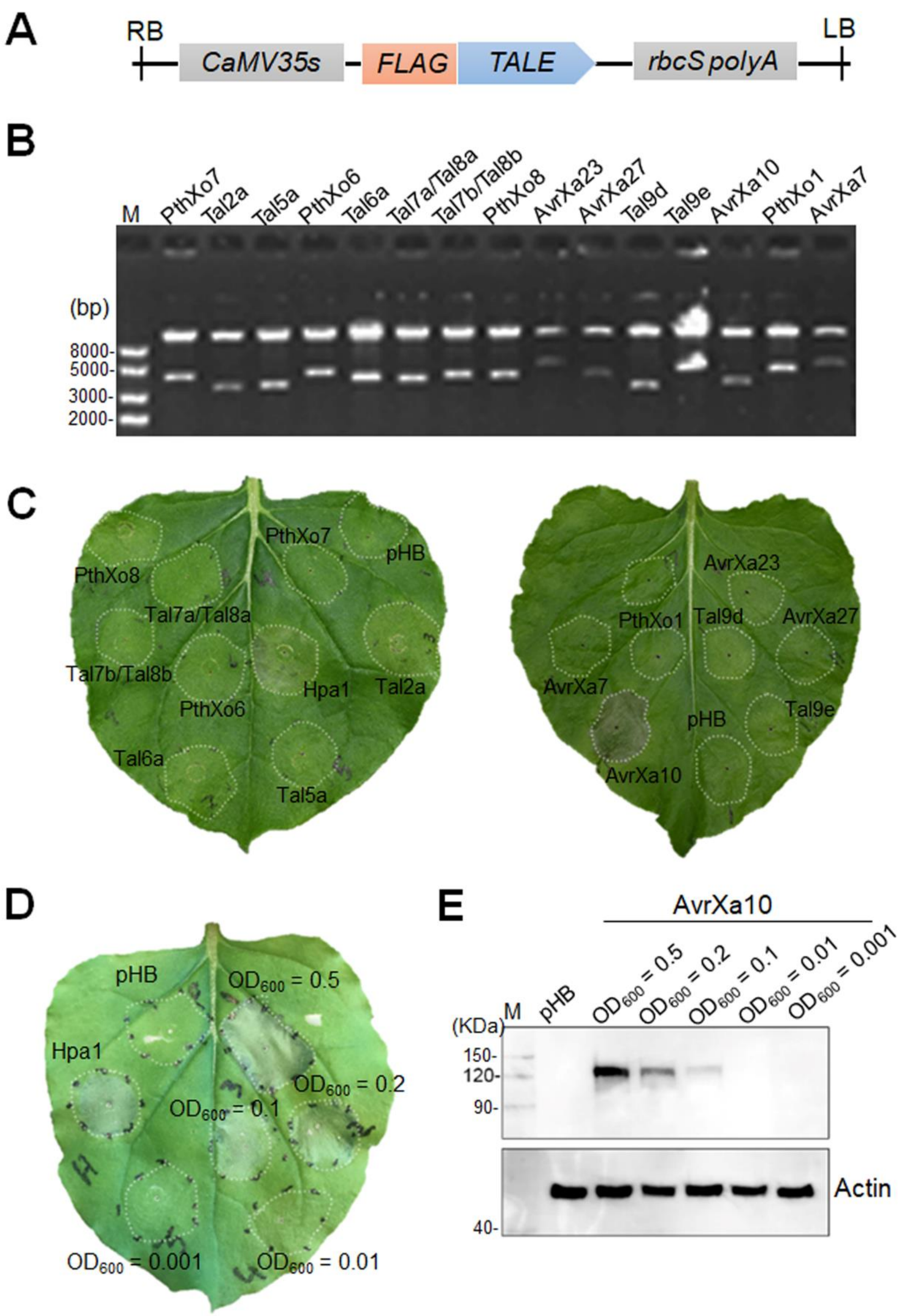

112

113 **Figure S1. Analysis of HR-like cell death in *N. benthamiana*. (A)** Schematic diagram showing
114 approach for cloning *TALEs* in the binary vector pHB. Abbreviations: CaMV 35S, cauliflower mosaic
115 35S promoter; FLAG, N-terminal flag epitope tag driven by 35S promoter; TALE, insertion site for *Xoo*

TALE gene; rbcS, ribulose-1,5-bisphosphate carboxylase, small subunit; polyA, polyadenylation site. **(B)** Confirmation of *tal*-gene insert in pHB vector by restriction digestion with *BamH*I. **(C)** Phenotype of *Nb* leaves expressing TALE proteins; the empty vector pHB was transformed as a negative control. *Agrobacterium* strains containing different TALE constructs or empty pHB vector were infiltrated into *Nb* leaves at OD_{600}=1.0. Leaves were photographed at three days post-infiltration (dpi). **(D)** *Agrobacterium* carrying AvrXa10 was infiltrated into *Nb* leaves at OD_{600}=0.5, 0.2, 0.1, 0.01 and 0.001. *Agrobacterium* strains carrying Hpa1 and empty pHB served as positive and negative controls, respectively. *Nb* leaves were photographed at 4 dpi. **(E)** Immunodetection of flag-tagged AvrXa10 in *N. benthamiana* at different OD values at 3 dpi. pHB was a negative control, and Actin was used as a loading control.

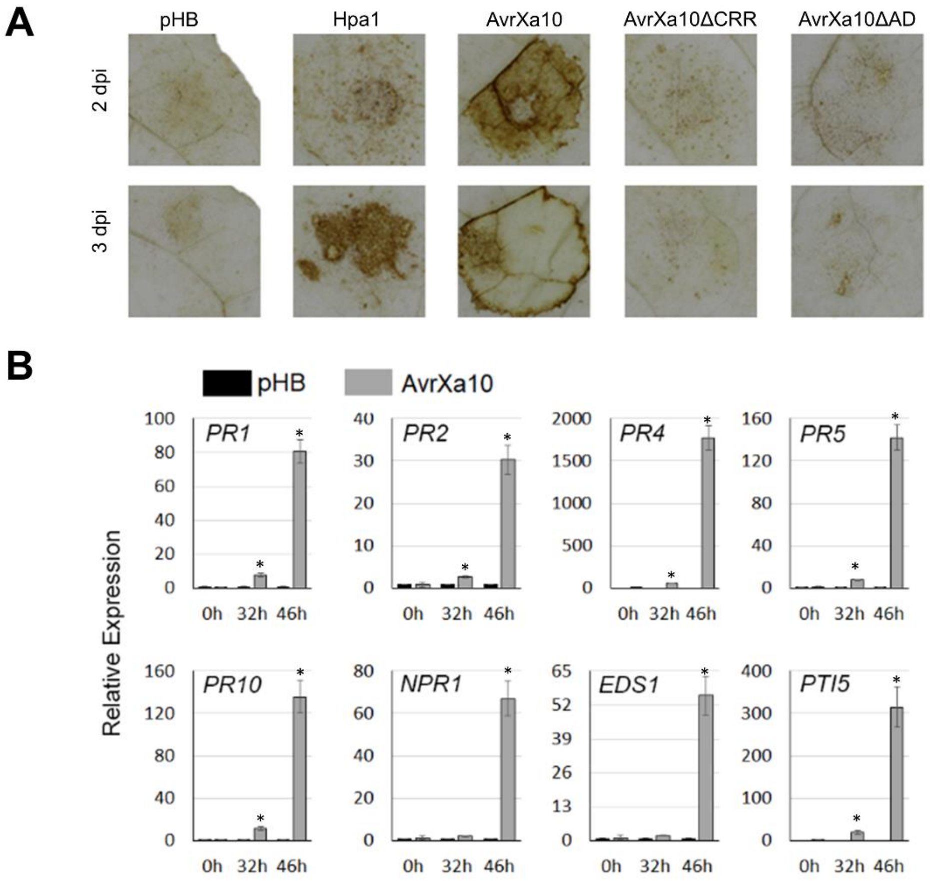

Figure S2. Detection of ROS and expression of defense-related genes in *N. benthamiana* leaves agro-infiltrated with pHB-AvrXa10 and derivatives. (A) ROS detection in *Nb* leaves infiltrated with *Agrobacterium* containing pHB-AvrXa10, pHB-HpaI (positive control) pHB-AvrXa10ΔCRR, pHB-AvrXa10ΔAD, and pHB at 2 and 3 dpi. Agro-infiltrated leaves of *Nb* were collected and incubated in DAB (1 mg/ml), 0.05% Tween-20 and 10 mM sodium phosphate buffer (pH 7.0) in darkness for 6-8 h. Chlorophyll was removed as described (Ma *et al.*, 2020), and leaves were stored in 65% ethanol and photographed. HpaI and empty pHB were used as positive and negative controls, respectively. **(B)** RT-qPCR expression analysis of defense-related genes, *PR1, PR2*, *PR4*, *PR5, PR10*, *EDS1*, *NPR1*, and *PTI5* in *N. benthamiana* leaves expressing AvrXa10 or pHB (control). Expression was monitored at 0, 32, and 46 hpi. Error bars represent means and means ± SD (n = 3), and columns labeled with asterisks show significant differences (P≤0.01). The results shown are representative of three independent replicates.

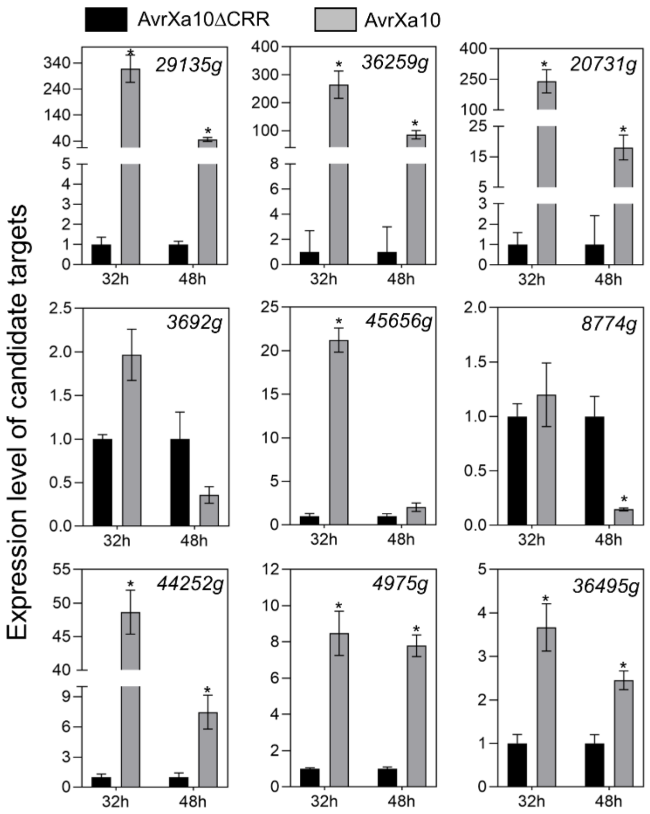

Figure S3. RT-qPCR analysis of putative AvrXa10 target genes in *N. benthamiana*. Leaves of four-week-old *Nb* were infiltrated with *Agrobacterium* strains carrying AvrXa10 or AvrXa10ΔCRR and collected at 32 and 48 hpi for RNA isolation. *NbEF1a* was used as an internal control. Error bars represent means ± SD (n =3), and columns labeled with asterisks show significant differences (P<0.05) in the AvrXa10-expressing leaves as compared to the AvrXa10ΔCRR control. The results shown are representative of three independent replicates.

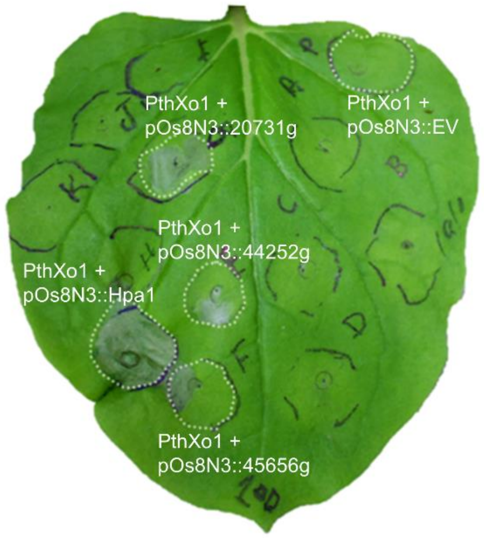

Figure S4. *in-vivo* reporter assay for HR in *N. benthamiana*. *Agrobacterium* strains containing the effector construct (pHB-pthXo1) and one of the six reporter constructs were infiltrated into five-week-old *Nb* leaves, which were evaluated for the HR at 4-7 dpi. Co-infiltration of pHB-PthXo1 with

pOs8N3-EV or pOs8N3-Hpa1 served as a negative and positive control, respectively. Representative results were chosen from five independent experiments.

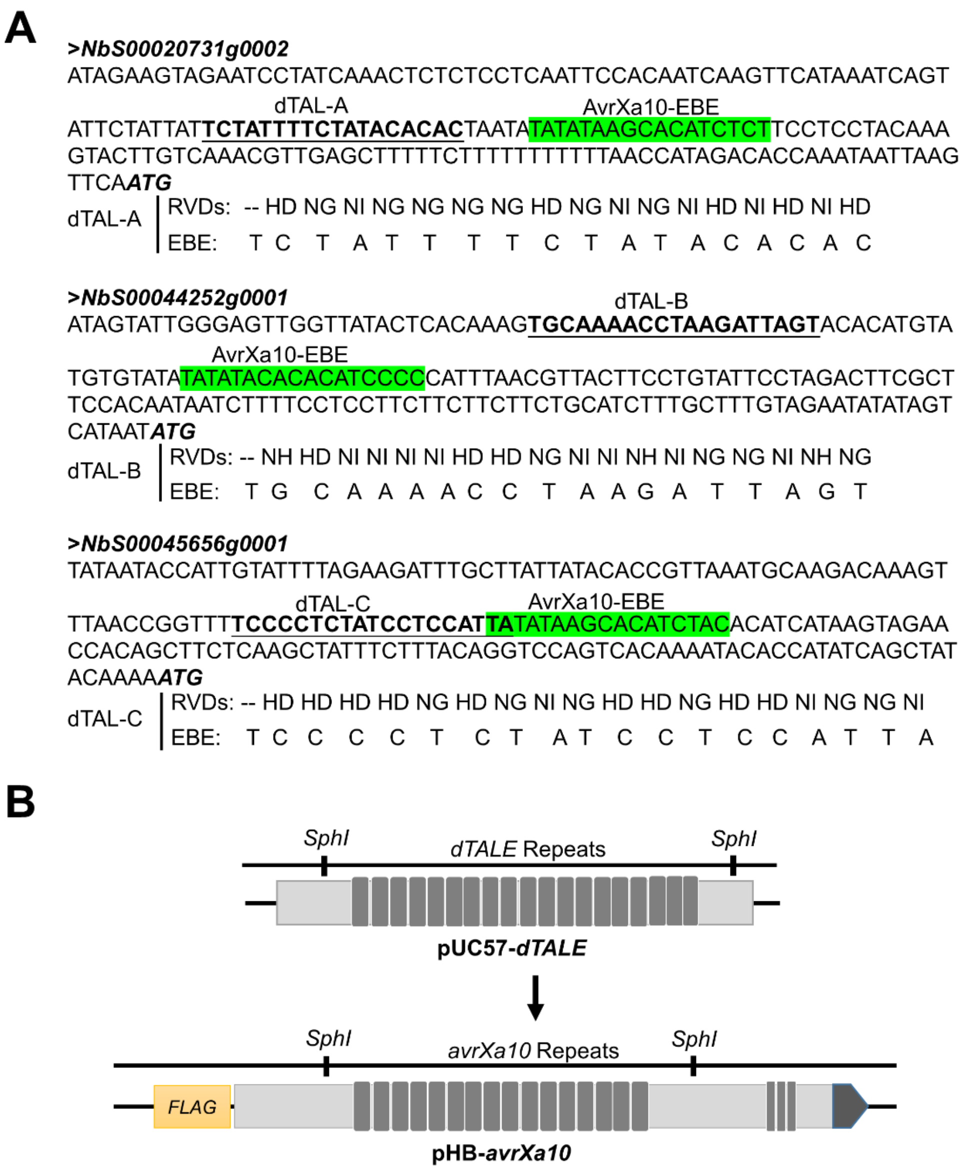

Figure S5. Construction of artificial dTALEs to activate the expression of *Nb20731g*, *Nb44252g* and *Nb45656g*. (A) Promoter sequences of *Nb20731g*, *Nb44252g* and *Nb45656g*. The bold underscored regions indicate sites for dTALE insertion. dTAL-A was designed to target an18-bp region upstream of AvrXa10-EBE (green-shaded nucleotides) in the promoter of *Nb20731g*. The dTAL-B and dTAL-C were designed to target 19-bp regions upstream of AvrXa10-EBE (green-shaded nucleotides) in the promoters of *Nb44252g* and *Nb45656g*, respectively. The repeat variable diresidues (RVDs) of each dTALE and their target EBE are shown. **(B)** Schematic map showing the cloning of dTALEs into pHB vector. The *SphI* fragment of each dTALE was replaced with the *Sph*I fragment of AvrXa10 in the pHB-AvrXa10 constructs to produce pHB-dTAL-A, pHB-dTAL-B, and pHB-dTAL-C.

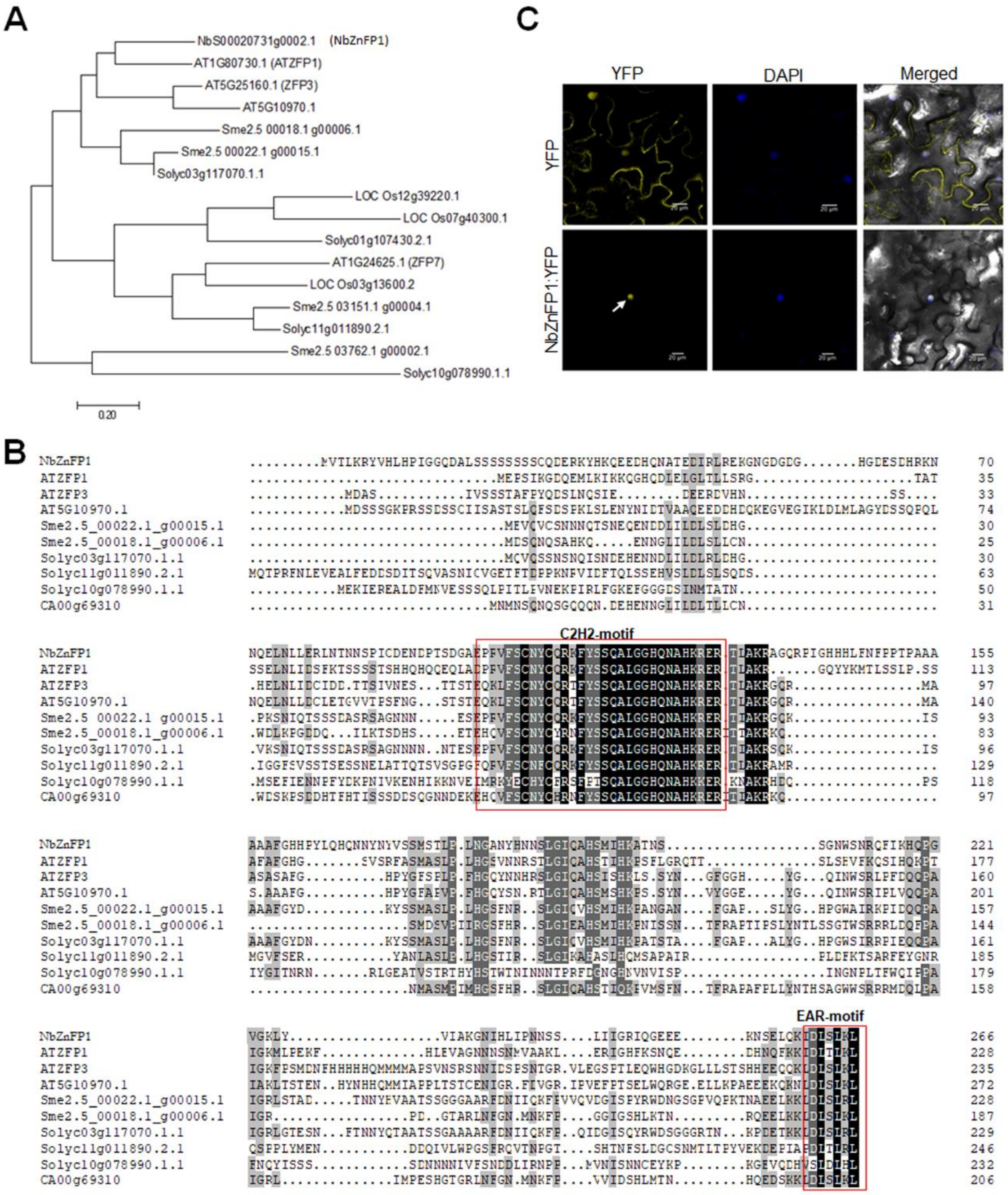

Figure S6. Phylogenetic relatedness of NbZnFP1 with related zinc finger proteins (ZnFPs) and localization of NbZnFP1 in *N. benthamiana*. (A) Relatedness of NbZnFP1 in *N. benthamiana* to ZnFPs in *Arabidopsis thaliana* (ATZFP1, ZFP3, ZFP7), *Solanum melongena*, *S. lycopersicum*, and *Capsicum annuum*. Phylogenetic relationships were based on full-length amino acid sequence data., and the tree was generated with MEGA 7.0.14. **(B)** Alignment of the NbZnFP1 sequence with orthologous proteins in *A. thaliana*, *S. melongena*, *S. lycopersicum,* and *C. annuum* with DNAMAN (https://www.lynnon.com/dnaman.html). Identical amino acids are indicated in white font with black background, and similar amino acids are shaded in dark or light gray. **(C)** Subcellular localization of NbZnFP1 in *N. benthamiana*. *Nb* leaves were infiltrated with *Agrobacterium* containing pYFP (control) or NbZnFP1::YFP, and images were captured at 48 hpi by confocal microscopy. Nuclei were stained with 4′,6-diamidino-2-phenylindole (DAPI). The white arrow indicates the nucleus. Scale bars: 20 µm.

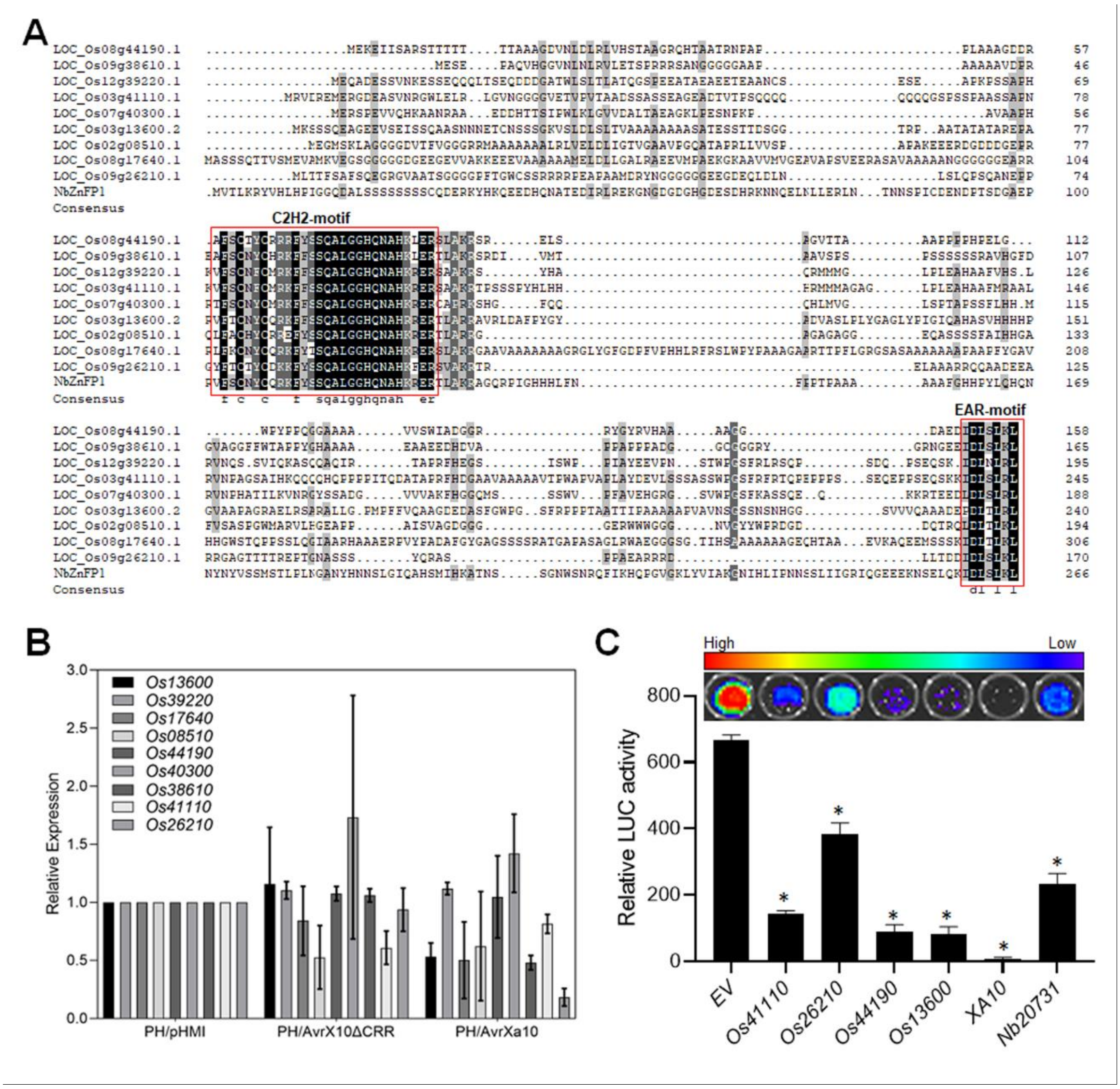

Figure S7. Alignment of NbZnFP1 orthologues in rice and expression assays. (A) Alignment of NbZnFP1 with nine orthologous proteins in rice cv. Nipponbare with DNAMAN. Proteins included: Os08g44190.1, Os9g38610.1, Os12g39220.1, Os03g41110.1, Os07g40300.1, Os03g13600.2, Os02g08510.1, Os08g17640.1, Os09g26210.1, and NbZnFP1. Identical amino acids are indicated in white font with black background; similar amino acids are shaded in dark or light gray. The C2H2 and EAR motifs are shown. **(B)** Expression of *NbZnFP1* homologs in rice inoculated with the derivatives of the *tal*-free strain, *Xoo* PH (Table S2). Leaves of three-week-old rice plants were infiltrated with PH strains carrying AvrXa10, AvrXa10ΔCRR, or pHM1 (empty vector), and collected at 24 hpi for RNA isolation. *OsActin* was used as an internal control. Error bars represent means and standard deviations (means ± SD) (n =3), and columns labeled with asterisks represent significant differences (P<0.05). **(C)** Transient expression of four *NbZnFP1* orthologue from rice cause cell death in rice protoplasts. Constructs pRTVcHA-Os41110, pRTVcHA-Os26210, pRTVcHA-Os44190, pRTVcHA-Os13600 and pRTVcHA (EV, empty vector) were co-expressed with the LUC reporter construct, pRTVcVC-LUC, in rice protoplasts. Co-transfection of pRTVcHA-Xa10 (Xa10) with the LUC construct was used as a positive, cell-death inducing control. LUC activity was measured after 24 h of transfection using Promega LUC assay system (Promega Corp.). The images on the top of the graph show microtiter plates containing protoplasts expressing the constructs. The image of LUC fluorescence was taken with CCD imaging (IVIS spectrum, PerkinElmer, USA). The graph below show the relative LUC activity measured with luminometer (Tecan, M200). Cell death in protoplasts was monitored by reduction in luciferase activity. Error bars represent means ± SD, and columns labeled with an asterisk (*) represent significance at P<0.01.

Supplemental Tables

Table S1. The list of candidate targets of AvrXa10.

Gene ID	EBE Score[a]	EBE sequence	EBE to ATG (bp)	Log$_2$FC[b] (vs. AvrXaΔCRR)		Expression[c] (vs. AvrXaΔCRR)		Description
				32 hpi	48 hpi	32 hpi	48 hpi	
NbS00029135g0002	-7.10287	TATATAAGCACATACCC	102	6.268363	8.359675	317.90	46.78	uncharacterized protein
NbS00036259g0008	-7.10287	TATATAAGCACATACCC	123	5.275453	Inf[d]	265.06	85.98	uncharacterized protein
NbS00020731g0002	-9.24073	TATATAAGCACATCTCT	77	Inf	9.240476	2.45	18.06	zinc finger protein 1-like
NbS00016355g0012	-10.0296	TATATAATCACGTGCCC	33	6.875339	8.508549	-	-	bidirectional sugar transporter SWEET6a-like
NbS00003692g0004	-10.7409	TATATAAGCACGTCTTC	16	6.282507	4.117477	1.98	0.36	transcription factor bHLH87-like
NbS00045656g0001	-11.0257	TATATAAGCACATCTAC	81	3.562645	4.422442	21.22	2.03	ethylene-responsive transcription factor 4-like
NbS00008774g0001	-11.2694	TATATAAGCATGTCTCT	115	4.509918	5.534762	1.20	0.14	transcription repressor OFP12-like
NbS00044252g0001	-11.4589	TATATACACACATCCCC	9	5.418556	8.997659	48.65	7.46	GDSL esterase/lipase At3g26430-like
NbS00004975g0004	-11.6268	TATATAAACACCAATCT	157	4.509345	6.526783	8.47	7.79	small auxin-up protein 58
NbS00036496g0001	-11.6283	TATATATACACATATCC	23	7.130504	8.262867	3.67	2.45	uncharacterized protein

[a]EBE score calculated via TALgetter tool; [b]Log$_2$-fold change values from RNA-seq data; [c]Expression values by qRT-PCR analysis; [d]Infinity value

Table S2. Bacterial strains and plasmids used in this study.

Strain or plasmid	Relevant characteristics	Source
**Strain**		
Escherichia coli		
DH5α	*F-*, *endA1*, *thi-1*, *recA1*, Φ80*lacZ*, Δ M15	Clontech
BL21	*F-*, *ompT*, *hsdS20*, *gal*, *dcm*(DE3)	Novagen
Agrobacterium tumefaciens		
EHA105	C58, pTiBo542DT-DNA, Rif^r	This lab
GV3101	C58, pTic58DT-DNA, Rif^r	This lab
Xanthomonas oryzae* pv*. oryzae		
PH	*tal*-free derivative of Xoo PXO99^A	(Ji et al., 2016)
**Plasmids**		
pHB	Binary vector, double 35S promoter, 3X N-terminal FLAG tag, Km^r	(Mao et al., 2005)
pUC57	Cloning vector, pUC19 derivative, Ap^r	ViewSolid Biotechnology, Beijing
pRTVcHA	*Ubi* promoter, c-terminal 4X-HA tag; used for transient expression in rice	(He et al., 2018)
pRTVcVC-LUC	*LUC* in pRTVcVC, mCherry, C-terminal cMyc-tag + mVenus-$C_{156-238}$	(Xu et al., 2021)
pET30a(+)	pBR322 origin, *lacI*, His-tag at C-terminus, Km^r	Novagen
pCAMBIA1381	Binary vector containing promoterless *gusA*; used for reporter assays, Km^r	
pTRV-RNA1 (pTRV1)	pTRV encoding replicase, movement protein and cysteine-rich protein, helper vector, Km^r	(Zhao et al., 2013)
pYL156-RNA2 (pTRV2)	TRV-based VIGS vector, Km^r	(Zhao et al., 2013)
pHM1	Broad-spectrum cosmid vector, Sp^r	(Hopkins et al., 1992)

pYFP	Binary vector with full-length YFP coding gene, c-Myc tag, Km^r	(Ma et al., 2018)
pZW-avrXa10	*avrXa10* in pBluescript II KS^+, contains FLAG epitope immediately downstream of the second *Sph*I site in the C-terminus of AvrXa10, Ap^r	(Zhu et al., 1998)
pHB-Hpa1	*hpa1* cloned in pHB	This study
pHB-hpa1	*hpa1* cloned in frame with N-terminal flag-tag, Km^r	(Ma et al., 2020)
pHB-AvrXa10	*avrXa10* cloned in frame with N-terminal flag-tag in pHB, Km^r	This study
pHB-AvrXa7	*avrXa7* cloned with N-terminal flag-tag in pHB, Km^r	This study
pHB-AvrXa27	*avrXa27* cloned with N-terminal flag-tag in pHB, Km^r	This study
pHB-PthXo1	*pthXo1* cloned with N-terminal flag-tag in pHB, Km^r	This study
pHB-pthXo7	*pthXo7* cloned with N-terminal flag-tag in pHB, Km^r	This study
pHB-tal2a	*tal2a* cloned with N-terminal flag-tag in pHB, Km^r	This study
pHB-tal4	*tal4* cloned with N-terminal flag-tag in pHB, Km^r	This study
pHB-tal5a	*tal5a* cloned with N-terminal flag-tag in pHB, Km^r	
pHB-pthXo6	*pthXo6* cloned with N-terminal flag-tag in pHB, Km^r	This study
pHB-tal6a	*tal6a* cloned with N-terminal flag-tag in pHB, Km^r	This study
pHB-tal7a	*tal7a* cloned with N-terminal flag-tag in pHB, Km^r	This study
pHB-tal7b	*tal7b* cloned with N-terminal flag-tag in pHB, Km^r	This study
pHB-tal8a	*tal8a* cloned with N-terminal flag-tag in pHB, Km^r	This study
pHB-tal8b	*tal8b* cloned with N-terminal flag-tag in pHB, Km^r	This study
pHB-pthXo8	*pthXo8* cloned with N-terminal flag-tag in pHB, Km^r	This study
pHB-avrXa23	*avrXa23* cloned with N-terminal flag-tag in pHB, Km^r	This study
pHB-tal9d	*tal9d* cloned with N-terminal flag-tag in pHB, Km^r	This study
pHB-tal9e	*tal9e* cloned with N-terminal flag-tag in pHB, Km^r	This study
pHB-AvrXa10ΔCRR	pHB containing N and C-terminal regions of AvrXa10; lacks *Sph*I fragment containing the CCR, Km^r	This study
pHB-AvrXa10ΔAD	pHB containing a truncated *avrXa10* that lacks the AD domain; Km^r	This study
pHB-$dTALE_{20731}$	*$dTALE_{20731}$* cloned in pHB	This study
pHB-$dTALE_{44252}$	*$dTALE_{44252}$* cloned in pHB	This study
pHB-$dTALE_{45656}$	*$dTALE_{45656}$* cloned in pHB	This study
pHB-NbZnFP1	*NbZnFP1* cloned in pHB	This study

pET30a-pthXo1	*pthXo1* cloned in pET30a; contains His-tag at C-terminus, Km^r	(Ma et al., 2018)
pET30a-avrXa10	*avrXa10* cloned in pET30a; contains His-tag at C-terminus, Km^r	This study
pHZW-AvrXa10	pHM1 fused with pZWavrXa10 at *Hin*dIII, *lacZ* promoter upstream of *avrXa10*, Ap^r, Sp^r	(Zhu et al., 1998)
pZW- AvrXa10ΔCRR	*Sph*I central repeat region was deleted in pZW-avrXa10 and self-ligated, Ap^r	This study
pHZW-AvrXa10ΔCRR	pHM1 fused with pZWavrXa10ΔCRR at *Hin*dIII, *lacZ* promoter upstream of *avrXa10ΔCRR*, Ap^r, Sp^r	This study
p29135g::GUS	~1 kb promoter region of *29135g* cloned upstream of *gusA* in pCAMBIA1381	This study
p36259g::GUS	~1 kb promoter region of *36259g* cloned upstream of *gusA* in pCAMBIA1381	This study
p20731g::GUS	~1 kb promoter region of *20731g* cloned upstream of *gusA* in pCAMBIA1381	This study
p44252g::GUS	~1 kb promoter region of *44252g* cloned upstream of *gusA* in pCAMBIA1381	This study
p45656g::GUS	~1 kb promoter region of *45656g* cloned upstream of *gusA* in pCAMBIA1381	This study
p3692g::GUS	~1 kb promoter region of *3692g* cloned upstream of *gusA* in pCAMBIA1381	This study
pOs8N3::GUS	promoter region of *Os8N3* cloned upstream of *gusA* in pCAMBIA1381	(Cai et al., 2017)
pOs8N3::29135	*29135g* CDS in pCAMBIA1381, driven by *Os8N3* promoter, Km^r	This study
pOs8N3::36259g	*36259g* CDS in pCAMBIA1381, driven by *Os8N3* promoter, Km^r	This study
pOs8N3::20731g	*20731g* CDS in pCAMBIA1381, driven by *Os8N3* promoter, Km^r	This study
pOs8N3::44252g	*44252g* CDS in pCAMBIA1381, driven by *Os8N3* promoter, Km^r	This study
pOs8N3::45656g	*45656g* CDS in pCAMBIA1381, driven by *Os8N3* promoter, Km^r	This study

pOs8N3::3692g	*3692g* CDS in pCAMBIA1381, driven by *Os8N3* promoter, Km^r	This study
pOs8N3::hpa1	*hpa1* CDS in pCAMBIA1381, driven by *Os8N3* promoter, Km^r	This study
pOs8N3::EV	*Os8N3* promoter cloned in pCAMBIA1381, Km^r	This study
pYL156-NbZnFP1	Partial CDS of *NbZnFP1* (*Nb20731g*) cloned as a *Xba*I/*Bam*HI fragment in pYL156	This study
pGBKT7-20731g	*20731g* gene cloned in pGBKT7	This study
pRTVcHA-NbZnFP1	Tobacco gene *NbZnFP1* (*Nb20731g*) cloned as a *Bam*HI/*Not*I fragment in pRTVcHA	This study
pRTVcHA-Xa10	Rice gene *Xa10* cloned as a *Bam*HI/*Not*I fragment in pRTVcHA	This study
pRTVcHA-Os41110	Rice gene *LOC_Os03g41110* cloned as a *Bam*HI/*Not*I fragment in pRTVcHA	This study
pRTVcHA-Os26210	Rice gene *LOC_Os09g26210* cloned as a *Bam*HI/*Not*I fragment in pRTVcHA	This study
pRTVcHA-Os44190	Rice gene *LOC_Os08g44190* cloned as a *Bam*HI/*Not*I fragment in pRTVcHA	This study
pRTVcHA-Os13600	Rice gene *LOC_Os03g13600* cloned as a *Bam*HI/*Not*I fragment in pRTVcHA	This study
pB-Xa10	Rice *Xa10* gene in pHB vector	Lab collection
NbZnFP1-YFP	*NbZnFP1* in *Kpn*I/*Sma*I site of pYFP, c-myc tag, Km^r	This study

Abbreviations: Ap, ampicillin; Sp, spectinomycin; Km, Kanamycin; Rif, Rifampicin

Table S3. Primers used in this study.

Primer name	Sequence (5'-3'; restriction sites underlined)	Description
avrXa10-F	CCCAAGCTTATGGATCCCATTCGTT	Amplifies N-terminus of AvrXa10 for CRR mutant construction; contains *Hin*dIII/*Sph*I sites
avrXa10-N-R	TGCATTGCGCCATGCATGCACTG	
avrXa10-C-F	CGGATCAGGCGTCTTTGCATGCA	Amplifies C-terminus of AvrXa10 for CRR mutant; contains *Sph*I/*Xba*I sites
avrXa10-R	GCTCTAGATCAGATCGTCCCTCCG	
avrXa10-(ΔAD)-F	GCGTCGACCCAGCCCAATG	Amplifies 176 bp from C-terminus of AvrXa10; for AD mutant; contains *Sal*I/*Xba*I sites
avrXa10-C(ΔAD)-R	GCTCTAGATCAGGTACGCGGGCGTTTTAC	
NbPR1a-F	GGTGTAGAACCTTTGACCTGGG	qPCR primer for *NbPR1a*
NbPR1a-R	AAATCGCCACTTCCCTCAGC	
NbPR2-qRT-F	TAGAGAATACCTACCCGCCC	qPCR primer for *NbPR2*
NbPR2-qRT-R	GAGTGGAAGGTTATGTCGTGC	
NbPR4-qRT-F	GTGACGAACACAAGAACAGGAA	qPCR primer for *NbPR4*
NbPR4-qRT-R	CCACTCCATTTGTGTCCAAT	
NbPR5-qRT-F	TTATGGTGAGACTGGACTCCC	qPCR primer for *NbPR5*
NbPR5-qRT-R	CCACCAGATGCCTTCTTTG	
NbPR10-qRT-F	AGGAGTCAGGTGATGGTGGT	qPCR primer for *NbPR10*
NbPR10-qRT-R	TGAACATAGCCGAGACCTTC	
NbNPR1-qRT-F	TGAGATTCTGGAGCAAGCA	qPCR primer for *NbNPR1*
NbNPR1-qRT-R	GCTTCATACGCAAATCATCG	
NbPTI-qRT-F	AGGCGTAAGACGGAGACCAT	qPCR primer for *NbPTI*
NbPTI-qRT-R	CCTTAGCACCACGCATTCTA	
NbEDS1-qRT-F	TTGGGCAGAAGTGGAGGAAC	qPCR primer for *NbEDS1*
NbEDS1-qRT-R	AAACATCATCGCCCAGAAGGC	
NbEF1α-qRT-F	AGACCACCAAGTACTACTGCAC	qPCR primer for *NbEF1α*; used as a reference gene for qRT-PCR
NbEF1α-qRT-R	CCACCAATCTTGTACACATCC	
Xa10-F	CGGGATCCATGCAGCTGATGCTCACATTC	Amplifies *Xa10* for cloning into pRTVcHA vector; contains *Bam*HI/*Not*I sites
Xa10-R	TTGCGGCCGCGACGGGGGAAATCTCCTC	
29135g-qRT-F	GGACAACAAGTTCACTAGCCTATTTG	qPCR primer for *29135g*
29135g-qRT-R	AACTTCTCCAAAATAGTAACTCTGATCAAT	
36259g-qRT-F	GGACAACAAGTTCACTAGCCTATCTC	qPCR primer for *36259g*
36259g-qRT-R	CAACTTCTCCAATATAGTAACTCTGATCAAC	
20731g-qRT-F	ATCCAACATCAGATGGTGCAGAG	qPCR primer for *20731g*

20731g-qRT-R	CCAGCTCTTTTGGCAAGTGTTC	
3692g-qRT-F	CAACAAGGACAATATTGCAGTTTCAT	qPCR primer for *3692g*
3692g-qRT-R	TGATCCGTCTCATTAATAGGGCAC	
45656g-qRT-F	GAGGAGTAAGAAAGAGGCCATGG	qPCR primer for *45656g*
45656g-qRT-R	CGGCGACGTCATAAGCCC	
20731g-F	CGGGATCCATGGTAACCCTAAAAAGATATGTACATT	Amplifies *Nb20731g* for cloning into pRTVcHA vector; contains *Bam*HI/*Not*I sites
20731g-R	TTGCGGCCGCGAGTTTGAGAGAAAGATCGATCTTT	
Os13600-F	CGGGATCCATGAAGAGCAGCAGCCAAGAAG	Amplifies *LOC_Os03g13600* for cloning into pRTVcHA vector; contains *Bam*HI/*Not*I sites
Os13600-R	TTGCGGCCGCGAGTCTAAGTGTGAGGTCGGGCT	
Os41110-F	CGGGATCCATGCGAGTCGATAGGGAGAT	Amplifies *LOC_Os03g41110* for cloning into pRTVcHA vector; contains *Bam*HI/*Not*I sites
Os41110-R	TTGCGGCCGCCAGCCTGAGGCTCAAGTCTATC	
Os26210-F	CGGGATCCATGCTCACCACTTTCTCGG	Amplifies *LOC_Os09g26210* for cloning into pRTVcHA vector; contains *Bam*HI/*Not*I sites
Os26210-R	TTGCGGCCGCTAGCTTCAGGGACAGGTCG	
Os44190-F	CGGGATCCATGGAGAAGGAGATCATCAGC	Amplifies *LOC_Os08g44190* for cloning into pRTVcHA vector; contains *Bam*HI/*Not*I sites
Os44190-R	TTGCGGCCGCCAACTTGAGAGAAAGGTCGATGT	
8774g-qRT-F	CTTCCCAAACTTTAAATGCCTTC	qPCR primer for *8774g*
8774g-qRT-R	TTAGTGAAAGATTTAGAAGTGGTGGA	
44252g-qRT-F	TAAGAGCAAGTGTGATTTTCCGG	qPCR primer for *44252g*
44252g-qRT-R	GGCAGGAGCACCAAAATATGTC	
4975g-qRT-F	AATTTCCATGTTCAAGTGCATCTAC	qPCR primer for *4975g*
4975g-qRT-R	GACCTACAAATACTGGAAGACACCC	
36496g-qRT-F	CACCGAGAATGGTAGATCAAAGG	qPCR primer for *36496g*
36496g-qRT-R	CAGCCTGAAAAAATCAACCCC	
p29135g-F	GGAATTCCTAGTCTTAGCAAGTTCTTTCTTTTTG	Amplifies ~1 kb promoter region of *29135g* for cloning in pCAMBIA1381; contains *Eco*RI/*BamH*I sites
p29135g-R	CGGGATCCGAAATAAGTATAATTAAGTCTGTCGAGGAG	
p36259g-F	GGAATTCGTGCTTACTTGTGTATTTTTCCCC	Amplifies ~1 kb promoter region of *36259g* for cloning into pCAMBIA1381; contains *Eco*RI/*Bam*HI sites
p36259g-R	CGGGATCCGAAATAAGTATAATTAAGTTTGTCGATG	
p44252g-F	GGAATTCACTACATAGCTGGGGAATGGG	Amplifies ~1 kb promoter region of *44252g* for cloning into pCAMBIA1381; contains *Eco*RI/*Bam*HI sites
p44252g-R	CGGGATCCATGACTATATATTCTACAAAGCAAAGATGC	

p45656g-F	GGAATTCGAAGGCAGCAATACGAATATTAGAAG	Amplifies ~1 kb promoter region of *45656g* for cloning into pCAMBIA1381; contains *Eco*RI/*Bam*HI sites
p45656g-R	CGGGATCCTTTTGTATAGCTGATATGGTGTATTTTGTG	
29135g-F	AACTGCAGATGGATCGCGCACTAATACTC	Amplifies *29135g* for cloning into pHB; contains *Pst*I/*Sac*I sites
29135g-R	CGAGCTCTTATTCTTTCCATGGAAGTGCA	
36259g-F	CGAGCTCATGGCTCGCGCACTAGC	Amplifies *36259g* for cloning into pHB; contains *Sac*I sites
36259g-R	CGAGCTCTCAACCTTTTGCTGATGAAGAG	
20731g-F	AACTGCAGATGGTAACCCTAAAAAGATATGTACATTTAC	Amplifies *20731g* for cloning into pHB; contains *Pst*I/*Sac*I sites
20731g-R	CGAGCTCTTAGAGTTTGAGAGAAAGATCGATCTTT	
44252g-F	AACTGCAGATGGAGCTTAGTGTCTCAAAACTAG	Amplifies *44252g* for cloning into pHB; contains *Pst*I/*Sac*I sites
44252g-R	CGAGCTCTCACTTGTGACATGCCATGTTC	
45656g-F	AACTGCAGATGGCTATGAAAGAAAAGCTAAATG	Amplifies *45656g* for cloning into pHB; contains *Pst*I sites
45656g-R	AACTGCAGTCAAGCTTCCATTGGTGGG	
3692g-F	AACTGCAGATGGAATTTGGAGCTACATTTGAT	Amplifies *3692g* for cloning into pHB; contains *Pst*I/*Sac*I sites
3692g-R	CGAGCTCTTAAGTATTCTTACCTGGTTGGAGC	
29135g-F	ttaagaggagtccacCATGGATCGCGCACTAATACTC	Amplifies *29135g* for cloning into pCAMBIA1381 by replacing *gusA*
29135g-R	gtcaccaattcacacgtgatggtgatggtgatgTTCTTTCCATGGAAGTGCA	
36259g-F	ttaagaggagtccacCATGGCTCGCGCACTAGC	Amplifies *36259g* for cloning into pCAMBIA1381 by replacing *gusA*
36259g-R	gtcaccaattcacacgtgatggtgatggtgatgTTCTTTCCATGGAAGTGCAA	
20731g-F	CATGCCATGGTAACCCTAAAAAGATATGTACATTTAC	Amplifies *20731g* for cloning into pCAMBIA1381 by replacing *gusA*
20731g-R	ACCCACGTGatggtgatggtgatgGAGTTTGAGAGAAAGATCGATCTTT	
44252g-F	CATGCCATGGAGCTTAGTGTCTCAAAACTAG	Amplifies *44252g* for cloning into pCAMBIA1381 by replacing *gusA*
44252g-R	ACCCACGTGatggtgatggtgatgCTTGTGACATGCCATGTTC	
45656g-F	ttaagaggagtccacCATGGCTATGAAAGAAAAGCTAAATG	Amplifies *45656g* for cloning into pCAMBIA1381 by replacing *gusA*
45656g-R	gtcaccaattcacacgtgatggtgatggtgatgAGCTTCCATTGGTGGG	
3692g-F	ttaagaggagtccacCATGGAATTTGGAGCTACATTTGAT	Amplifies *3692g* for cloning into pCAMBIA1381 by replacing *gusA*
3692g-R	gtcaccaattcacacgtgatggtgatggtgatgAGTATTCTTACCTGGTTGGAGC	

20731g(EBE)-F	CTAATATATATAAGCACATCTCTTCCTCC	EBE fragment for EMSA
20731g(EBE)-R	GGAGGAAGAGATGTGCTTATATATATTAG	
44252g(EBE)-F	GTATATATATACACACATCCCCCATTTAAC	EBE fragment for EMSA
44252g(EBE)-R	GTTAAATGGGGGATGTGTGTATATATATAC	
45656g(EBE)-F	CTCCATTATATAAGCACATCTACACATC	EBE fragment for EMSA
45656g(EBE)-R	GATGTGTAGATGTGCTTATATAATGGAG	
NbZnFP1-F	AACTGCAGATGGTAACCCTAAAAAGATATGTACATTTAC	Amplifies *NbZnFP1* for cloning into pHB vector; contains *PstI*/*Sac*I sites
NbZnFP1-R	CGAGCTCTTAGAGTTTGAGAGAAAGATCGATCTTT	
NbZnFP1(VIGS)-F	GCTCTAGAACCCCATAGGTGGTCAAGAC	Used for on-spot silencing; amplifies 285 bp of *NbZnFP1* for VIGS construct; contains *Xba*I/*Bam*HI restriction sites
NbZnFP1(VIGS)-R	CGGGATCCTGCACGAGAATACTCTTGGC	
NbZnFP1-YFP-F	GGGGTACCATGGTAACCCTAAAAAGATATGTACATTTAC	Amplifies *NbZnFP1* for cloning into pYFP vector; contains *Kpn*I/*Sma*I sites
NbZnFP1-YFP-R	TCCCCCGGGGAGTTTGAGAGAAAGATCGATCTTTTG	
Solyc03g117070(qRT)-F	ATCTACGCCTCGATCACGG	qPCR primer for *Solyc03g117070*
Solyc03g117070(qRT)-R	GCGAACTGTAAAATTTCCTCTGG	
Solyc07g006880(qRT)-F	ATGAGTTATGAACCAAACACGGC	qPCR primer for *Solyc07g006880*
Solyc07g006880(qRT)-R	GCAAGAAAAAACACGTGGCTC	
Solyc00g014800(qRT)-F	TGTTTACGATTTCTGGAACCAGTC	qPCR primer for *Solyc00g014800*
Solyc00g014800(qRT)-R	AAACTTGCGAGAACAGTAGAGGC	
Solyc10g078990(qRT)-F	GAGTCCTCCTCTCAACTTCCTATTAC	qPCR primer for *Solyc10g078990*
Solyc10g078990(qRT)-R	TTTCCTCATGATTTCAACATTCTTC	
SolyEF1α(qRT)-F	GATTGACAGACGTTCTGGTAAGGA	qPCR primer for *SolyEF1α*
SolyEF1α(qRT)-R	ACCGGCATCACCATTCTTCA	
Os03g13600(qRT)-F	TGAAGAGCAGCAGCCAAGAAG	qPCR primer for *Os03g13600*
Os03g13600(qRT)-R	GTGAGCGACAGGTCGAGGC	
Os12g39220(qRT)-F	CGAGTCGTCGGTGAACAAAGAG	qPCR primer for *Os12g39220*

Os12g39220(qRT)-R	GTTTGCTGCTTCTGTTTCTTCTGC	
Os08g17640(qRT)-F	CTTCCAGCTCGCAGACCACC	qPCR primer for *Os08g17640*
Os08g17640(qRT)-R	CTCCTCCTCCTTCTTGGCCAC	
Os02g08510(qRT)-F	AGGAGGAGGCGACGTGACG	qPCR primer for *Os02g08510*
Os02g08510(qRT)-R	GACACGACGAGCAGACGAGG	
Os08g44190(qRT)-F	ATGGAGAAGGAGATCATCAGCG	qPCR primer for *Os08g44190*
Os08g44190(qRT)-R	AGCCGCAGGTCGAGGTTG	
Os07g40300(qRT)-F	AAGTGGTGCAACACAAGGCAG	qPCR primer for *Os07g40300*
Os07g40300(qRT)-R	GGCTTCGGATTCGACTCG	
Os09g38610(qRT)-F	GGCGGCGTCAACCTCAAC	qPCR primer for *Os09g38610*
Os09g38610(qRT)-R	GGCAGTAGTTGCAGGAGAAGGC	
Os03g41110(qRT)-F	CGATAGGGAGATGGAGAGAGGG	qPCR primer for *Os03g41110*
Os03g41110(qRT)-R	AAGGAGAACCCTGCTGCTGC	
Os09g26210(qRT)-F	ATGCTCACCACTTTCTCGGC	qPCR primer for *Os09g26210*
Os09g26210(qRT)-R	GAGGTCCAGCTGCTCATCCC	
OsActin(qRT)-F	GTTCCTGCTGTTTGTTCTGTTG	qPCR primer for *OsActin*
OsActin(qRT)-R	ATCTCACGCATTACCCTACCTT	

Supplemental Dataset 1. Script for promoter extraction from *N. benthamiana* genome

Supplemental Dataset 2: List of genes up-regulated by AvrXa10 in *Nb* and putative target genes containing EBEs recognized by AvrXa10.

Supplemental References

Cai, L., Cao, Y., Xu, Z., Ma, W., Zakria, M., Zou, L., Cheng, Z., and Chen, G. (2017). A transcription activator-like effector Tal7 of *Xanthomonas oryzae* pv. *oryzicola* activates rice gene *Os09g29100* to suppress rice immunity. Sci. Rep **7**:5089.

Haq, F., Xie, S., Huang, K., Shah, S.M.A., Ma, W., Cai, L., Xu, X., Xu, Z., Wang, S., and Zou, L. (2020). Identification of a virulence *tal* gene in the cotton pathogen, *Xanthomonas citri* pv. *malvacearum* strain Xss-V_2–18. BMC Microbiol. **20**:1-13.

He, F., Zhang, F., Sun, W., Ning, Y., and Wang, G. (2018). A versatile vector toolkit for functional analysis of rice genes. Rice **11**:1-10.

Hopkins, C.M., White, F., Choi, S., Guo, A., and Leach, J. (1992). Identification of a family of avirulence genes from *Xanthomonas oryzae* pv. *oryzae*. Mol. Plant-Microbe Interact. **5**:451-459.

Ji, Z., Ji, C., Liu, B., Zou, L., Chen, G., and Yang, B. (2016). Interfering TAL effectors of *Xanthomonas oryzae* neutralize R-gene-mediated plant disease resistance. Nat. Commun **7**:1-9.

Ma, W., Zou, L., Ji, Z., Xu, X., Xu, Z., Yang, Y., Alfano, J.R., and Chen, G. (2018). *Xanthomonas oryzae* pv. *oryzae* TALE proteins recruit OsTFIIAγ1 to compensate for the absence of OsTFIIAγ5 in bacterial blight in rice. Mol. Plant Pathol. **19**:2248-2262.

Ma, W., Xu, X., Cai, L., Cao, Y., Haq, F., Alfano, J.R., Zhu, B., Zou, L., and Chen, G. (2020). A *Xanthomonas oryzae* type III effector XopL causes cell death through mediating ferredoxin degradation in *Nicotiana benthamiana*. Phytopathology Res. **2**:1-12.

Mao, J., Zhang, Y., Sang, Y., Li, Q., and Yang, H. (2005). A role for *Arabidopsis* cryptochromes and COP1 in the regulation of stomatal opening. Proc. Natl. Acad. Sci. USA **102**:12270-12275.

Xu, X., Xu, Z., Ma, W., Haq, F., Li, Y., Shah, S.M.A., Zhu, B., Zhu, C., Zou, L., and Chen, G. (2021). TALE-triggered and iTALE-suppressed *Xa1* resistance to bacterial blight is independent of *OsTFIIAγ1* or *OsTFIIAγ5* in rice. J. Exp. Bot.

Zhao, Y., Liu, W., Xu, Y.-P., Cao, J.-Y., Braam, J., and Cai, X.-Z. (2013). Genome-wide identification and functional analyses of calmodulin genes in *Solanaceous* species. BMC Plant Biol. **13**:1-15.

Zhu, W., Yang, B., Chittoor, J.M., Johnson, L.B., and White, F.F. (1998). AvrXa10 contains an acidic transcriptional activation domain in the functionally conserved C terminus. Mol. Plant-Microbe Interact. **11**:824-832.
