# Supplementary material for: A Xanthomonas transcription activator-like effector is trapped in nonhost plants for immunity
Source: Plant Commun. 2021 Oct 14;3(1):100249. doi: 10.1016/j.xplc.2021.100249 (PMC8760140; doi:10.1016/j.xplc.2021.100249)
Supplement: Supplemental Data 1. Script for promoter extraction from N. benthamiana genome [file mmc2.docx]

**Script for promoter extraction from *N. benthamiana* genome (v0.4.4)**

________________________________________________________________________

#!/usr/bin/perl

##########使用：perl XX.pl XXX.gb tag. tag用于指定输出文件的前缀。

use warnings;

use strict;

my $filename = shift @ARGV;

my $out_tag = shift @ARGV;

my $outNuc = $out_tag."_promter_region.txt";

open FILE, '<', $filename

or die "Can't open '$filename': $!";

open(OUT1, ">$outNuc");

my $strain;

my ($contig, $contig_seq);

my (@complement, @locus, @locus_tag,@product,@protein_id,@GeneID);

my $gene_num =0;

my ($sig1,$sig2,$sig3,$sig5);

my $in;

while (<FILE>) {

s/\n//;

my @in;

if (/CDS\s+(complement\()?(join\()?\d+.+(\))?(\))?/) { ##########换行问题还没处理 使用$sig2

$sig1=1;

$gene_num++;

s/CDS\s+//;

unless (/\,/) {

if (s/complement\(//) {$complement[$gene_num]=1;

} else {$complement[$gene_num]=0;}

s/join\(//;

s/\)//g;

s/\s+//;

@in = split /\.\.(\>)?/;

if ($complement[$gene_num]) {

$locus[$gene_num]=$in[-1];

} else {

$locus[$gene_num]=$in[0];

}

} elsif (/\)/) {

if (s/complement\(//) {$complement[$gene_num]=1;

} else {$complement[$gene_num]=0;}

s/join\(//;

s/\)//g;

s/\s+//;

@in = split /\.\.(\>)?/;

if ($complement[$gene_num]) {

$locus[$gene_num]=$in[-1];

} else {

$locus[$gene_num]=$in[0];

}

} else {

$in = $_;

$sig2=1;

}

} elsif ($sig1) {

if (s/\/gene//) {

@in = split /"/;

$locus_tag[$gene_num] = $in[1];

$sig2=0;

} elsif (s/\/product//) {

@in = split /"/;

$product[$gene_num]=$in[1];

$sig3=1;

$sig3=0 if ($_=~ s/"/#/g)==2;

} elsif (s/\/protein_id//) {

@in = split /"/;

$protein_id[$gene_num]=$in[1];

$sig3=0;

} elsif (/GeneID/) {

@in = split /"/;

$GeneID[$gene_num]=$in[1];

$sig1=0;

} else {

if ($sig2) {

$_=~ s/\s\s+/ /;

$in .=$_;

if (/\)/) {$sig2=0;

} else { next;}

if ($in =~ s/complement\(//) {$complement[$gene_num]=1;

} else {$complement[$gene_num]=0;}

$in =~ s/join\(//;

$in =~ s/\)//g;

$in =~ s/\s+//;

@in = split /\.\.(\>)?/, $in;

if ($complement[$gene_num]) {

$locus[$gene_num]=$in[-1];

} else {

$locus[$gene_num]=$in[0];

}

} elsif ($sig3) {

$_=~ s/\s\s+/ /;

$product[$gene_num] .=$_;

if (/"/) {$sig3=0;

$product[$gene_num] =~ s/"//;

}

}

}

}

if (/ORIGIN/) {$sig5=1; next;}

if ($sig5) {

if (/\/\//) { $sig5=0;

} else {

$_=~ s/\s+//g;

$_=~ s/\d+//g;

$contig_seq .=$_;

}

}

}

foreach (1..$gene_num) {

my ($up,$down,$promter);

if ($complement[$_]) {

unless ($locus[$_]=~ /\,/) {

$down=$locus[$_]-3;

$up=$down+1999;

$promter= substr($contig_seq,$down, 2000);

$promter=~ tr/acgt/ACGT/;

$promter = reverse_complement ($promter);

print OUT1 "\>$out_tag"."_Reverse [gene=$locus_tag[$_]] [product=$product[$_]] [$GeneID[$_]] [ProteinID=$protein_id[$_]] [location=$up..$down]\n$promter\n";

} else {print "\>$out_tag"."_Reverse [gene=$locus_tag[$_]] [product=$product[$_]] [$GeneID[$_]] [ProteinID=$protein_id[$_]]\n";}

} else {

unless ($locus[$_]=~ /\,/) {

$down=$locus[$_]+1;

$up=$down-1999;

if ($up<0) {$promter= substr($contig_seq,0, $down);

} else {$promter= substr($contig_seq,$up, 2000);}

$promter=~ tr/acgt/ACGT/;

print OUT1 "\>$out_tag"."_Forward [gene=$locus_tag[$_]] [product=$product[$_]] [$GeneID[$_]] [ProteinID=$protein_id[$_]] [location=$up..$down]\n$promter\n";

} else {print "\>$out_tag"."_Reverse [gene=$locus_tag[$_]] [product=$product[$_]] [$GeneID[$_]] [ProteinID=$protein_id[$_]]\n";}

}

}

####print OUT1 "$contig_seq";

sub reverse_complement {

my $dna = shift;

# reverse the DNA sequence

my $revcomp = reverse($dna);

# complement the reversed DNA sequence

$revcomp =~tr/ACGT/TGCA/;

return $revcomp;

}
