# Supplementary material for: A Xanthomonas transcription activator-like effector is trapped in nonhost plants for immunity
Source: Plant Commun. 2021 Oct 14;3(1):100249. doi: 10.1016/j.xplc.2021.100249 (PMC8760140; doi:10.1016/j.xplc.2021.100249)
Supplement: Document S2. Article plus supplemental information [file mmc4.pdf]

Plant Communications
Research Article

CellPress
Partner Journal

A *Xanthomonas* transcription activator-like effector is trapped in nonhost plants for immunity

Fazal Haq[1,2], Xiameng Xu[1,2], Wenxiu Ma[1,2], Syed Mashab Ali Shah[1,2], Linlin Liu[1,2], Bo Zhu[1], Lifang Zou[1,2] and Gongyou Chen[1,2,*]

[1]School of Agriculture and Biology/State Key Laboratory of Microbial Metabolism, Shanghai Jiao Tong University, Shanghai, China

[2]Key Laboratory of Urban Agriculture of the Ministry of Agriculture, Shanghai, 200240, China

***Correspondence: Gongyou Chen (gyouchen@sjtu.edu.cn)**

https://doi.org/10.1016/j.xplc.2021.100249

ABSTRACT

***Xanthomonas oryzae* pv. *oryzae* (*Xoo*), the causal agent of bacterial leaf blight in rice, delivers transcription activator-like effector (TALE) proteins into host cells to activate susceptibility or resistance (*R*) genes that promote disease or immunity, respectively. Nonhost plants serve as potential reservoirs of *R* genes; consequently, nonhost *R* genes may trap TALEs to trigger an immune response. In this study, we screened 17 *Xoo* TALEs for their ability to induce a hypersensitive response (HR) in the nonhost plant *Nicotiana benthamiana* (*Nb*); only AvrXa10 elicited an HR when transiently expressed in *Nb*. The HR generated by AvrXa10 required both the central repeat region and the activation domain, suggesting a specific interaction between AvrXa10 and a potential *R*-like gene in nonhost plants. Evans blue staining and ion leakage measurements confirmed that the AvrXa10-triggered HR was a form of cell death, and the transient expression of AvrXa10 in *Nb* induced immune responses. Genes targeted by AvrXa10 in the *Nb* genome were identified by transcriptome profiling and prediction of effector binding sites. Using several approaches (*in vivo* reporter assays, electrophoretic mobility-shift assays, targeted designer TALEs, and on-spot gene silencing), we confirmed that AvrXa10 targets *NbZnFP1*, a C2H2-type zinc finger protein that resides in the nucleus. Functional analysis indicated that overexpression of *NbZnFP1* and its rice orthologs triggered cell death in rice protoplasts. An NbZnFP1 ortholog was also identified in tomato and was specifically activated by AvrXa10. These results demonstrate that *NbZnFP1* is a nonhost *R* gene that traps AvrXa10 to promote plant immunity in *Nb*.**

Key words: *Xanthomonas oryzae* pv. *oryzae*, AvrXa10, hypersensitive response, nonhost plant, zinc finger protein

Haq F., Xu X., Ma W., Shah S.M.A., Liu L., Zhu B., Zou L., and Chen G. (2022). A *Xanthomonas* transcription activator-like effector is trapped in nonhost plants for immunity. Plant Comm. **3**, 100249.

INTRODUCTION

Plants are constantly under attack by microbial pathogens in nature and cope by deploying an innate immune system to resist infection (Jones et al., 2016). The first layer of immunity is triggered by recognition of pathogen-associated molecular patterns via pattern recognition receptors; the latter are often receptor-like proteins or receptor-like kinases (Tang et al., 2017). At the cellular level, pathogen-associated molecular pattern-triggered immunity (PTI) includes the generation of reactive oxygen species (ROS), mitogen-activated protein kinase cascades, induction of pathogenesis-related (*PR*) genes, and deposition of phenolic compounds (Schwessinger and Ronald, 2012). To suppress PTI, pathogens have evolved virulence "effectors" that interfere with PTI and inhibit basal defense, resulting in effector-triggered susceptibility (Jones and Dangl, 2006; Dou and Zhou, 2012). As a countermeasure, plants have developed additional receptors that recognize effectors, resulting in a second layer of immunity known as effector-triggered immunity (ETI). ETI occurs more rapidly than PTI and is often accompanied by a hypersensitive response (HR) at the invasion site that inhibits pathogen multiplication (Jones and Dangl, 2006; Jones et al., 2016).

Bacteria in the genus *Xanthomonas* infect many important crops, including rice, wheat, cassava, soybean, and cotton. The pathogenicity of *Xanthomonas* spp. depends on the type III secretion system (T3SS) (Yang and White, 2004), which delivers effector proteins into plant cells. Effectors include *Xanthomonas* outer

Published by the Plant Communications Shanghai Editorial Office in association with Cell Press, an imprint of Elsevier Inc., on behalf of CSPB and CEMPS, CAS.

This is an open access article under the CC BY license (http://creativecommons.org/licenses/by/4.0/).

proteins or transcription activator-like effector proteins (TALEs); the latter are highly conserved in *Xanthomonas* spp. (Boch and Bonas, 2010). TALEs share a conserved modular structure that comprises a type III secretion signal at the N terminus, nuclear localization signals, an acidic activation domain (AD) at the C terminus, and a central repeat region (CRR). The nuclear localization signals guide the TALEs into the plant nucleus, where they utilize the CRR to bind specific promoter sequences known as effector-binding elements (EBE); the AD then triggers expression of the target gene (Moscou and Bogdanove, 2009; Boch and Bonas, 2010). The CRR of TALEs comprises 1.5–33.5 copies of nearly identical, tandemly arranged repeats that are mostly 33 or 34 amino acids long. These highly conserved repeats are polymorphic at positions 12 and 13, and the amino acids at these locations are known as repeat variable di-residues (RVDs). The RVDs recognize specific nucleotides in the EBE of the host gene promoter and can be used to help identify TALE targets (Boch et al., 2009; Grau et al., 2013; Cernadas et al., 2014).

Some TALEs function as virulence factors by inducing host susceptibility genes that promote plant diseases. For example, the TALE AvrBs3 from *Xanthomonas euvesicatoria* targets the pepper transcription factor *UPA20* and contributes to disease by inducing hypertrophy (Kay et al., 2007). Tal2g in *Xanthomonas oryzae* pv. *oryzicola* (*Xoc*) contributes to lesion development in rice by targeting the sulfate transporter OsSULTR3;6 (Cernadas et al., 2014). In citrus, *CsLOB1* is targeted by multiple TAL effectors and promotes bacterial growth and pustule formation (Hu et al., 2014; Li et al., 2014). The *SWEET* genes in host plants encode sugar transporters and are the most important virulence targets of TALEs (Yang et al., 2006; Antony et al., 2010; Cohn et al., 2014; Cox et al., 2017; Xu et al., 2019). Tal2 in *Xanthomonas citri* pv. *malvacearum* contributes to bacterial blight of cotton by targeting a yet-unknown susceptibility gene in cotton (Haq et al., 2020).

As a countermeasure, plants have evolved resistance (*R*) genes that “trap” TALEs and confer immunity. Some *R* genes, including *Xa10*, *Xa23*, *Xa27*, and *Xa7* in rice and *Bs3* and *Bs4C-R* in solanaceous plants, trap TALEs and exhibit TALE-dependent transcription to promote an HR (Bogdanove et al., 2010; Xue et al., 2020; Chen et al., 2021; Luo et al., 2021). The resistance spectra of *R* genes are diverse and depend on the cognate or “trapped” TALEs in the *Xanthomonas* population. For example, *Xa10*-mediated resistance is very limited owing to the absence of AvrXa10 in most *Xanthomonas oryzae pv. oryzae (Xoo)* races (Wang et al., 2017). Several rice *R* genes originated from the wild species *Oryza rufipogon* (Zhang et al., 2001; Wang et al., 2015); thus it seems possible that *Xanthomonas* TALEs may occasionally be trapped in nonhost plants.

Nonhost plants exhibit durable, broad-spectrum resistance to a wide range of phytopathogens. The mechanistic basis of nonhost resistance (NHR) is complex and involves both preformed and induced defense responses that may result in symptomless reactions or the HR (Uma et al., 2011; Senthil-Kumar and Mysore, 2013). Nonhost plants can also recognize effectors from pathogens, and this recognition can trigger ETI. For example, the effector AvrRxo1 from the rice pathogen *Xoc* was recognized by the maize *R* gene product Rxo1, and this recognition induced a nonhost defense in maize (Zhao et al., 2004). Furthermore, rice lines expressing *Rxo1* exhibited a high level of resistance to *Xoc* containing *avrRxo1* (Zhao et al., 2005), thus demonstrating that *R* genes from nonhost plants can be used in host resistance. Similarly, the effectors XopQ from *Xanthomonas* and HopQ1 from *Pseudomonas* are recognized by Roq1 in the nonhosts *Nicotiana benthamiana* (*Nb*) and *Nicotiana tabacum*, resulting in ETI (Wei et al., 2007; Schwartz et al., 2015; Schultink et al., 2017). Recently, our lab reported that the effector XopL from *Xoo* triggered an HR in *Nb* by interacting with ferredoxin (Ma et al., 2020). Collectively, these studies indicate that nonhost plants possess a repository of *R* genes that could be deployed in plant disease resistance breeding.

Xoo elicits an HR in the nonhost *Nb* (Li et al., 2012); however, the role of TALEs in NHR has not previously been investigated. In this study, 17 TALEs from *Xoo* were analyzed for their ability to elicit an HR-like response in *Nb* by *Agrobacterium*-mediated transient expression. The TALE AvrXa10 was shown to elicit an HR in *Nb*, and AvrXa10-mediated cell death was dependent on the induced expression of *NbZnFP1*, which encodes a C2H2-type zinc finger protein (ZnFP). AvrXa10 was also shown to trigger HR in tomato, likely by activating an *NbZnFP1* ortholog. These findings suggest that NbZnFP1 functions in a manner analogous to that of an *R* gene in *Nb* in response to the TALE AvrXa10.

RESULTS

AvrXa10 induces HR and immune responses in *N. benthamiana*

Xoo was previously shown to induce the HR in the nonhost *Nb* in a T3SS-dependent manner (Li et al., 2012). In this study, the role of *Xoo* TAL effectors in mediating NHR was investigated by cloning 17 *tal* genes into the binary vector pHB, which contains an N-terminal FLAG epitope tag driven by the 35S promoter (Supplemental Figure 1A and 1B). Genes encoding the TALEs were transiently expressed in *Nb* via *Agrobacterium*-mediated transformation to evaluate their ability to induce HR-like cell death (Supplemental Methods 1 and 2). The TALE AvrXa10 induced cell death at 3 days post inoculation (dpi), whereas the other 16 effectors (Supplemental Figure 1C) failed to induce an HR when expressed in *Nb*.

The truncated proteins AvrXa10ΔCRR and AvrXa10ΔAD, which lack the CRR and AD, respectively (Figure 1A), were constructed as described in Supplemental Methods 1. When the mutant constructs pHB-AvrXa10ΔCRR and pHB-AvrXa10ΔAD were transiently expressed in *Nb*, the HR was absent, suggesting that the cell death triggered by AvrXa10 occurs when the CRR and AD target an unknown *Nb* gene (Figure 1B). The HR-inducing ability of AvrXa10 was further tested by infiltrating *Nb* with *Agrobacterium* strains carrying pHB-AvrXa10 at OD_{600} 0.5, 0.2, 0.1, 0.01, and 0.001. AvrXa10 triggered an HR at OD_{600} 0.5, 0.2, and 0.1, but not at 0.01 or 0.001 (Supplemental Figure 1D). Immunoblotting confirmed that AvrXa10 was clearly detectable in leaves infiltrated with OD_{600} 0.5, 0.2, and 0.1, but not with lower values (OD_{600} 0.01 and 0.001) (Supplemental Figure 1E). Evans blue staining and ion leakage measurements confirmed that AvrXa10 elicited cell

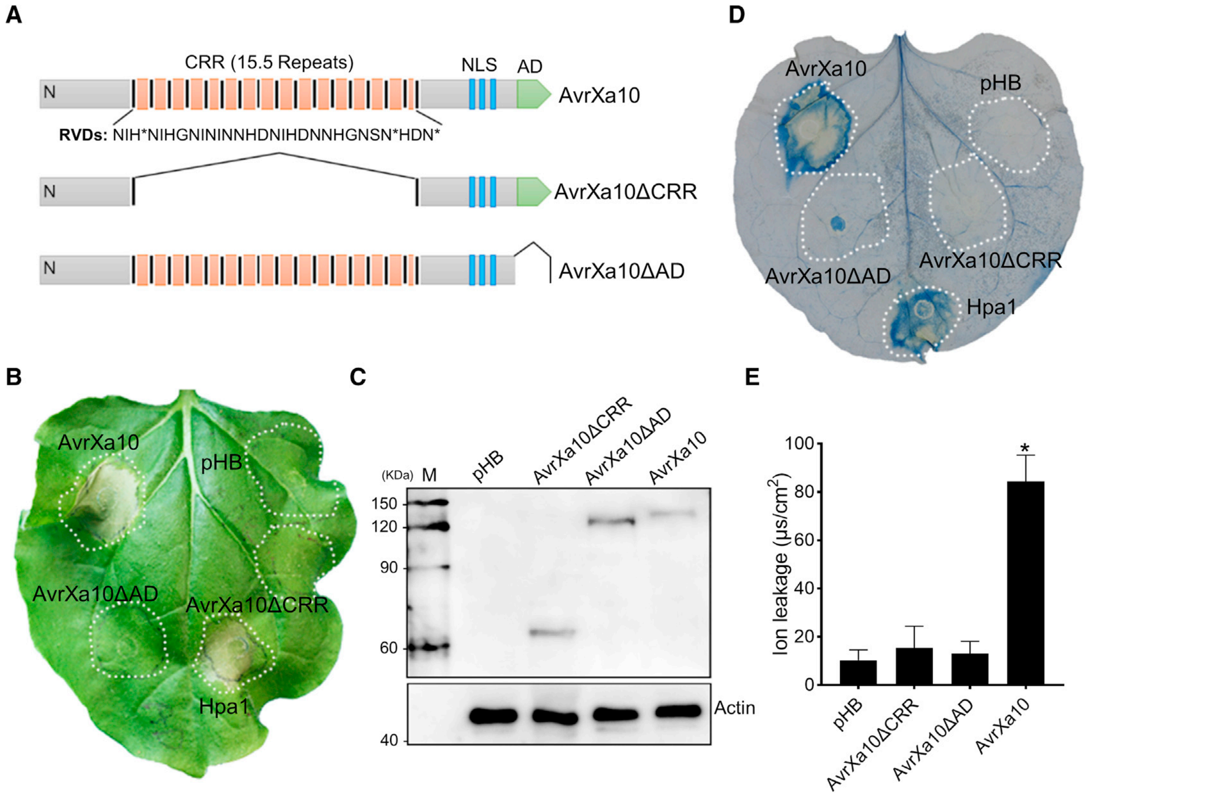

Figure 1. HR-like cell death induced by *Xoo* TALE AvrXa10 in *N. benthamiana*.

(A) Diagram showing RVDs in AvrXa10 and the construction of mutants AvrXa10ΔCRR and AvrXa10ΔAD, which lack the central repeat region (CRR) and activation domain (AD), respectively.

(B) Phenotypes of *Nb* leaves expressing AvrXa10, AvrXa10ΔCRR, and AvrXa10ΔAD. *Agrobacterium* strains containing constructs pHB-AvrXa10, pHB-AvrXa10ΔCRR, pHB-AvrXa10ΔAD, pHB-Hpa1 (positive control), and empty pHB were used to transform *Nb* leaves at OD_{600} 1.0. Photographs were taken at 3 dpi.

(C) Detection of AvrXa10, AvrXa10ΔCRR, and AvrXa10ΔAD expression in *Nb* leaves by western blotting. Actin was used as a loading control.

(D) Detection of cell death in *Nb* leaves infiltrated with *Agrobacterium* containing AvrXa10, AvrXa10ΔCRR, AvrXa10ΔAD, and empty pHB by Evans blue staining.

(E) Measurement of ion leakage in *Nb* leaves expressing AvrXa10, AvrXa10ΔCRR, AvrXa10ΔAD, or empty pHB vector. Error bars show means ± SD (n = 3), and columns labeled with an asterisk are significantly different (*$P < 0.01$) from the pHB control. The experiment was repeated at least three times with similar results.

death when transiently expressed in *Nb*, whereas AvrXa10ΔAD and AvrXa10ΔCRR did not (Figure 1D and 1E).

The cell death reaction in plants is generally preceded by other immune responses, such as the generation of ROS and the expression of defense-related genes (Asai and Yoshioka, 2009; Deb et al., 2018). Therefore, we tested whether AvrXa10 and its derivatives could induce ROS accumulation in *Nb*. *Agrobacterium* strains carrying pHB-AvrXa10, pHB-AvrXa10-ΔAD, pHB-AvrXa10ΔCRR, or empty pHB were infiltrated into *Nb* leaves, and ROS accumulation was measured. AvrXa10, but not AvrXa10ΔAD or AvrXa10ΔCRR, induced ROS accumulation at 2 and 3 dpi (Supplemental Figure 2A). Furthermore, we monitored the expression of eight defense-related genes after agroinfiltration of *Nb* leaves with pHB-AvrXa10 and pHB at 0, 32, and 46 h post inoculation (hpi) (Supplemental Figure 2B). Compared with the pHB control, genes highly induced by AvrXa10 included the *PR* genes *PR1*, *PR2*, *PR4*, *PR5*, and *PR10*; the HR marker gene *EDS1*; the master immune regulatory gene *NPR1*; and the pattern-triggered immunity gene *PTI5*. Taken together, these results indicated that AvrXa10 induced HR-like cell death and defense responses when transiently expressed in *Nb.*

Candidate targets of AvrXa10 in *N. benthamiana*

Our results indicated that the AvrXa10-mediated HR in *Nb* is dependent on the CRR and AD, implying that the AvrXa10 RVDs bind to an unknown *R* gene promoter. Two complementary approaches were used to identify putative targets of AvrXa10 (Figure 2A). In one approach, RNA was sequenced from *Nb* leaves transiently expressing AvrXa10 or AvrXa10ΔCRR at 32 and 48 hpi (Figure 2A). A two-fold change in expression and $P \leq 0.05$ were used as cutoff values, and the *Nb* genome (v.0.4.4) was used as a reference (ftp://ftp.solgenomics.net/genomes/Nicotiana_benthamiana). Using this approach, 1425 and 1265 genes were differentially upregulated in the presence of AvrXa10 at 32 and 48 hpi, respectively, compared with AvrXa10 ΔCRR (Figure 2B and Supplemental Dataset 2). We then compared the upregulated genes at the two different time points and identified 804 genes that were induced at 32 and 48 hpi (Figure 2B). The TALgetter computational tool (Grau

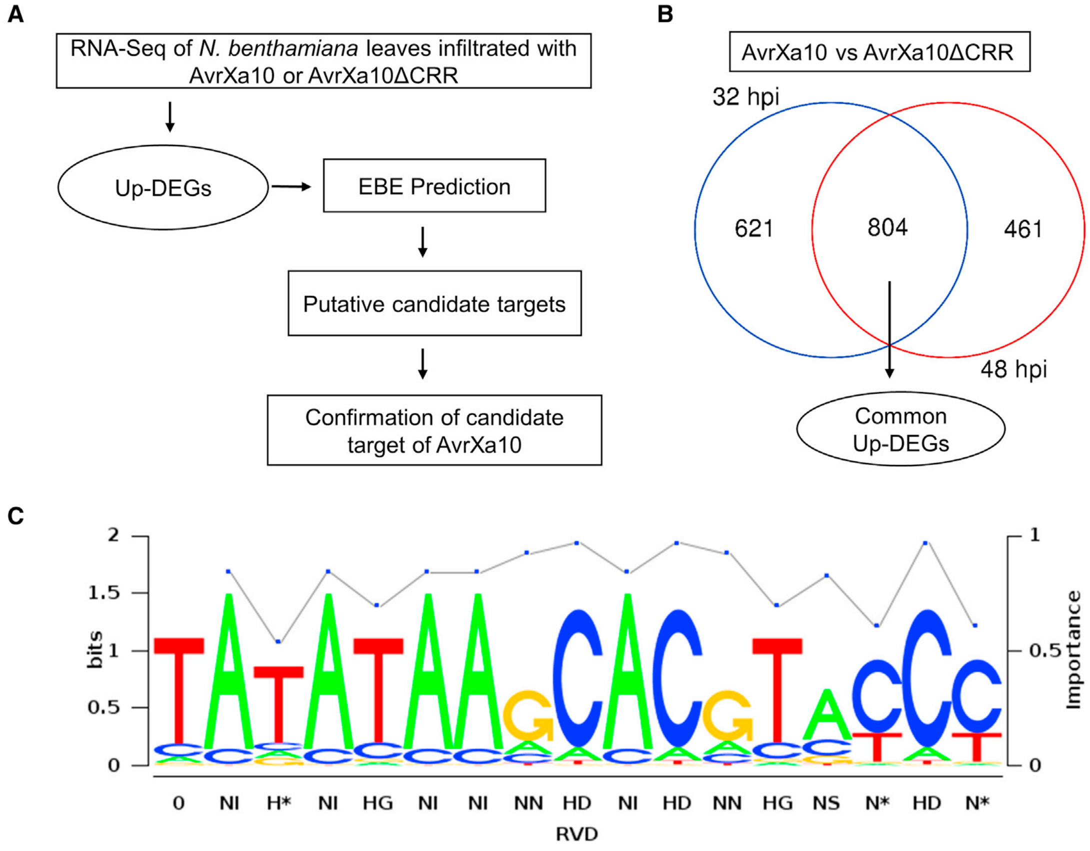

Figure 2. Experimental design for identification of candidate AvrXa10 target genes in *N. benthamiana*.

(A) Schematic flowchart of experimental design. RNA was sequenced from *Nb* leaves transiently expressing AvrXa10 or AvrXa10ΔCRR at 32 and 48 hpi. Differentially regulated genes were identified at the two time points, and upregulated DEGs common to both time points were identified. The TALgetter computational tool was then used to predict AvrXa10 EBEs in the promoter regions of the upregulated genes.

(B) Venn diagram of DEGs upregulated by AvrXa10 but not by AvrXa10ΔCRR at 32 and 48 hpi.

(C) The predicted theoretical EBE of AvrXa10 RVDs. The AvrXa10 RVDs and their associated nucleotides are shown. The logo was produced using TALgetter (Galaxy v.1.1 http://galaxy.informatik.uni-halle.de/).

et al., 2013) was then used to predict AvrXa10 EBEs in the promoter regions of these upregulated genes. A script for promoter extraction (Supplemental Dataset 1) and the *Nb* genome were used to identify promoter regions ~2.0 kb upstream of the ATG site in the upregulated genes. EBEs potentially recognized by AvrXa10 were identified in the promoter regions of upregulated differentially expressed genes (DEGs) using the default parameters of TALgetter. Among the 804 DEGs, 16 had promoter regions that contained predicted EBEs for AvrXa10 (Supplemental Dataset 2). These 16 genes were ranked based on their EBE prediction scores, and the proximity of the EBE to the ATG and TATA box was noted; 6 genes were deleted as candidate targets because the AvrXa10 EBE was located too far away from the ATG and TATA box. The 10 genes listed in Supplemental Table 1 were designated as the putative targets of AvrXa10.

Analysis of candidate AvrXa10 target genes in *N. benthamiana*

Our working hypothesis was that one of the genes in Supplemental Table 1 functions as an *R* gene targeted by AvrXa10. To identify the direct target of AvrXa10, expression of the candidate genes listed in Supplemental Table 1 was monitored in *Nb* leaves transiently expressing AvrXa10 or AvrX10ΔCRR (control). We found that genes *29135g*, *36259g*, *20731g*, *44252g*, *45656g*, and *3269g* were significantly induced by AvrXa10 compared with AvrXa10ΔCRR at both 32 and 48 hpi (Supplemental Figure 3), which agreed with the RNA-sequencing data. To investigate whether the presence of AvrXa10 induces the expression of the candidate genes, we cloned ~1 kb promoter regions of the six highly induced genes upstream of a *gusA* reporter gene in pCAMBIA1381. The six promoter::GUS fusions (p29135g::GUS, p32659g::GUS, p20731g::GUS, p44252g::GUS, p45656g::GUS, and p3692g::GUS) were individually co-expressed in *Nb* with pHB-AvrXa10 by *Agrobacterium*-mediated transformation (Figure 3A and Supplemental Table 2). As a control, the *Os8N3* promoter region, which has an EBE bound by the TALE PthXo1 (Yang et al., 2006), was inserted upstream of *gusA* in pCAMBIA1381 to form the pOs8N3::GUS construct; this was co-expressed in *Nb* with pHB-pthXo1 (Figure 3A and Supplemental Table 2). There was a significant level of GUS expression when pHB-AvrXa10 was co-expressed with p29135g::GUS, p32659g::GUS, p20731g::GUS, p44252g::GUS, p45656g::GUS, or p3692g::GUS, but not with pOs8N3::GUS (Figure 3B). Furthermore, *GUS* expression was higher with the *p29135g*,

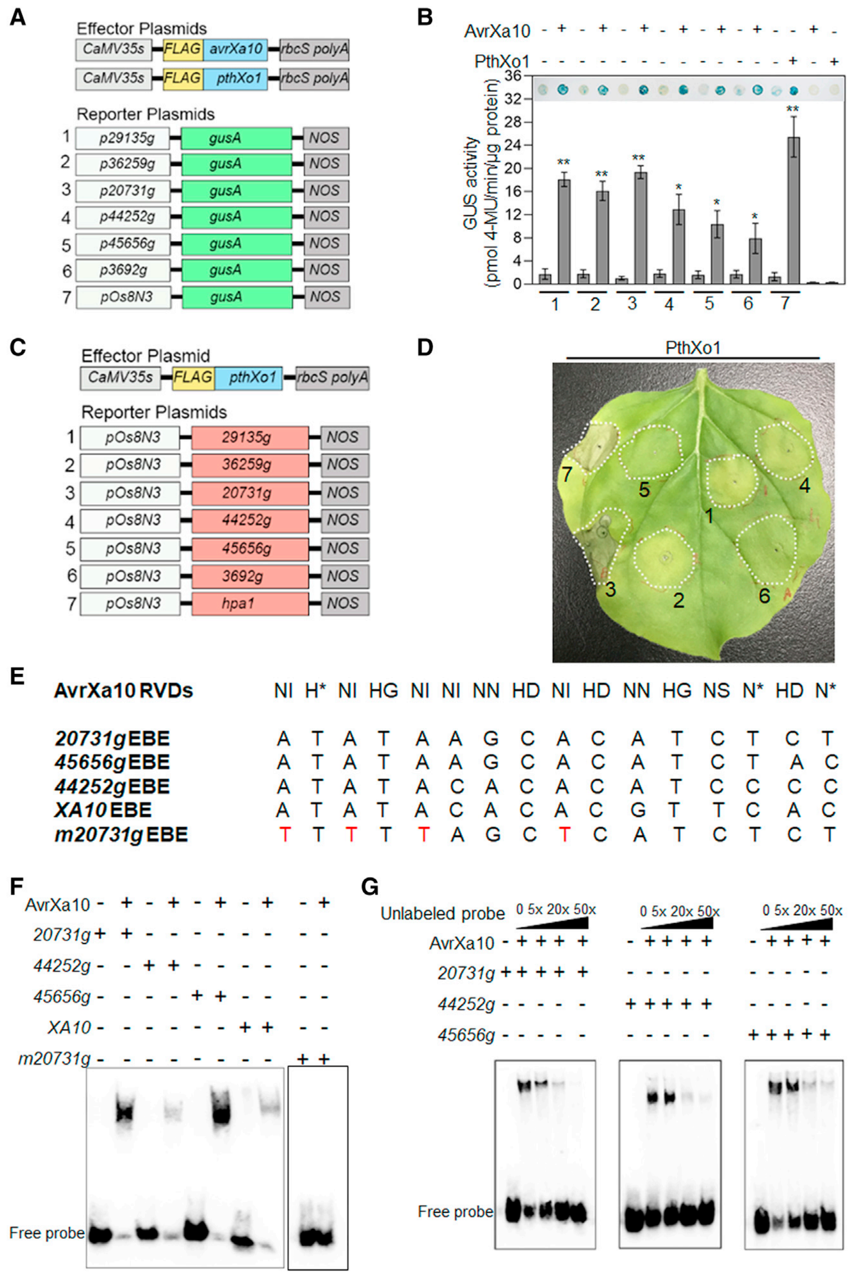

Figure 3. AvrXa10 activates the expression of potential target genes by binding EBEs in promoter regions.

(A) Functional maps of effector and reporter plasmid constructs. The effector constructs contained FLAG-tag fused AvrXa10 or PthXo1 in vector pHB under the control of the CaMV 35S promoter. Reporter constructs contained *gusA* reporter cassettes that were driven by the candidate gene promoters (~1 kb in length); these were cloned in pCAMBIA1381. Abbreviations: rbcS, ribulose-1,5-bisphosphate carboxylase, small subunit; polyA, polyadenylation site; NOS, NOS terminator site.

(B) *Nb* promoters from six genes direct the AvrXa10-dependent, transient expression of GUS in *N. benthamiana*. The photographs of qualitative GUS assays are shown above the bars. The TALE PthXo1 and the *Os8N3* promoter were used as a control. Samples were collected at 36 hpi, and GUS activity was calculated. Error bars indicate means ± SD ($n = 3$), and asterisks indicate significant differences (*$P \leq 0.05$; **$P \leq 0.01$). The experiment was performed at least three times with similar results.

(C) Schematic map of effector and reporter constructs used to identify target genes that promote an HR in *Nb*. The effector constructs contained FLAG-tag fused *pthXo1* under the control of the CaMV 35S promoter. The reporter constructs contained the coding sequences of candidate target genes fused with the *Os8N3* promoter, and the *Os8N3-hpa1* construct served as a positive control.

(D) HR assay in *N. benthamiana*. *Agrobacterium* strains containing the effector construct (pHB-pthXo1) and one of the six reporter constructs were infiltrated into fully expanded *Nb* leaves, which were evaluated for the HR at 4–7 dpi. Representative results were chosen from five independent experiments. Legend: 1, PthXo1 + pOs8N3::29135g; 2, PthXo1 + pOs8N3::36259g; 3, PthXo1 + pOs8N3::20731g; 4, PthXo1 + pOs8N3::44252g; 5, PthXo1 + pOs8N3::45656g; 6, PthXo1 + pOs8N3::3692g; and 7, PthXo1 + pOs8N3::Hpa1.

(E) Oligonucleotide sequences of the EBE probes used in electrophoretic mobility-shift assays. *Xa10* EBE was used as a positive control, and the mutated *20731g* EBE was used as a negative control. The AvrXa10 RVDs corresponding to the EBE sequences are shown above.

(F) Electromobility shift assays using biotin-labeled putative EBE fragments derived from the promoter regions of *20731g*, *44252g*, and *45656g.* The *XA10* EBE probe was used as a positive control.

(G) Binding specificity of AvrXa10 to the target EBEs. Competition of biotinylated probes with unlabeled probes that were used at increasing concentrations (0, 5, 20, and 50×). The experiments were repeated three times.

p32659g, and *p20731g* promoters ($P \leq 0.01$) compared with those of *p44252g*, *p45656g*, and *p3692g* ($P \leq 0.05$); this may be caused by weaker binding between effector and promoter, or it may reflect reduced activation ability. These results indicate that AvrXa10 can induce the expression of these six genes in *Nb* by potentially targeting an EBE site in their promoter regions.

A novel *in vivo* reporter system was developed to determine which of the six candidate target genes could trigger an HR once activated *in planta*. In this experiment, *gusA* was replaced with the coding sequence (CDS) of a target gene, and expression was driven by the *Os8N3* promoter (Figure 3C and Supplemental Table 2); a *pOs8N3-hpa1* construct served as a positive control. The reporter constructs were transiently co-expressed in *Nb* with the effector *pthXo1* (Figure 3C and 3D). In this screening assay, an HR would develop if a target gene were expressed under the *Os8N3* promoter, which depends on PthXo1 binding in this assay (Yang et al., 2006). The construct *pOs8N3-hpa1* served as a positive HR-inducing control; *hpa1* encodes a harpin protein that elicits an HR in *Nb* (Zou et al., 2006). pOs8N3-EV was co-infiltrated with pHB-pthXo1 as a negative control (Supplemental Figure 4). When PthXo1 was co-expressed with *pOs8N3::20731g*, HR-like cell

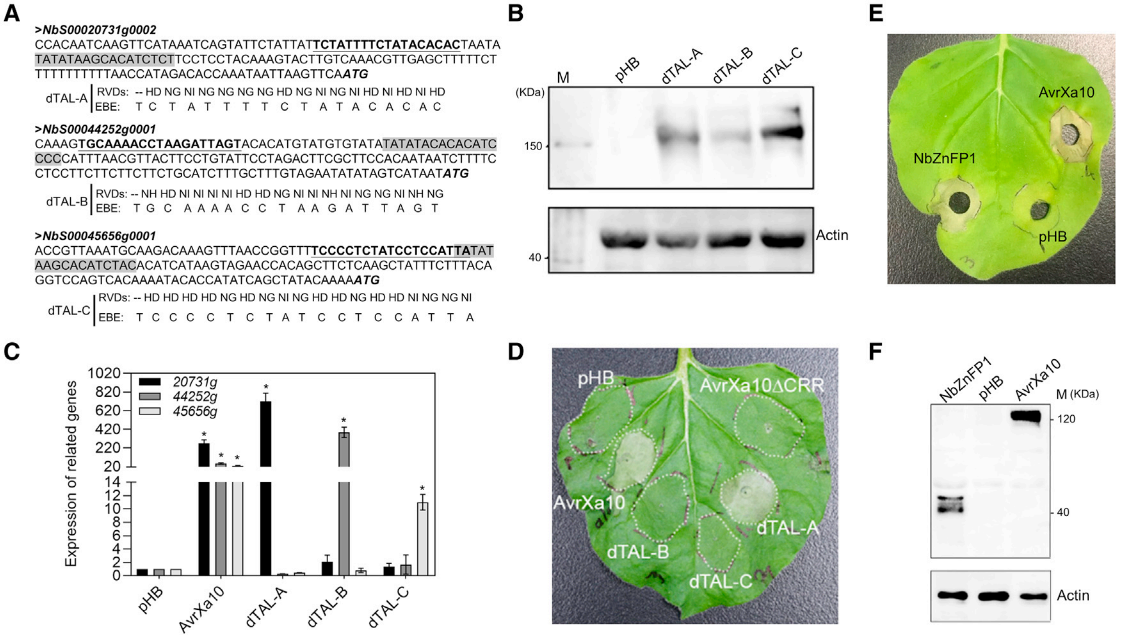

Figure 4. Overexpression of *20731g* (*NbZnFP1*) results in the HR in *N. benthamiana*.

(A) Designer TALE assays. Promoter sequences of *Nb20731g*, *Nb44252g*, and *Nb45656g*; bold, underscored bases indicate sites for dTALE insertion. The gray highlighted region shows the putative AvrXa10 EBE, and the repeat variable di-residues of dTAL-A, dTAL-B, and dTAL-C and target EBEs are shown.

(B) Detection of dTAL-A, dTAL-B, and dTAL-C by western blot analysis. Fully expanded *Nb* leaves were infiltrated with pHB-TAL constructs, and FLAG-tagged dTALs were detected at 48 hpi. The pHB vector and actin protein were used as negative and loading controls, respectively.

(C) Expression analysis of *20731g*, *44252g*, and *45656g* in *Nb* leaves infiltrated with *Agrobacterium* carrying the pHB constructs dTAL-A, dTAL-B, and dTAL-C; pHB and pHB-AvrXa10 served as negative and positive controls, respectively. Four-week-old *Nb* leaves were infiltrated and collected at 36 hpi for qRT-PCR. Error bars indicate means ± SD (n = 3), and asterisks indicate significant differences (*$P \leq 0.01$). The results shown are representative of three independent replicates.

(D) dTAL-A induces the HR in *N. benthamiana*. Constructs were transiently expressed in fully expanded *Nb* leaves via infiltration with *Agrobacterium* (OD_{600} 0.8). Constructs included pHB-dTAL-A, pHB-dTAL-B, pHB-dTAL-C, pHB-AvrXa10 (positive control), pHB-AvrXa10ΔCRR, and pHB. *Nb* leaves were photographed at 3 dpi. All experiments were repeated three times with similar results.

(E) Transient overexpression of *NbZnFP1* in *Nb* leaves. The *Agrobacterium* strains containing pHB-AvrXa10, pHB-NbZnFP1, or empty pHB vector were infiltrated into *Nb* leaves with a needleless syringe. The phenotype was photographed at 3 dpi.

(F) For the western blotting assay, the center region of infiltrated leaf samples was collected at 2 dpi.

death was apparent starting at 4 dpi (Figure 3D, infiltration site 3). The co-expression of PthXo1 with *pOs8N3*::*44252* and *pOs8N3*::*45656* induced a partial HR starting at 7 dpi, but the HR was not produced consistently (Supplemental Figure 4). The other three candidate genes, *29135g*, *32659g*, and *p3692g*, did not produce an HR in this assay. Based on these results, *20731g*, *44252g*, and *45656g* were subjected to further analyses.

The potential binding of AvrXa10 to the promoter region of the three candidate target genes *20731g*, *44252g*, and *45656g* was investigated in electrophoretic mobility-shift assays (EMSAs); the Xa10 EBE (Tian et al., 2014) was used as a positive AvrXa10-interacting control. AvrXa10 was purified from pET30a-AvrXa10 as a C-terminal histidine fusion protein and incubated with the biotin-labeled putative EBE fragments present in the promoter regions of *20731g*, *44252g*, and *45656g* (Figure 3E and 3F). EMSA indicated that all three candidate genes contained potential EBE sites that were recognized and bound by AvrXa10 (Figure 3F). AvrXa10 was unable to bind a mutated EBE fragment of *20731g* (Figure 3F), confirming its binding specificity to the targeted EBE. The specificity of AvrXa10 binding was further confirmed by performing competition assays with labeled and unlabeled EBEs (Figure 3G). The intensity of the AvrXa10-bound putative EBE fragments from *20731g*, *44252g*, and *45656g* was reduced by increasing the concentration of unlabeled EBE probes (Figure 3G). Collectively, these results suggest that AvrXa10 binds to all three promoters via effector binding sites.

Overexpression of *NbZnFP1* elicits HR in *N. benthamiana*

Designer TALEs (dTALEs) designated dTAL-A, dTAL-B, and dTAL-C were generated to target other sites upstream of the AvrXa10-EBEs in the promoter regions of *20731g*, *44252g*, and *45656g*, respectively (Supplemental Figure 5A and Figure 4A), and were used to identify genes responsible for AvrXa10-triggered HR in *Nb*. The dTALEs were cloned into a binary pHB vector with an N-terminal FLAG-tag epitope and were transiently expressed in *Nb* via *Agrobacterium*; western blot analysis confirmed that the dTALEs were expressed in *Nb* leaves (Figure 4B and Supplemental Methods 3). qRT-PCR assays showed that *20731g*, *44252g*, and *45656g* were specifically and significantly induced by dTAL-A, dTAL-B, and dTAL-C,

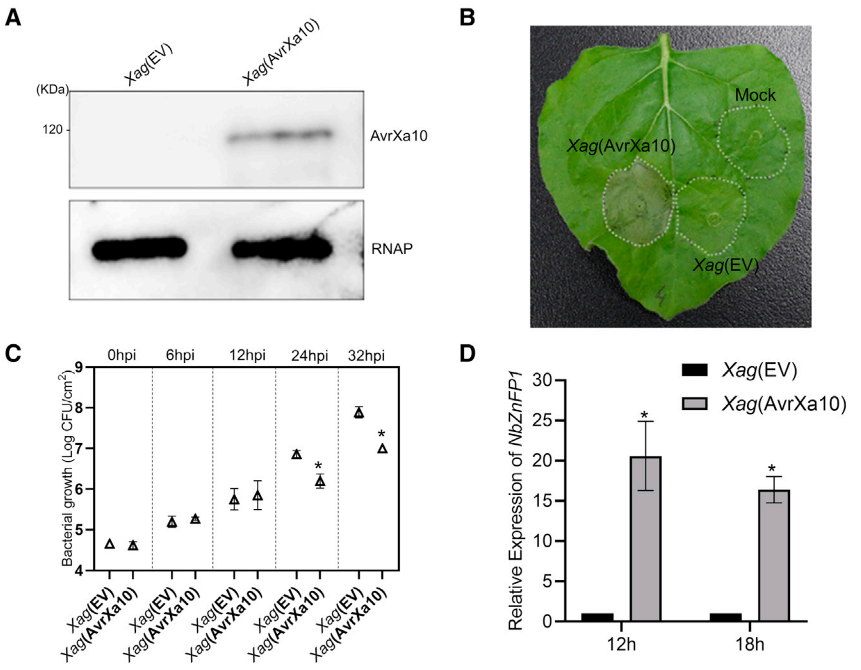

Figure 5. AvrXa10 causes cell death when delivered by *X. axonopodis* pv. *glycines* (*Xag*) in *N. benthamiana*.

(A) Detection of AvrXa10 production in *Xag* by western blotting using an anti-FLAG primary antibody (see methods). RNA polymerase subunit alpha (RNAP) from *E. coli* was used as a loading control.

(B) HR phenotype at 24 hpi. The *Xag* strains containing AvrXa10 or empty vector (EV) were inoculated into *Nb* leaves. The simple buffer $MgCl_2$ was used as a mock control. The experiment was repeated three times with similar results.

(C) Quantification of bacterial growth at different time points. The *Xag* strains containing AvrXa10 or empty vector (EV) were inoculated into *Nb* leaves. The infiltrated leaf samples were collected with a 1-cm diameter cork borer at different time points (0, 12, 24, and 32 hpi). Three leaf discs from three different plants were used as a single replicate, and three replicates were used for this experiment. Error bars indicate means ± SD (n = 3), and asterisks indicate significant differences ($^*P \leq 0.01$).

(D) qRT-PCR analysis of *NbZnFP1* in *Nb* leaves upon inoculation with derivatives of the *Xag* strain. Fully expanded leaves were infiltrated with *Xag* strains carrying AvrXa10 or pHM1 (EV), then collected at 24 hpi for RNA isolation. *NbEF1α* was used as an internal control. Error bars represent means ± SD (n = 3), and asterisks indicate significant differences ($^*P \leq 0.05$). The results shown are representative of three independent replicates.

respectively (Figure 4C). Functional analysis indicated that transient expression of dTAL-A targeting *20731g* elicited an HR in *Nb* at 3 dpi; HR-mediated cell death was not observed with dTAL-B or dTAL-C targeting *44252g* or *45656g*, respectively (Figure 4D). Collectively, these data demonstrate that the activation of *Nb* target *20731g*, which encodes a C2H2-type ZnFP 1 (hereafter NbZnFP1; Supplemental Table 1), elicits the HR in *Nb*.

To test whether transient expression of *NbZnFP1* in *Nb* leaves causes HR, the full-length CDS of *NbZNFP1* was amplified from *Nb* cDNA using the primers NbZnFP1-F/Nb-ZnFP1-R listed in Supplemental Table 3 and was cloned into the pHB vector, which is driven by the CaMV35S promoter. The plasmid pHB-NbZnFP1 was transformed into *Agrobacterium* strain EHA105, and the strain was infiltrated into *Nb* leaves. The *Agrobacterium* strains containing pHB-AvrXa10 and empty pHB vector were used as controls. The results showed that transient overexpression of *NbZnFP1* in *Nb* leaves can also cause HR (Figure 4E). Western blot results indicated that the NbZnFP1 protein was detected in high amounts in the leaf sample showing visible HR (Figure 4F). Overall, these results confirmed that overexpression of NbZnFP1, either by activation of *NbZnFP1* using dTALE or by transient expression, can cause HR in *Nb* leaves. Thus, NbZnFP1 is the biologically relevant target of AvrXa10 and functions as an *R* gene in *Nb*.

AvrXa10 activates *NbZnFP1* and causes cell death when delivered into plant cells by *Xanthomonas*

Expression of AvrXa10 in *Nb* leaves via *Agrobacterium* showed that AvrXa10 triggers HR by activating *NbZnFP1*. We further evaluated this phenomenon by delivering AvrXa10 into plant cells using the *Xanthomonas axonopodis* pv. *glycines* (*Xag*) strain ATCC43911, which possesses a functional T3SS but does not cause HR in *Nb* (Liu et al., 2016). The pHZW-AvrXa10 construct and the pHM1 empty vector (EV) were transformed into *Xag* ATCC43911 (Supplemental Methods 4). The expression of AvrXa10 in *Xag* ATCC43911 was confirmed by western blotting (Figure 5A). The leaves of *Nb* were infiltrated with *Xag* strain ATCC43911 carrying AvrXa10 or an EV and mock control. AvrXa10 caused HR at 24 hpi in *Nb* leaves when delivered by *Xag* (Figure 5B) and also significantly reduced bacterial growth at 24 and 32 hpi (Figure 5C). The qRT-PCR result showed that AvrXa10 activated *NbZnFP1* in *Nb* leaves (Figure 5D). Overall, these data further confirmed that AvrXa10 activates *NbZnFP1* to trigger immunity in *Nb* when delivered by *Xanthomonas* (*Xag*).

On-spot silencing of *NbZnFP1* inhibits AvrXa10-mediated HR

We used the on-spot silencing approach (Johansen and Carrington, 2001) to further confirm that *NbZnFP1* activation by AvrXa10 mediates the HR in *Nb*. A partial CDS (285 bp) of *NbZnFP1* was amplified from *Nb* and cloned into pYL156, resulting in pYL156-NbZnFP1 (Supplemental Table 2). *Nb* leaves were infiltrated with *Agrobacterium* suspensions containing the helper plasmid pTRV-RNA1, the virus-induced gene silencing (VIGS) construct pYL156-NbZnFP1 or pYL156 (EV), and pHB-AvrXa10 (see methods). Leaves inoculated with AvrXa10 + VIGS-NbZnFP1 showed no obvious HR in comparison to leaves inoculated with AvrXa10 + VIGS-EV at 3 dpi (Figure 6A), suggesting that *NbZnFP1* expression was silenced in the VIGS-NbZnFP1 treatment. RT-PCR analysis of inoculated leaves in Figure 6A indicated that the expression of *NbZnFP1* was reduced in the VIGS-NbZnFP1 treatment compared with the VIGS-EV-inoculated leaves (Figure 6B). These results suggested that

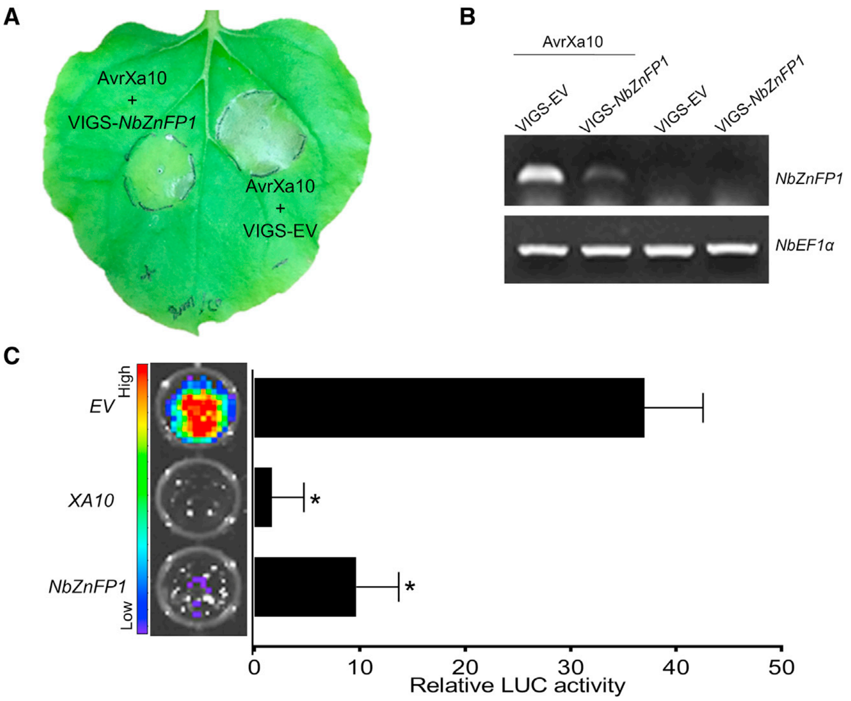

Figure 6. *NbZnFP1* is the biologically relevant target of AvrXa10 in *N. benthamiana*.
(A) On-spot VIGS-mediated silencing of NbZnFP1 partially inhibits AvrXa10-induced HR. *Nb* leaves were co-infiltrated with *Agrobacterium* carrying pHB-AvrXa10 + pTRV-RNA1 + pYL156-NbZnFP1 or pHB-AvrXa10 + pTRV-RNA1 + pYL156 (EV); these two combinations are labeled AvrXa10 + VIGS-NbZnFP1 and AvrXa10 + VIGS-EV, respectively. *Nb* leaves were photographed at 3 dpi.
(B) RT-PCR analysis of *NbZnFP1* expression in *Nb* after on-spot VIGS. Infiltrated leaves were collected at 3 dpi and analyzed for *NbZnFP1* expression by RT-PCR; *NbEF1α* served as a reference gene.
(C) Transient expression of *NbZnFP1* induces cell death in rice protoplasts. Constructs pRTVcHA-NbZnFP1 (NbZnFP1) and pRTVcHA (EV, empty vector) were co-expressed with the LUC reporter construct pRTVcVC-LUC in rice protoplasts. Co-transfection of pRTVcHA-Xa10 (XA10) with the LUC construct was used as a positive, cell-death-inducing control. LUC activity was measured after 24 h of transfection using the Promega LUC assay system. The images on the left side of the graph show microtiter plates containing protoplasts expressing the constructs. The image of LUC fluorescence was taken with a CCD imaging system (IVIS Spectrum, PerkinElmer, USA). The graph on the right shows the relative LUC activity measured with a luminometer (Tecan, M200). Cell death in protoplasts was monitored by the reduction in luciferase activity. Error bars represent means ± SD ($n = 3$), and asterisks indicate significant differences (*$P \leq 0.01$). The results are representative of three replicates.

on-spot silencing of *NbZnFP1* impaired the HR caused by AvrXa10, providing further evidence that *NbZnFP1* is the *R* gene activated by AvrXa10 for HR induction in *Nb*.

Transient overexpression of *NbZnFP1* causes cell death in rice protoplasts

To investigate the potential use of *NbZnFP1* in bacterial leaf blight resistance, we attempted to generate rice transgenic lines expressing *NbZnFP1* with its native promoter, which contains the AvrXa10 EBE; however, stable transgenic plantlets were not obtained. We therefore used a transient expression system to investigate the functionality of *NbZnFP1* in rice protoplasts. *NbZnFP1* was co-expressed with a luciferase reporter construct in rice protoplasts, and cell death was evaluated by monitoring changes in LUC activity (Figure 6C). Transient co-expression of the EV (pRTVcHA) or *Xa10* (pRTVcHA-Xa10) with the LUC construct served as negative and positive controls, respectively. Transient expression of *NbZnFP1* significantly reduced LUC activity compared with the EV (Figure 6C), indicating that the expression of the candidate target *NbZnFP1* causes cell death in rice protoplasts.

Phylogenetic analysis of *NbZnFP1* and subcellular localization

Phylogenetic analysis revealed that NbZnFP1 is closely related to AtZFP1 (Supplemental Figure 6A). NbZnFP1 and orthologs from different plant species contained a conserved C2H2 domain and an ethylene-responsive transcription factor-associated amphiphilic repressor (EAR) motif (Supplemental Figure 6B). Because some ZnFPs function as transcription factors in the plant nucleus (Koguchi et al., 2017; Zhang et al., 2019; Han et al., 2020), the subcellular localization of NbZnFP1 was investigated. The full-length CDS of *NbZnFP1* was cloned into pYFP and transiently expressed in *Nb* leaves. At 2 dpi, confocal microscopy indicated that NbZnFP1 was localized in the nucleus (Supplemental Figure 6C).

Rice has nine orthologs of NbZnFP1 that contain the conserved C2H2 domain and EAR motif at the C terminus (Supplemental Figure 7A). However, the promoter regions of these nine genes lack EBE sites that might be recognized by AvrXa10. Rice leaves were infiltrated with *Xoo* strain PH (*tal*-free strain, Supplemental Table 2) carrying AvrX10, AvrXa10ΔCRR, or an EV control. There were no consistent differences in gene expression among the nine homologs in response to AvrXa10 or AvrXa10ΔCRR as measured by qRT-PCR (Supplemental Figure 7B), implying that the increased expression of these nine genes may cause cell death in rice. To test this possibility, four rice orthologs of *NbZnFP1* (*Os41110*, *Os26210*, *Os44190*, and *Os13600*) were randomly selected and individually co-expressed with the luciferase reporter construct in rice protoplasts, and cell death was evaluated by monitoring changes in LUC activity (Supplemental Figure 7C). Indeed, the overexpression of these four rice genes, like *NbZnFP1*, significantly reduced LUC activity compared with the EV (Supplemental Figure 7C), indicating that transient overexpression of NbZnFP1 family genes causes cell death in rice protoplasts.

AvrXa10 triggers the HR in tomato by activating an NbZnFP1 ortholog

Tomato, like *Nb*, is a member of the Solanaceae. Interestingly, four orthologs of NbZnFP1 that contained a putative EBE site for AvrXa10 in their promoter regions were identified in tomato (Figure 7A). When 4-week-old tomato (cv. Ailsa Craig) leaves

A

Gene ID	AvrXa10-EBE sequence	EBE score	bp to ATG
Solyc03g117070.1.1	TATACTATAAAATCCCC	18.7	275
Solyc07g006880.1.1	TATACACACAAATACAC	14.27	177
Solyc00g014800.1.1	TACATACATACATTCCC	17.87	31
Solyc10g078990.1.1	TATATACACACATATAT	10.97	4
NbZnFP1	TATATAAGCACATCTCT	7.52	77

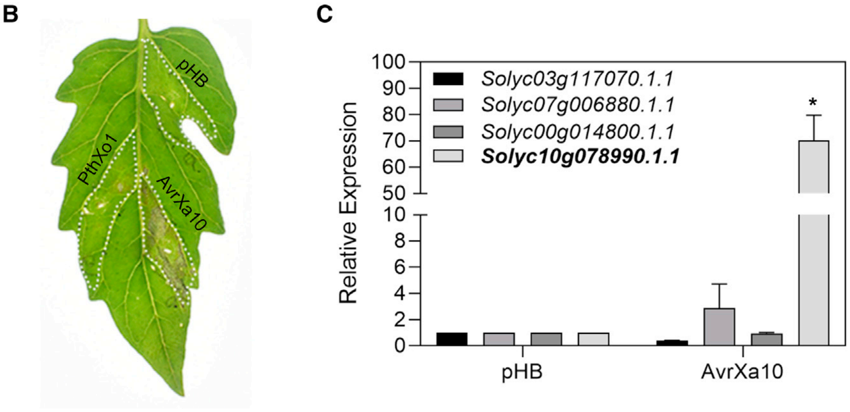

Figure 7. AvrXa10 triggers HR in tomato, probably by activating a zinc finger-like protein gene (*ZnFP*)
(A) Putative AvrXa10 EBEs in the promoters of *ZnFP* homologs in tomato. The AvrXa10-EBE in the promoter of *NbZnFP* is also shown.
(B) HR phenotypes at 4 dpi.
(C) qRT-PCR analysis of the four *ZnFP* homologs. Leaves of 4-week-old tomato cv. Ailsa Craig were infiltrated with *Agrobacterium* strains containing pHB-AvrXa10, pHB-AvrXa10ΔCRR, pHB-AvrXa10ΔAD, or pHB (empty vector). Samples were collected at 32 hpi for RNA isolation and qRT-PCR analysis. The *SolyEF1α* gene was used to normalize the data. Error bars represent means ± SD (n = 3), and asterisks indicate significant differences (*$P \leq 0.01$). The results shown are representative of three independent replicates.

were infiltrated with *Agrobacterium* strains containing pHB-AvrXa10, pHB-PthXo1, or pHB (EV), a prominent HR induced by AvrXa10 was observed (Figure 7B). We then investigated whether AvrXa10 could activate any of the four *NbZnFP1* homologs in tomato (Figure 7B). qRT-PCR analysis indicated that one homolog, *Solyc10g078990.1.1*, was significantly induced by AvrXa10 at 32 hpi (Figure 7C). This suggested that AvrXa10 specifically activates the expression of *Solyc10g078990.1.1* for HR cell death, similar to that in *Nb*.

DISCUSSION

Xoo elicits a T3SS-dependent HR in *Nb* (Li et al., 2012). Known *Xoo* factors that elicit HR in *Nb* include Hpa1, Ssb, XopL, XopP1, and XopY (Li et al., 2013; Ma et al., 2020; Zou et al., 2006). In this study, the role of 17 *Xoo* TALE proteins in mediating the HR in *Nb* was systematically evaluated, and only AvrXa10 elicited a stable HR when transiently expressed in *Nb* (Supplemental Figure 1). AvrXa10 elicited a strong HR in *Nb* at relatively low OD values, suggesting that the interaction was specific. AvrXa10 has previously been used in *Nb* with promoter–GUS reporter assays, and no cell death was reported (Boch et al., 2009; Tian et al., 2014), perhaps because of timing issues. In GUS reporter assays, samples are typically collected at 36 hpi, and the AvrXa10-triggered HR in *Nb* leaves starts at 3 dpi. This can also be observed in our GUS results, in which AvrXa10 did not show visible HR when the samples were collected early (Figure 3B). To meet the criteria for NHR, the target *R* gene must be dependent on a cognate TALE for transcription (Zhang et al., 2015). Our results show that the CRR and AD of AvrXa10 are specifically required to induce the HR in *Nb*, suggesting that the target gene is dependent on AvrXa10 for activation (Figure 1). AvrXa10 was previously shown to activate the expression of the *R* gene *Xa10* in rice, and this activation resulted in the HR (Tian et al., 2014). However, *Xa10* homologs are absent in *Nb*, indicating that AvrXa10 activates a different target gene for HR induction in *Nb*.

Transcriptome analysis and EBE prediction are valid approaches for the identification of TALE-activated target genes. For example, the *R* gene *Bs4c* was identified in pepper by sequencing host RNA in the presence or absence of AvrBs4 (Strauß et al., 2012). Similarly, *GhSWEET10* in cotton and *TaNCED-5BS* in wheat were identified based on transcriptome profiling and EBE prediction of TALE-binding sites (Cox et al., 2017; Peng et al., 2019). In the current study, RNA sequencing and EBE prediction were used to identify 16 putative AvrXa10 targets in *Nb* (Figure 2). Analysis by qRT-PCR, EMSA, and a novel reporter assay indicated that *20731g*, *44252g*, and *45656g* were probable AvrXa10 targets (Supplemental Figures 3 and 4 and Figure 3). The *44252g* gene encodes a GDSL-esterase/lipase; interestingly, a similar enzyme was involved in the NHR of *Arabidopsis thaliana* (Langenbach et al., 2016). The *45656g* gene encodes an ethylene-responsive transcription factor, which is relevant because the overexpression of a similar tobacco gene, *NtERF3*, triggered an HR in tobacco (Ogata et al., 2012). The target *20731g* encodes a C2H2-type ZnFP, and ZnFPs are known to function in the plant stress response (Kim et al., 2004; Shi et al., 2014; Yu et al., 2016; Han et al., 2020). It is noteworthy that many R gene products and defense proteins contain zinc finger domains (Gupta et al., 2012).

Although qRT-PCR, *in vivo* reporter assays, and EMSA indicated that *20731g*, *44252g*, and *45656g* were potential targets of AvrXa10, it was unclear which gene was the biologically relevant target of AvrXa10 or whether the HR caused by AvrXa10 was due to the collective activity of all three genes. This question was addressed by utilizing dTALEs, which have been used to identify potential TALE gene targets (Cernadas et al., 2014; Cohn et al., 2014; Cox et al., 2017; Peng et al., 2019). We constructed dTALEs that specifically induced each candidate gene by targeting a unique sequence in its promoter region. Only dTAL-A targeting *20731g* triggered the HR in *Nb* when transiently expressed (Figure 4). qRT-PCR showed that dTAL-A specifically induced the expression of target *20731g* and not the expression

of the other two candidate genes. dTAL-B and dTAL-C specifically activated *44252g* and *45656g*, but did not cause the HR in *Nb.* On-spot VIGS was used to further validate that *20731g* is targeted by AvrXa10 in *Nb*. The inoculation site where *20731g* was silenced showed a reduced HR in response to AvrXa10, thus confirming that *20731g* (*NbZnFP1*) was the biologically significant target of AvrXa10 in *Nb* (Figure 6). It is important to mention that transient expression of *NbZnFP1* elicited the HR in *Nb* leaves when it was expressed in very high amounts (Figure 4E and 4F). Also, the silencing of *NbZnFP1* did not fully reduce the transcript level, but the HR was no longer completely visible (Figure 6). These results suggest that the AvrXa10-*NbZnFP1* mediated HR is somehow dose dependent.

R genes for race-specific resistance to *Xanthomonas* TALEs have been cloned from rice (*Xa10*, *Xa23*, *Xa27*, and *Xa7*) and solanaceous plants (*Bs3* and *Bs4C-R*) (Xue et al., 2020; Chen et al., 2021; Luo et al., 2021). These *R* genes are not homologous to classical *NLR* genes and are subdivided into two groups (Zhang et al., 2015). Group 1 probably functions in plant development or physiology and includes the *Bs3*-encoded flavin monooxygenases (Römer et al., 2007). Group 2 consists of transmembrane proteins that possess multiple hydrophobic domains (Zhang et al., 2015). This study shows that nonhost plants may encode a third type of resistance protein, namely the C2H2-type ZnFPs.

Phylogenetic analysis of ZnFPs from different plant species indicated that NbZnFP1 is closely related to ZnFPs in *Arabidopsis*, tomato (*Solanum lycopersicum*), and eggplant (*Solanum melongena*) (Supplemental Figure 6). Although homologs of *NbZnFP1* were identified in rice, their promoter regions did not encode the EBE recognized by AvrXa10 and were not induced by AvrXa10 in rice (Supplemental Figure 7). The potential use of *NbZnFP1* to confer *Xoo* resistance was assessed by transforming rice with *NbZnFP1* under the control of its native promoter; however, we were unable to generate stable transgenic plantlets. This may have been caused by lethal effects of *NbZnFP1* when expressed from its endogenous promoter or by other factors outside the scope of the present study.

C2H2-type ZnFPs are transcription factors that contain one or more potential DNA-binding regions comprising 25–30 amino acids (C-X_{2-4}-C-X_3-P-X_5-L-X_2-H-X_3-H) (Han et al., 2020). In addition to roles in transcriptional activation, some ZnFPs also possess an EAR motif that can function as a transcriptional repressor (Han et al., 2020). In this study, the NbZnFP1 deduced protein sequence revealed a single C2H2 domain and an EAR motif at the C terminus. The subcellular localization experiments showed that both NbZnFP1 and AvrXa10 localized to the nucleus (Supplemental Figure 6C), suggesting that NbZnFP1 transcriptionally activates an unknown gene, exhibiting cross talk with other defense-related genes (Supplemental Figure 2). This possibility is worth investigating in the future.

C2H2 ZnFPs have been previously characterized in rice and function in development (Liu et al., 2018; Zhuang et al., 2020) and tolerance to abiotic stress, including drought, salt, and acidic soils (Iuchi et al., 2008; Xu et al., 2008; Sun et al., 2010). A growing number of studies have documented the contribution of C2H2 ZnFPs to both abiotic and biotic stress tolerance (Kim et al., 2004; Shi et al., 2014; Yu et al., 2016; Lawrence and Novak, 2018; Yin et al., 2020; Zhao et al., 2020). Furthermore, the potential use of C2H2 ZnFPs in enhancing pathogen resistance in transgenic plants has been successfully demonstrated using the ZFPs CaPIF1 and CaZFP, which were originally identified in pepper (Kim et al., 2004; Oh et al., 2005; Shi et al., 2014).

Certain *R* genes, like *Xa23*, *Xa27*, and *Xa10*, originated from *O. rufipogon* and *Oryza minuta*, which are wild species of domesticated rice (Gu et al., 2004; Wang et al., 2015; Zhang et al., 2001). Recently, a novel locus was identified in the wild species *Oryza latifolia* that conferred race-specific resistance to *Xoo* PXO339 (Angeles-Shim et al., 2020). A relevant example is the use of *Rxo1* from the nonhost maize as a source of resistance to *Xoc* in rice, which indicates that NHR can be used in different crop species (Zhao et al., 2005). It is possible that TALE proteins may be occasionally trapped in nonhost plants, as in this study, in which AvrXa10 was trapped by the ZnFP gene in *Nb* (Figures 1 and 4) and tomato (Figure 7) to promote HR induction. Transient expression of *NbZnFP1* and its homologs in rice protoplasts caused cell death (Figure 6C and Supplemental Figure 7C), suggesting that these *ZnFP* genes could potentially be used in rice resistance against *Xoo*/*Xoc* infection. The transfer of AvrXa10 into *Xag* strain ATCC43911 caused HR in *Nb* (Figure 5), which suggests that the promoter region of *ZnFP1* could be engineered to confer a broad-spectrum resistance by inserting a major TALE EBE and could be used in a strategy that includes other *R* genes against *Xoo* (Zeng et al., 2015). Thus, it remains possible that *NbZnFP1* could be used in a rice resistance breeding program if the barriers to stable transformation are overcome.

METHODS

Bacterial strains, plasmids, plant materials, and DNA manipulation

The bacterial strains and plasmids used in this study are listed in Supplemental Table 2. *Escherichia coli* strains were grown in Luria-Bertani medium at 37°C (Sambrook and Russell, 2001), and *Agrobacterium* was grown in Luria-Bertani containing rifampicin at 28°C. When needed, antibiotics were added at the following final concentrations: ampicillin, 100 $\mu g\ ml^{-1}$; rifampicin, 75 $\mu g\ ml^{-1}$; and kanamycin, 25 $\mu g\ ml^{-1}$. Protocols for DNA manipulation and plasmid construction are provided in Method S1.

Nb plants were cultivated in a growth chamber at 25°C with a 16 h light/8 h dark photoperiod. Four- to eight-week-old *Nb* plants were used for all experiments. Rice cv. Nipponbare (*Oryza sativa* subsp. *japonica*) was grown at 28°C in a greenhouse located at Shanghai Jiao Tong University with a 12 h light/dark photoperiod.

Detection of cell death and ROS accumulation

Cell death was detected in *Nb* by Evans blue staining using established protocols (Wright et al., 2000). After excess dye was removed, *Nb* leaves were destained with 95% ethanol in a boiling water bath, stored in 65% ethanol, and photographed.

Cell death in leaves was also estimated by measuring ion leakage as described previously (Wright et al., 2000), with minor modifications. Two days after agroinfiltration, five leaf discs (1 cm diameter) were immersed in deionized distilled water (5 ml) and incubated at 25°C with gentle agitation for 4 h. Ion leakage was measured with a DDS-12DW conductivity meter (Bante Instruments). All experiments were repeated three times.

ROS were detected in *Nb* leaves with 3,3′-diaminobenzidine at 32 and 48 hpi as described previously (Bindschedler et al., 2006). The experiment was repeated three times with three independent plants.

RNA sequencing

RNA sequencing was provided by Shanghai Ouyi Biomedical Technology Co. (Shanghai, China). Total RNA was extracted from *Nb* leaves inoculated with AvrXa10 or AvrXa10ΔCRR at 32 and 48 hpi using the mirVana miRNA Isolation Kit (Ambion) as recommended by the manufacturer. RNA integrity was evaluated using the Agilent 2100 Bioanalyzer (Agilent Technologies, Santa Clara, CA, USA), and samples with RNA integrity numbers ≥7 were used for library construction. RNA libraries were constructed using the TruSeq Stranded mRNA Sample Prep Kit (Illumina, San Diego, CA, USA) and sequenced using the Illumina platform (HiSeq 2500 or Illumina HiSeq X Ten); 125 bp/150 bp paired-end reads were generated. The *Nb* reference genome used for alignment was downloaded from ftp://ftp.solgenomics.net/genomes/Nicotiana_benthamiana.

Prediction of the EBE recognized by AvrXa10

The EBE recognized by AvrXa10 was predicted in a subset of AvrXa10-upregulated genes using the TALgetter tool (Grau et al., 2013). The *Nb* promoterome, beginning 2 kb upstream of the translation start site, was extracted from genome v.0.4.4 using the script described in Supplemental Dataset 1.

GUS reporter assays

The GUS reporter system was used to assess the transcriptional activation of selected target genes by AvrXa10. Promoter sequences located ~1 kb upstream of the translational start site in *2913g*, *36259g*, *20731g*, *44252g*, *45656g*, and *3692g* were cloned into the binary GUS reporter construct pCAMBIA1381 using primers listed in Supplemental Table 3. The effector and reporter constructs were then co-expressed (OD_{600} 0.8 for each strain) in 4- to 7-week-old *Nb* leaves via *Agrobacterium*-mediated transformation. In qualitative GUS assays, leaf discs (1 cm diameter) were sampled at 2 dpi, immersed in GUS staining solution for 6–8 h (100 mM phosphate buffer with 10 mM EDTA, 0.5% Triton X-100, 0.5 mM $K_3[Fe(CN)_6]$, 0.5 mM $K_4[Fe(CN)_6]$, and 0.5 mM X-gluc), and washed in 70% ethanol. In quantitative assays, three leaf discs (1 cm) were collected at 2 dpi, and GUS activity was measured using 4-methylumbelliferyl-β-glucuronide. Proteins were quantified using the Bradford method (Antony et al., 2010).

A novel *in vivo* reporter system was also utilized in which the *gusA* CDS was replaced with the CDS of target genes and fused with the *Os8N3* promoter. The susceptibility gene *Os8N3* is targeted by the effector PthXo1 in rice (Yang et al., 2006), and Os8N3 and PthXo1 were shown to interact when delivered *in trans* and co-expressed in *Nb* (Cai et al., 2017). This approach enabled us to test whether selected target genes triggered an HR in *Nb*. The reporter constructs were co-expressed with PthXo1 in *Nb*, and phenotypes were observed at 7 dpi. The experiment was repeated three times with five independent plants for each replicate.

Electrophoretic mobility-shift assays

Selected proteins were purified and used in EMSAs. AvrXa10 with a C-terminal 6×His-tag in the pET30a-AvrXa10 construct (Supplemental Table 2) was expressed in *E. coli* and purified as a histidine fusion protein (His-AvrXa10). Complementary pairs of EBE fragments (~25–30 bp) were annealed and labeled with the Biotin 3′ End DNA Labeling Kit (Thermo Scientific, USA). EMSA was performed as described previously (Cai et al., 2017), and photos were taken with the ChemiScope 6000 series imaging system (Clinx Science Instruments Co., Ltd).

Construction of designer TALEs

TAL Effector-Nucleotide Targeter 2.0 (TALE-NT 2.0) (Doyle et al., 2012) was used to construct dTALEs that targeted 18–19 bp promoter regions in the tobacco genes *Nb20731g* (dTAL-A), *Nb44252g* (dTAL-B), and *Nb45656g* (dTAL-C). A library of four basic repeats encoding RVDs NG, NI, HD, and NN, which correspond to target nucleotides T, A, C, and G, respectively, was used. The repeat regions of artificial dTALEs were assembled using the RVDs corresponding to the targeted nucleotides in the promoter regions of *Nb20731g*, *Nb44252g*, and *Nb45656g*. The CRRs of dTALEs were then synthesized by ViewSolid Biotechnology (Beijing, China) using the TALE construction kit (Catalog no. VK006-02) and then cloned into pUC57. Detailed information on the dTALEs, including RVDs and targeted EBE sequences, is provided in Supplemental Figure 5A. For transient expression in *Nb*, the dTALEs were replaced with AvrXa10 in pHB-AvrXa10 at the *Sph*I site, giving rise to pHB-dTALE (Supplemental Figure 5B).

Agro-mediated VIGS of target gene *Nb20731g*

On-spot VIGS was used to downregulate the expression of the target gene *Nb20731g* (*NbZnFP1*) in *Nb* leaves using the tobacco rattle virus (TRV) bipartite system. A 285-bp fragment of *NbZnFP1* was amplified from *Nb* cDNA using primers *NbZnFP1*(VIGS)-F/R (Supplemental Table 3); the PCR product was then cloned into the TRV RNA2-containing vector pYL156 as an *Xba*I/*Bam*HI fragment, resulting in pYL156-NbZnFP1. For VIGS, *Agrobacterium* strains containing the helper vector pTRV-RNA1 and pYL156 (EV) or pYL156-NbZnFP1 were cultured overnight, collected, washed once with infiltration buffer (10 mM $MgCl_2$, 0.2 mM acetosyringone, and 200 mM MES [pH 5.6]), and then resuspended in infiltration buffer to OD_{600} 1.0. The pTRV-RNA1 culture suspension was mixed with suspensions containing pYL156 or pYL156-20731g at a 1:1 ratio and then combined with an *Agrobacterium* suspension containing pHB-AvrXa10. Cultures were mixed and used to infiltrate fully expanded leaves of 5-week-old *Nb*. The phenotypes were observed at 3 dpi and photographed, and one image was chosen from three independent replicates.

Cell death assays in rice protoplasts

The role of *NbZnFP1* (*Nb20731g*) and its four rice orthologs in mediating cell death in rice was explored using a luciferase (*LUC*) reporter system. Protoplasts were isolated from

10-day-old seedlings of rice cv. Nipponbare as described previously (He et al., 2016). The pRTVcVC-LUC construct, containing *LUC* fused with the C terminus of mVenus (Xu et al., 2021), was used as a source of luciferase (Supplemental Table 2). *NbZnFP1* (*Nb20731g*) and *Xa10* were cloned into pRTVcHA (He et al., 2018) as described in Supplemental Methods 1. Two micrograms of pRTVcHA-NbZnFP1, pRTVcHA-Xa10, or pRTVcHA (EV) was co-transfected with pRTVcVC-LUC (2 μg) into rice protoplasts as described previously (He et al., 2016). After a 24-h incubation period, LUC activity and fluorescence were detected using a luminometer (Tecan M200), CCD imaging system (IVIS Spectrum, PerkinElmer, USA), and the Luciferase Assay System (Promega). Co-expression of pRTVcHA-Xa10 (*Xa10*) or pRTVcHA (EV) with the LUC construct served as positive and negative controls, respectively.

ACCESSION NUMBERS

The data that support the findings of this study are available from the corresponding author upon reasonable request.

SUPPLEMENTAL INFORMATION

Supplemental information can be found online at *Plant Communications Online*.

FUNDING

This work was supported by the National Natural Science Foundation of China (31830072), the National Key Research and Development Program of China (2016YFD0100601), and the National Transgenic Major Program (2016ZX08001-002).

AUTHOR CONTRIBUTIONS

F.H. and G.C. designed the experiments. F.H. performed the experiments. X.X. and W.M. provided technical assistance. S.M.A.S., L.L., B.Z., and L.Z. contributed materials. F.H. and G.C. wrote the manuscript. G.C. supervised the project.

ACKNOWLEDGMENTS

We thank Professor Xin Zhong Cai for providing the VIGS vectors. No conflict of interest is declared.

Received: April 6, 2021
Revised: August 29, 2021
Accepted: October 13, 2021
Published: October 14, 2021

REFERENCES

Angeles-Shim, R.B., Shim, J., Vinarao, R.B., Lapis, R.S., and Singleton, J.J. (2020). A novel locus from the wild allotetraploid rice species *Oryza latifolia* Desv. confers bacterial blight (*Xanthomonas oryzae* pv. *oryzae*) resistance in rice (*O. sativa*). PLoS One **15**:e0229155.

Antony, G., Zhou, J., Huang, S., Li, T., Liu, B., White, F., and Yang, B. (2010). Rice *xa13* recessive resistance to bacterial blight is defeated by induction of the disease susceptibility gene *Os-11N3*. The Plant Cell **22**:3864–3876.

Asai, S., and Yoshioka, H. (2009). Nitric oxide as a partner of reactive oxygen species participates in disease resistance to necrotrophic pathogen *Botrytis cinerea* in *Nicotiana benthamiana*. Mol. Plant-Microbe Interact. **22**:619–629.

Bindschedler, L.V., Dewdney, J., Blee, K.A., Stone, J.M., Asai, T., Plotnikov, J., Denoux, C., Hayes, T., Gerrish, C., Davies, D.R., et al. (2006). Peroxidase-dependent apoplastic oxidative burst in Arabidopsis required for pathogen resistance. Plant J. **47**:851–863.

Boch, J., and Bonas, U. (2010). *Xanthomonas* AvrBs3 family-type III effectors: discovery and function. Annu. Rev. Phytopathol. **48**:419–436.

Boch, J., Scholze, H., Schornack, S., Landgraf, A., Hahn, S., Kay, S., Lahaye, T., Nickstadt, A., and Bonas, U. (2009). Breaking the code of DNA binding specificity of TAL-type III effectors. Science **326**:1509–1512.

Bogdanove, A.J., Schornack, S., and Lahaye, T. (2010). TAL effectors: finding plant genes for disease and defense. Curr. Opin. Plant Biol. **13**:394–401.

Cai, L., Cao, Y., Xu, Z., Ma, W., Zakria, M., Zou, L., Cheng, Z., and Chen, G. (2017). A transcription activator-like effector Tal7 of *Xanthomonas oryzae* pv. *oryzicola* activates rice gene *Os09g29100* to suppress rice immunity. Sci. Rep. **7**:5089.

Cernadas, R.A., Doyle, E.L., Niño-Liu, D.O., Wilkins, K.E., Bancroft, T., Wang, L., Schmidt, C.L., Caldo, R., Yang, B., White, F.F., et al. (2014). Code-assisted discovery of TAL effector targets in bacterial leaf streak of rice reveals contrast with bacterial blight and a novel susceptibility gene. PLoS Path **10**:e1003972.

Chen, X., Liu, P., Mei, L., He, X., Chen, L., Liu, H., Shen, S., Ji, Z., Zheng, X., and Zhang, Y. (2021). *Xa7*, a new executor R gene that confers durable and broad-spectrum resistance to bacteria-blight disease in rice. Plant Commun. 100143.

Cohn, M., Bart, R.S., Shybut, M., Dahlbeck, D., Gomez, M., Morbitzer, R., Hou, B.-H., Frommer, W.B., Lahaye, T., and Staskawicz, B.J. (2014). *Xanthomonas axonopodis* virulence is promoted by a transcription activator-like effector–mediated induction of a SWEET sugar transporter in cassava. Mol. Plant-microbe Interact. **27**:1186–1198.

Cox, K.L., Meng, F., Wilkins, K.E., Li, F., Wang, P., Booher, N.J., Carpenter, S.C., Chen, L.-Q., Zheng, H., and Gao, X. (2017). TAL effector driven induction of a *SWEET* gene confers susceptibility to bacterial blight of cotton. Nat. Commun. **8**:1–14.

Deb, D., Anderson, R.G., How-Yew-Kin, T., Tyler, B.M., and McDowell, J.M. (2018). Conserved RxLR effectors from oomycetes *Hyaloperonospora arabidopsidis* and *Phytophthora sojae* suppress PAMP-and effector-triggered immunity in diverse plants. Mol. Plant-microbe Interact. **31**:374–385.

Dou, D., and Zhou, J.-M. (2012). Phytopathogen effectors subverting host immunity: different foes, similar battleground. Cell Host & Microbe **12**:484–495.

Doyle, E.L., Booher, N.J., Standage, D.S., Voytas, D.F., Brendel, V.P., VanDyk, J.K., and Bogdanove, A.J. (2012). TAL effector-nucleotide targeter (TALE-NT) 2.0: tools for TAL effector design and target prediction. Nucleic Acids Res. **40**:W117–W122.

Grau, J., Wolf, A., Reschke, M., Bonas, U., Posch, S., and Boch, J. (2013). Computational predictions provide insights into the biology of TAL effector target sites. Plos Comp. Biol. **9**:e1002962.

Gu, K., Tian, D., Yang, F., Wu, L., Sreekala, C., Wang, D., Wang, G.-L., and Yin, Z. (2004). High-resolution genetic mapping of *Xa27 (t)*, a new bacterial blight resistance gene in rice, *Oryza sativa* L. Theor. Appl. Genet. **108**:800–807.

Gupta, S.K., Rai, A.K., Kanwar, S.S., and Sharma, T.R. (2012). Comparative analysis of zinc finger proteins involved in plant disease resistance. PLoS One **7**:e42578.

Han, G., Lu, C., Guo, J., Qiao, Z., Sui, N., Qiu, N., and Wang, B. (2020). C2H2 zinc finger proteins: master regulators of abiotic stress responses in plants. Front. Plant Sci. **11**:115.

Haq, F., Xie, S., Huang, K., Shah, S.M.A., Ma, W., Cai, L., Xu, X., Xu, Z., Wang, S., and Zou, L. (2020). Identification of a virulence *tal* gene in the cotton pathogen, *Xanthomonas citri* pv. *malvacearum* strain Xss-V_2–18. BMC Microbiol. **20**:1–13.

He, F., Chen, S., Ning, Y., and Wang, G.L. (2016). Rice (*Oryza sativa*) protoplast isolation and its application for transient expression analysis. Curr. Protoc. Plant Biol. **1**:373–383.

Hu, Y., Zhang, J., Jia, H., Sosso, D., Li, T., Frommer, W.B., Yang, B., White, F.F., Wang, N., and Jones, J.B. (2014). Lateral organ boundaries 1 is a disease susceptibility gene for citrus bacterial canker disease. Proc. Natl. Acad. Sci. USA **111**:E521–E529.

Iuchi, S., Kobayashi, Y., Koyama, H., and Kobayashi, M. (2008). STOP1, a Cys2/His2 type zinc-finger protein, plays critical role in acid soil tolerance in Arabidopsis. Plant Signal. Behav. **3**:128–130.

Johansen, L.K., and Carrington, J.C. (2001). Silencing on the spot. Induction and suppression of RNA silencing in the *Agrobacterium*-mediated transient expression system. Plant Physiol. **126**:930–938.

Jones, J.D., and Dangl, J.L. (2006). The plant immune system. Nature **444**:323–329.

Jones, J.D., Vance, R.E., and Dangl, J.L. (2016). Intracellular innate immune surveillance devices in plants and animals. Science **354**:aaf6395.

Kay, S., Hahn, S., Marois, E., Hause, G., and Bonas, U. (2007). A bacterial effector acts as a plant transcription factor and induces a cell size regulator. Science **318**:648–651.

Kim, S.H., Hong, J.K., Lee, S.C., Sohn, K.H., Jung, H.W., and Hwang, B.K. (2004). CAZFP1, Cys_2/His_2-type zinc-finger transcription factor gene functions as a pathogen-induced early-defense gene in *Capsicum annuum*. Plant Mol. Biol. **55**:883–904.

Koguchi, M., Yamasaki, K., Hirano, T., and Sato, M.H. (2017). Vascular plant one-zinc-finger protein 2 is localized both to the nucleus and stress granules under heat stress in Arabidopsis. Plant Signal. Behav. **12**:e1295907.

Langenbach, C., Schultheiss, H., Rosendahl, M., Tresch, N., Conrath, U., and Goellner, K. (2016). Interspecies gene transfer provides soybean resistance to a fungal pathogen. Plant Biotechnol. J. **14**:699–708.

Lawrence, S.D., and Novak, N.G. (2018). The remarkable plethora of infestation-responsive Q-type C2H2 transcription factors in potato. BMC Res. Notes **11**:1–7.

Li, W., Xu, Y.-P., Zhang, Z.-X., Cao, W.-Y., Li, F., Zhou, X., Chen, G.-Y., and Cai, X.-Z. (2012). Identification of genes required for nonhost resistance to *Xanthomonas oryzae* pv. *oryzae* reveals novel signaling components. PLoS One **7**:e42796.

Li, Y.-R., Ma, W.-X., Che, Y.-Z., Zou, L.-F., Zakria, M., Zou, H.-S., and Chen, G.-Y. (2013). A highly-conserved single-stranded DNA-binding protein in *Xanthomonas* functions as a harpin-like protein to trigger plant immunity. PLoS One **8**:e56240.

Li, Z., Zou, L., Ye, G., Xiong, L., Ji, Z., Zakria, M., Hong, N., Wang, G., and Chen, G. (2014). A potential disease susceptibility gene *CsLOB* of citrus is targeted by a major virulence effector PthA of *Xanthomonas citri* subsp. *Citri*. Mol. Plant **7**:912–915.

Liu, L., Ma, W.-X., Zou, L.-F., Chen, X.-B., and Chen, G.-Y. (2016). Comparative analysis of type-III secretion system and T3SS-secreted effectors among seven quarantine *Xanthomonas* pathovars. Acta Phytopathol. Sin. **46**:37–46.

Liu, X., Li, D., Zhang, D., Yin, D., Zhao, Y., Ji, C., Zhao, X., Li, X., He, Q., and Chen, R. (2018). A novel antisense long noncoding RNA, TWISTED LEAF, maintains leaf blade flattening by regulating its associated sense R2R3-MYB gene in rice. New Phytol. **218**:774–788.

Luo, D., Huguet-Tapia, J.C., Raborn, R.T., White, F.F., Brendel, V.P., and Yang, B. (2021). The *Xa7* resistance gene guards the susceptibility gene SWEET14 of rice against exploitation by bacterial blight pathogen. Plant Commun. 100164.

Ma, W., Xu, X., Cai, L., Cao, Y., Haq, F., Alfano, J.R., Zhu, B., Zou, L., and Chen, G. (2020). A *Xanthomonas oryzae* type III effector XopL causes cell death through mediating ferredoxin degradation in *Nicotiana benthamiana*. Phytopathology Res. **2**:1–12.

Moscou, M.J., and Bogdanove, A.J. (2009). A simple cipher governs DNA recognition by TAL effectors. Science **326**:1501.

Ogata, T., Kida, Y., Arai, T., Kishi, Y., Manago, Y., Murai, M., and Matsushita, Y. (2012). Overexpression of tobacco ethylene response factor *NtERF3* gene and its homologues from tobacco and rice induces hypersensitive response-like cell death in tobacco. J. Gen. Plant Pathol. **78**:8–17.

Oh, S.K., Park, J.M., Joung, Y.H., Lee, S., Chung, E., KIM, S.Y., Yu, S.H., and Choi, D. (2005). A plant EPF-type zinc-finger protein, *CaPIF1*, involved in defence against pathogens. Mol. Plant Pathol. **6**:269–285.

Peng, Z., Hu, Y., Zhang, J., Huguet-Tapia, J.C., Block, A.K., Park, S., Sapkota, S., Liu, Z., Liu, S., and White, F.F. (2019). *Xanthomonas translucens* commandeers the host rate-limiting step in ABA biosynthesis for disease susceptibility. Proc. Natl. Acad. Sci. USA **116**:20938–20946.

Römer, P., Hahn, S., Jordan, T., Strauß, T., Bonas, U., and Lahaye, T. (2007). Plant pathogen recognition mediated by promoter activation of the pepper *Bs3* resistance gene. Science **318**:645–648.

Sambrook, J., and Russell, D.W. (2001). Molecular Cloning - A Laboratory Manual (Cold Spring Harbor, NY: Cold Spring Harbor Laboratory Press).

Schultink, A., Qi, T., Lee, A., Steinbrenner, A.D., and Staskawicz, B. (2017). Roq1 mediates recognition of the *Xanthomonas* and *Pseudomonas* effector proteins XopQ and HopQ1. Plant J. **92**:787–795. https://doi.org/10.1111/tpj.13715.

Schwartz, A.R., Potnis, N., Timilsina, S., Wilson, M., Patané, J., Martins, J., Jr., Minsavage, G.V., Dahlbeck, D., Akhunova, A., and Almeida, N. (2015). Phylogenomics of *Xanthomonas* field strains infecting pepper and tomato reveals diversity in effector repertoires and identifies determinants of host specificity. Front. Microbiol. **6**:535.

Schwessinger, B., and Ronald, P.C. (2012). Plant innate immunity: perception of conserved microbial signatures. Annu. Rev. Plant Biol. **63**:451–482.

Senthil-Kumar, M., and Mysore, K.S. (2013). Nonhost resistance against bacterial pathogens: retrospectives and prospects. Annu. Rev. Phytopathol. **51**:407–427.

Shi, H., Wang, X., Ye, T., Chen, F., Deng, J., Yang, P., Zhang, Y., and Chan, Z. (2014). The Cysteine2/Histidine2-Type transcription factor *ZINC FINGER OF ARABIDOPSIS THALIANA6* modulates biotic and abiotic stress responses by activating salicylic acid-related genes and C-REPEAT-BINDING FACTOR genes in *Arabidopsis*. Plant Physiol. **165**:1367–1379.

Strauß, T., van Poecke, R.M., Strauß, A., Römer, P., Minsavage, G.V., Singh, S., Wolf, C., Strauß, A., Kim, S., and Lee, H.-A. (2012). RNA-seq pinpoints a *Xanthomonas* TAL-effector activated resistance gene in a large-crop genome. Proc. Natl. Acad. Sci. USA **109**:19480–19485.

Sun, S.-J., Guo, S.-Q., Yang, X., Bao, Y.-M., Tang, H.-J., Sun, H., Huang, J., and Zhang, H.-S. (2010). Functional analysis of a novel Cys2/His2-type zinc finger protein involved in salt tolerance in rice. J. Exp. Bot. **61**:2807–2818.

Tang, D., Wang, G., and Zhou, J.-M. (2017). Receptor kinases in plant-pathogen interactions: more than pattern recognition. The Plant Cell **29**:618–637.

Tian, D., Wang, J., Zeng, X., Gu, K., Qiu, C., Yang, X., Zhou, Z., Goh, M., Luo, Y., Murata-Hori, M., et al. (2014). The rice TAL effector–dependent resistance protein XA10 triggers cell death and calcium depletion in the endoplasmic reticulum. The Plant Cell **26**:497–515.

Uma, B., Rani, T.S., and Podile, A.R. (2011). Warriors at the gate that never sleep: non-host resistance in plants. J. Plant Physiol. **168**:2141–2152.

Wang, C., Zhang, X., Fan, Y., Gao, Y., Zhu, Q., Zheng, C., Qin, T., Li, Y., Che, J., Zhang, M., et al. (2015). XA23 is an executor R protein and confers broad-spectrum disease resistance in rice. Mol. Plant **8**:290–302.

Wang, J., Tian, D., Gu, K., Yang, X., Wang, L., Zeng, X., and Yin, Z. (2017). Induction of *Xa10*-like genes in rice cultivar Nipponbare confers disease resistance to rice bacterial blight. Mol. Plant-microbe Interact. **30**:466–477.

Wei, C.-F., Kvitko, B.H., Shimizu, R., Crabill, E., Alfano, J.R., Lin, N.-C., Martin, G.B., Huang, H.-C., and Collmer, A. (2007). A *Pseudomonas syringae* pv. tomato DC3000 mutant lacking the type III effector HopQ1-1 is able to cause disease in the model plant *Nicotiana benthamiana*. Plant J. **51**:32–46.

Wright, K.M., Duncan, G.H., Pradel, K.S., Carr, F., Wood, S., Oparka, K.J., and Santa Cruz, S. (2000). Analysis of the N gene hypersensitive response induced by a fluorescently tagged tobacco mosaic virus. Plant Physiol. **123**:1375–1386.

Xu, D.-Q., Huang, J., Guo, S.-Q., Yang, X., Bao, Y.-M., Tang, H.-J., and Zhang, H.-S. (2008). Overexpression of a TFIIIA-type zinc finger protein gene ZFP252 enhances drought and salt tolerance in rice (*Oryza sativa* L.). FEBS Lett. **582**:1037–1043.

Xu, X., Xu, Z., Ma, W., Haq, F., Li, Y., Shah, S.M.A., Zhu, B., Zhu, C., Zou, L., and Chen, G. (2021). TALE-triggered and iTALE-suppressed *Xa1* resistance to bacterial blight is independent of *OsTFIIAγ1* or *OsTFIIAγ5* in rice. J. Exp. Bot.

Xu, Z., Xu, X., Gong, Q., Li, Z., Li, Y., Wang, S., Yang, Y., Ma, W., Liu, L., Zhu, B., et al. (2019). Engineering broad-spectrum bacterial blight resistance by simultaneously disrupting variable TALE-binding elements of multiple susceptibility genes in rice. Mol. Plant **12**:1434–1446.

Xue, J., Lu, Z., Liu, W., Wang, S., Lu, D., Wang, X., and He, X. (2020). The genetic arms race between plant and *Xanthomonas*: lessons learned from TALE biology. Sci. China Life Sci. **64**:51–65.

Yang, B., and White, F.F. (2004). Diverse members of the AvrBs3/PthA family of type III effectors are major virulence determinants in bacterial blight disease of rice. Mol. Plant-microbe Interact. **17**:1192–1200.

Yang, B., Sugio, A., and White, F.F. (2006). *Os8N3* is a host disease-susceptibility gene for bacterial blight of rice. Proc. Natl. Acad. Sci. USA **103**:10503–10508.

Yin, J., Wang, L., Zhao, J., Li, Y., Huang, R., Jiang, X., Zhou, X., Zhu, X., He, Y., and He, Y. (2020). Genome-wide characterization of the C2H2 zinc-finger genes in *Cucumis sativus* and functional analyses of four *CsZFPs* in response to stresses. BMC Plant Biol. **20**:1–22.

Yu, Y.-H., Li, X.-Z., Wu, Z.-J., Chen, D.-X., Li, G.-R., Li, X.-Q., and Zhang, G.-H. (2016). VvZFP11, a Cys_2His_2-type zinc finger transcription factor, is involved in defense responses in *Vitis vinifera*. Biol. Plant **60**:292–298.

Zeng, X., Tian, D., Gu, K., Zhou, Z., Yang, X., Luo, Y., White, F.F., and Yin, Z. (2015). Genetic engineering of the *Xa10* promoter for broad-spectrum and durable resistance to *Xanthomonas oryzae* pv. *Oryzae*. Plant Biotechnol. J. **13**:993–1001.

Zhang, H., Gao, X., Zhi, Y., Li, X., Zhang, Q., Niu, J., Wang, J., Zhai, H., Zhao, N., and Li, J. (2019). A non-tandem CCCH-type zinc-finger protein, IbC3H18, functions as a nuclear transcriptional activator and enhances abiotic stress tolerance in sweet potato. New Phytol. **223**:1918–1936.

Zhang, J., Yin, Z., and White, F. (2015). TAL effectors and the executor *R* genes. Front. Plant Sci. **6**:641.

Zhang, Q., Wang, C., Zhao, K., Zhao, Y., Caslana, V., Zhu, X., Li, D., and Jiang, Q. (2001). The effectiveness of advanced rice lines with new resistance gene *Xa23* to rice bacterial blight. Rice Genet. Newsl. **18**:71–72.

Zhao, B., Lin, X., Poland, J., Trick, H., Leach, J., and Hulbert, S. (2005). A maize resistance gene functions against bacterial streak disease in rice. Proc. Natl. Acad. Sci. USA **102**:15383–15388. https://doi.org/10.1073/pnas.0503023102.

Zhao, B., Ardales, E.Y., Raymundo, A., Bai, J., Trick, H.N., Leach, J.E., and Hulbert, S.H. (2004). The *avrRxo1* gene from the rice pathogen *Xanthomonas oryzae* pv. *oryzicola* confers a nonhost defense reaction on maize with resistance gene *Rxo1*. Mol. Plant-microbe Interact. **17**:771–779.

Zhao, T., Wu, T., Zhang, J., Wang, Z., Pei, T., Yang, H., Li, J., and Xu, X. (2020). Genome-wide analyses of the genetic screening of C_2H_2-type zinc finger transcription factors and abiotic and biotic stress responses in tomato (*Solanum lycopersicum*) based on RNA-seq data. Front. Genet. **11**:540.

Zhuang, H., Wang, H.-L., Zhang, T., Zeng, X.-Q., Chen, H., Wang, Z.-W., Zhang, J., Zheng, H., Tang, J., and Ling, Y.-H. (2020). *NONSTOP GLUMES1* encodes a C2H2 zinc finger protein that regulates spikelet development in rice. The Plant Cell **32**:392–413.

Zou, L., Wang, X., Xiang, Y., Zhang, B., Li, Y.-R., Xiao, Y., Wang, J., Walmsley, A.R., and Chen, G. (2006). Elucidation of the hrp clusters of *Xanthomonas oryzae* pv. *oryzicola* that control the hypersensitive response in nonhost tobacco and pathogenicity in susceptible host rice. Appl. Environ. Microbiol. **72**:6212–6224.

He, F., Zhang, F., Sun, W., Ning, Y., and Wang, G. (2018). A versatile vector toolkit for functional analysis of rice genes. Rice **11**:1–10.

Plant Communications, Volume *3*

Supplemental information

A *Xanthomonas* transcription activator-like effector is trapped in non-host plants for immunity

Fazal Haq, Xiameng Xu, Wenxiu Ma, Syed Mashab Ali Shah, Linlin Liu, Bo Zhu, Lifang Zou, and Gongyou Chen

Supplemental Information

The following Supplemental Information is available for this article:

Supplemental Methods

Methods S1. DNA manipulation and plasmid construction.

DNA gel extraction and plasmid miniprep kits were purchased from Axygen (Beijing, China). DNA polymerases, restriction endonucleases, and molecular weight markers were obtained from TaKaRa (Dalian, China). DNA ligase was purchased from Thermo Fisher Scientific (USA), and Ni-NTA purification resin was provided by Shanghai Yisheng Biotechnology Co., Ltd. Reverse transcription PCR and fluorescent quantitative PCR kits were purchased from TransGen Biotech (Beijing) Co., Ltd. Primers were designed with Primer Premier 5 Design Program (Premier Biosoft International, Palo Alto, CA, USA) and were synthesized by Generay (Shanghai, China). The constructs were confirmed by Sanger sequencing, which was a service provided by Biosune (Shanghai, China).

Cloning *Xoo* TALEs and *avrXa10* derivatives. For transient expression assays in *N. benthamiana*, *Xoo* TALEs were cloned in binary vector pHB (Mao et al., 2005), which contains the 35S promoter and a flag epitope upstream of the polylinker. The N- and C-terminal ends of *avrXa10* were amplified from plasmid pZW-avrXa10 (Zhu et al., 1998) using the primer sets avrXa10-F/avrXa10-N-R and avrXa10-C-F/avrXa10-R with *Hin*dIII/*Sph*I and *Sph*I/*Xba*I sites (Table S3), respectively. The amplified fragments were purified, sequenced and digested with *Hin*dIII/*Sph*I (N-terminus) and *Sph*I/*Xba*I (C-terminus). The two digested fragments were ligated together and cloned into pHB digested with *Hin*dIII and *Xba*I. The resulting plasmid was named pHB-AvrXa10ΔCRR and contained the N and C-terminal regions of AvrXa10, but lacked the *Sph*I fragment containing the central repeat region (CRR). The central repeat region (CRR) of AvrXa10 was digested from plasmid pZW-avrXa10 with *Sph*I and cloned into *Sph*I-digested pHB-avrXa10ΔCRR plasmid, resulting in pHB-AvrXa10 (pHB with full-length *avrXa10*). Clones containing the 17 *Xoo tal* genes in pBluescript (unpublished data) were digested with *Bam*HI to release the CRR regions and portions of the N and C-terminal ends (N+CRR+C fragments). The pHB-AvrXa10 construct was digested with *Bam*HI to release the CRR and flanking regions, and the N+CRR+C fragments from the 17 *tal* genes were individually inserted into the *Bam*HI site, giving rise to 17 *tal* genes in pHB (Table S2).

To construct the AD mutant of *avrXa10*, pHB-avrXa10 was digested with *Sal*I (unique site located after the C-terminal *Sph*I site in *avrXa10*) and *Xba*I to release the AD domain along with two nuclear localization signal (NLS) motifs. The NLS motifs (~176 bp upstream of the AD domain) were amplified from pHB-avrXa10 using primers avrXa10-C(ΔAD)-F/avrXa10-C(ΔAD)-R; a stop codon was included in the latter primer (Table S3). The PCR product was sequenced, digested with *Sal*I and *Xba*I, and cloned into *Sal*I/*Xba*I-digested pHB-avrXa10, resulting in pHB-avrXa10ΔAD.

Methods S2. Transient expression assays in tobacco and rice.

The binary vector pHB was used to clone and express TALEs in *N. benthamiana* (Mao et al., 2005). pHB contains the CaMV 35S promoter and a flag-tag upstream of the polylinker, and *tal* genes were cloned into the vector polylinker. pHB constructs were transformed into *Agrobacterium* strain EHA105 by freeze-thaw method; and *Agrobacterium* transformants were cultured in LB medium to OD_{600}=1.5. Cultures were harvested by centrifugation, washed and resuspended in infiltration buffer (10 mM $MgCl_2$, 0.2 mM acetosyringone and 200 mM MES, pH 5.6) to a final concentration of OD_{600}=1.0. Buffer-supplemented *Agrobacterium* strains were incubated at room temperature for 1 h and then infiltrated (OD_{600} = 1.0) into *N. benthamiana* leaves with needleless syringes for transient expression assays.

Transient expression assays in rice. For transient expression in rice protoplasts, pRTVcHA was used, which contains the maize ubiquitin (Ubi) 1 promoter and a C-terminal HA-tag epitope (He et al., 2018). The full-length coding sequence (CDS) of *Nb20731g* was amplified from tobacco cDNA using primer pairs 20731g-F/20731g-R (Table S3). The full-length CDS of *LOC_Os03g41110*, *LOC_Os09g26210*, *LOC_Os08g44190*, *LOC_Os03g13600* was amplified from rice (Nipponbare) cDNA using primer pairs Os41110-F/Os41110-R, Os26210-F/Os26210-R, Os44190-F/Os44190-R, Os13600-F/Os13600-R, respectively (Table S3). The PCR product was purified, sequenced and cloned into the *Bam*HI/*Not*I sites of pRTVcHA, giving rise to pRTVcHA-20731g. The full-length CDS of rice gene *Xa10* was amplified from pB-Xa10 (Table S2) using primer pairs Xa10-F/Xa10-R (Table S3). The PCR product was purified, sequenced and cloned into the *Bam*HI/*Not*I sites of pRTVcHA, giving rise to pRTVcHA-Xa10.

Methods S3. Western blot assays.

In planta expression of the TALE and dTALE proteins cloned in pHB vector was confirmed by western blotting using the flag-tag epitope. Briefly, infiltrated leaf samples were collected at 48 hpi and macerated in protein lysis buffer (50 mM Tris-MES, 0.5 M sucrose, 1 mM $MgCl_2$, 10 mM EDTA, 5 mM DTT and protease inhibitor cocktail, pH 8.0). The protein samples were separated on 8% SDS-PAGE and transferred to PVDF membranes for immunoblotting using anti-FLAG serum (Transgene, Beijing, China) as described previously (Haq et al., 2020).

Methods S4. AvrXa10 delivery into plant cells via *Xanthomonas*

The Plasmid pHZW-AvrXa10 (Table S2), which contains a FLAG-tag epitope in the *c*-terminus of AvrXa10, was electroporated (2.5 kv, 4 ms) into *Xanthomonas axonopodis* pv. *glycines* (*Xag*) strain ATCC43911. The expression of AvrXa10 was confirmed by western blot using mouse anti-flag antibody as described previously (Haq et al., 2020). The strain ATCC43911 containing AvrXa10 or empty vector pHM1 were cultured overnight in NA liquid medium. The bacterial cells were collected via centrifugation, washed twice with 10mM $MgCl_2$ and, resuspended in 10mM $MgCl_2$ to OD_{600} = 0.2. The suspensions were infiltrated into *N. benthamiana* leaves with needleless syringe. Infiltration of simply 10mM buffer served as a mock. The leaf phenotype was photographed at 24hpi. This experiments were repeated three times with similar results.

Methods S5. RNA isolation, RT-PCR and qRT-PCR.

Total RNA was extracted from inoculated leaf tissue using RNAiso plus reagent (Takara, China). The quality of RNA was checked with the NanoDrop spectrophotometer (Eppendorf) and then reverse transcribed using EasyScript® One Step gDNA Removal and cDNA Synthesis Supermix (TransGen). Semi-quantitative expression was carried out by RT-PCR using 2x Taq PCR StarMix (TaKaRa). PCR amplification conditions were as follows: 98˚C for 15 s, 60˚C for 30 s, and 72˚C for 12 s; amplification continued for a total of 28 cycles. Real-time quantitative PCR (RT-qPCR) was performed using TransStart® Tip Green qPCR SuperMix (TransGen) and the ABI 7500 quantitative PCR system. *NbEF1α* was used as an internal control. Primers used for qRT-PCR are listed in Table S3.

Methods S6. Protein expression and purification.

The construct pET30a-pthXo1 was used to construct pET30a-avrXa10. In a previous report (Ma et al., 2018) a multi-step process was used to clone *pthXo1* in pET30a. For cloning avrXa10 in pET30a, the central portion of *pthXo1* in pET30a-pthXo1 was replaced with avrXa10 at conserved *Not*I and *Sal*I sites to give rise pET30a-avrXa10 (His-AvrXa10). The *Sal*I site is conserved in TALE genes containing the C-terminal flag sequence. The generated construct was then introduced into *E. coli* BL21 (DE3). The bacteria were grown in LB medium containing 25 µg/ml kanamycin at 37℃ to an OD_{600} of 0.5. The expression of His-AvrXa10 was induced by adding 0.5mM IPTG (isopropyl-b-D-thiogalactopyranoside) and incubating at 16℃ for 14 h. Bacterial cells were incubated on ice for 20 minutes to stop growth and then harvested by centrifugation at 5000 rpm for 10 min. The cells were washed one time in PBS and resuspended in 10 mM PBS (pH 7.5) supplemented with cocktail and phenylmethylsulfonyl fluoride (PMSF). The cells were sonicated for 10-20 min and then centrifuged at 8000 rpm for 20 min at 4℃. Proteins were purified from the supernatant using Ni-NTA His Resin (Shanghai Yisheng Biotechnology Co., Ltd.) according to the manufacturer's instructions.

Methods S7. Subcellular localization of NbZnFP1.

The subcellular localization of NbZnFP1 was investigated. NbZnFP1 was amplified using primers NbZnFP1-YFP-F/ NbZnFP1-YFP-R and cloned in the YFP vector with *Kpn*I and *Sma*I sites, resulting in construct NbZnFP1-YFP. *Agrobacterium* strain GV3101 containing the construct YFP-NbZnFP1 or YFP (empty vector) were transiently expressed in *Nb* leaves. At 2 dpi, Nb leaves were visualized with a Leica confocal microscope; the excitation wavelength for YFP was 488 nm, and 520–550 nm was used for emission.

Supplemental Figures

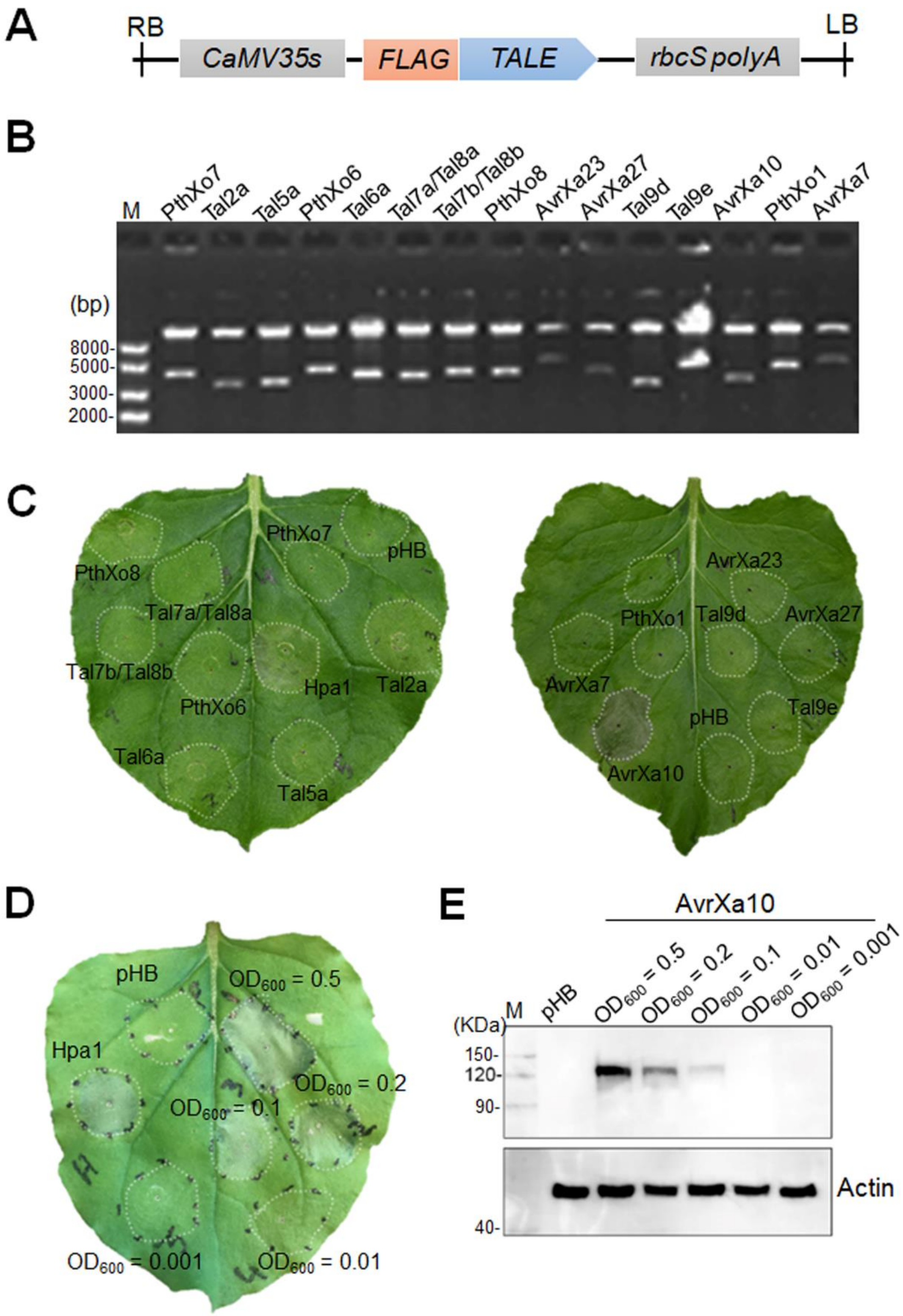

Figure S1. Analysis of HR-like cell death in *N. benthamiana*. (A) Schematic diagram showing approach for cloning *TALEs* in the binary vector pHB. Abbreviations: CaMV 35S, cauliflower mosaic 35S promoter; FLAG, N-terminal flag epitope tag driven by 35S promoter; TALE, insertion site for *Xoo*

TALE gene; rbcS, ribulose-1,5-bisphosphate carboxylase, small subunit; polyA, polyadenylation site. **(B)** Confirmation of *tal*-gene insert in pHB vector by restriction digestion with *BamH*I. **(C)** Phenotype of *Nb* leaves expressing TALE proteins; the empty vector pHB was transformed as a negative control. *Agrobacterium* strains containing different TALE constructs or empty pHB vector were infiltrated into *Nb* leaves at OD_{600}=1.0. Leaves were photographed at three days post-infiltration (dpi). **(D)** *Agrobacterium* carrying AvrXa10 was infiltrated into *Nb* leaves at OD_{600}=0.5, 0.2, 0.1, 0.01 and 0.001. *Agrobacterium* strains carrying Hpa1 and empty pHB served as positive and negative controls, respectively. *Nb* leaves were photographed at 4 dpi. **(E)** Immunodetection of flag-tagged AvrXa10 in *N. benthamiana* at different OD values at 3 dpi. pHB was a negative control, and Actin was used as a loading control.

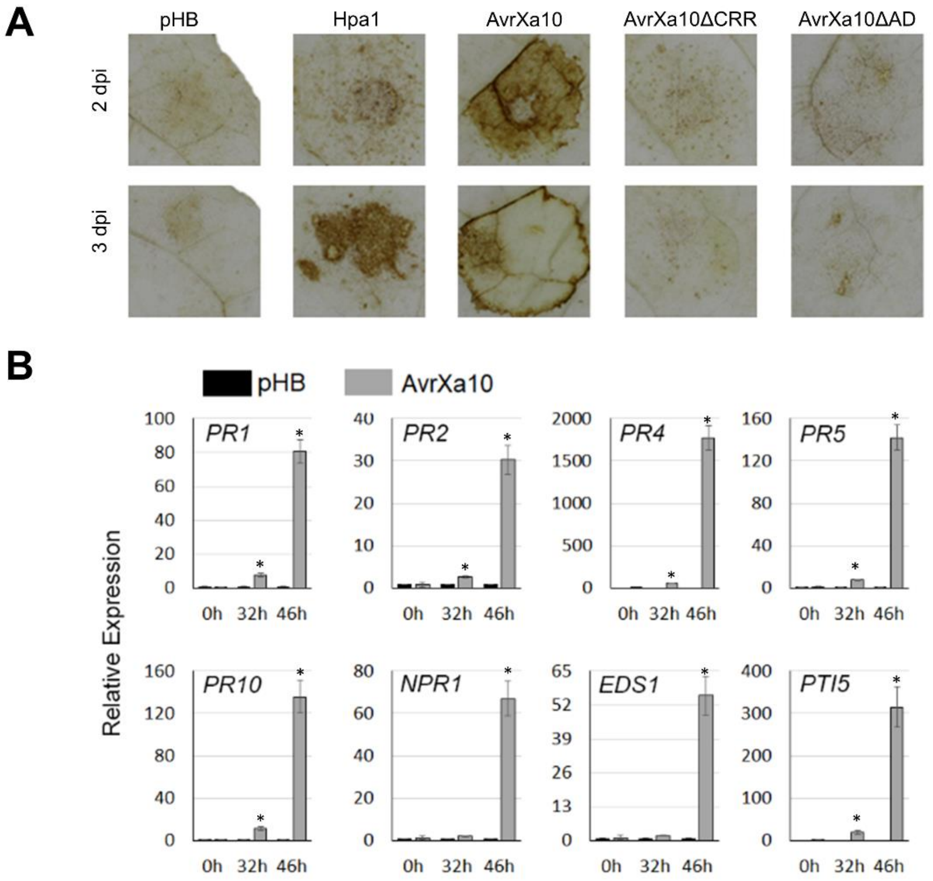

Figure S2. Detection of ROS and expression of defense-related genes in *N. benthamiana* leaves agro-infiltrated with pHB-AvrXa10 and derivatives. (A) ROS detection in *Nb* leaves infiltrated with *Agrobacterium* containing pHB-AvrXa10, pHB-HpaI (positive control) pHB-AvrXa10ΔCRR, pHB-AvrXa10ΔAD, and pHB at 2 and 3 dpi. Agro-infiltrated leaves of *Nb* were collected and incubated in DAB (1 mg/ml), 0.05% Tween-20 and 10 mM sodium phosphate buffer (pH 7.0) in darkness for 6-8 h. Chlorophyll was removed as described (Ma *et al.*, 2020), and leaves were stored in 65% ethanol and photographed. HpaI and empty pHB were used as positive and negative controls, respectively. **(B)** RT-qPCR expression analysis of defense-related genes, *PR1, PR2*, *PR4*, *PR5, PR10*, *EDS1*, *NPR1*, and *PTI5* in *N. benthamiana* leaves expressing AvrXa10 or pHB (control). Expression was monitored at 0, 32, and 46 hpi. Error bars represent means and means ± SD (n = 3), and columns labeled with asterisks show significant differences (P≤0.01). The results shown are representative of three independent replicates.

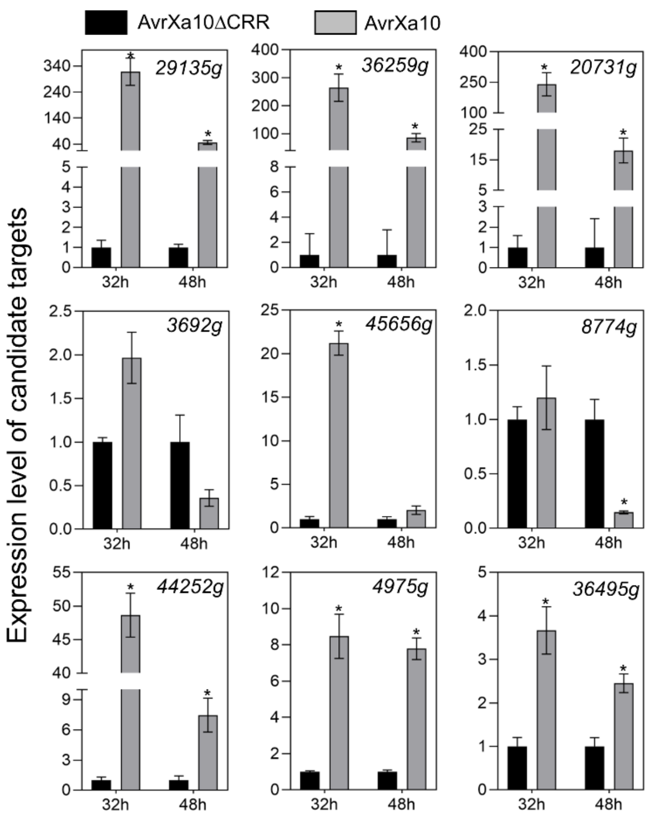

Figure S3. RT-qPCR analysis of putative AvrXa10 target genes in *N. benthamiana*. Leaves of four-week-old *Nb* were infiltrated with *Agrobacterium* strains carrying AvrXa10 or AvrXa10ΔCRR and collected at 32 and 48 hpi for RNA isolation. *NbEF1a* was used as an internal control. Error bars represent means ± SD (*n* =3), and columns labeled with asterisks show significant differences (*P*<0.05) in the AvrXa10-expressing leaves as compared to the AvrXa10ΔCRR control. The results shown are representative of three independent replicates.

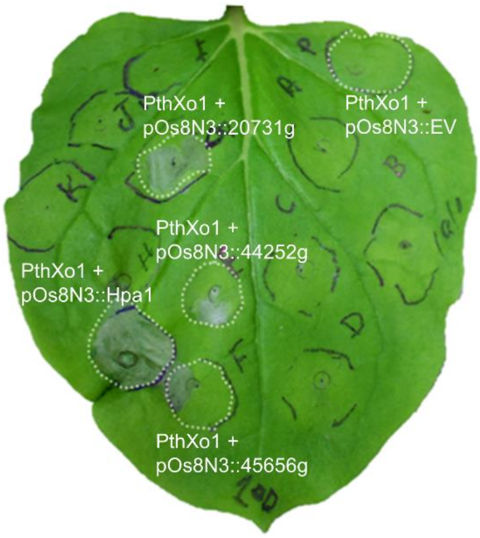

Figure S4. *in-vivo* reporter assay for HR in *N. benthamiana*. *Agrobacterium* strains containing the effector construct (pHB-pthXo1) and one of the six reporter constructs were infiltrated into five-week-old *Nb* leaves, which were evaluated for the HR at 4-7 dpi. Co-infiltration of pHB-PthXo1 with

pOs8N3-EV or pOs8N3-Hpa1 served as a negative and positive control, respectively. Representative results were chosen from five independent experiments.

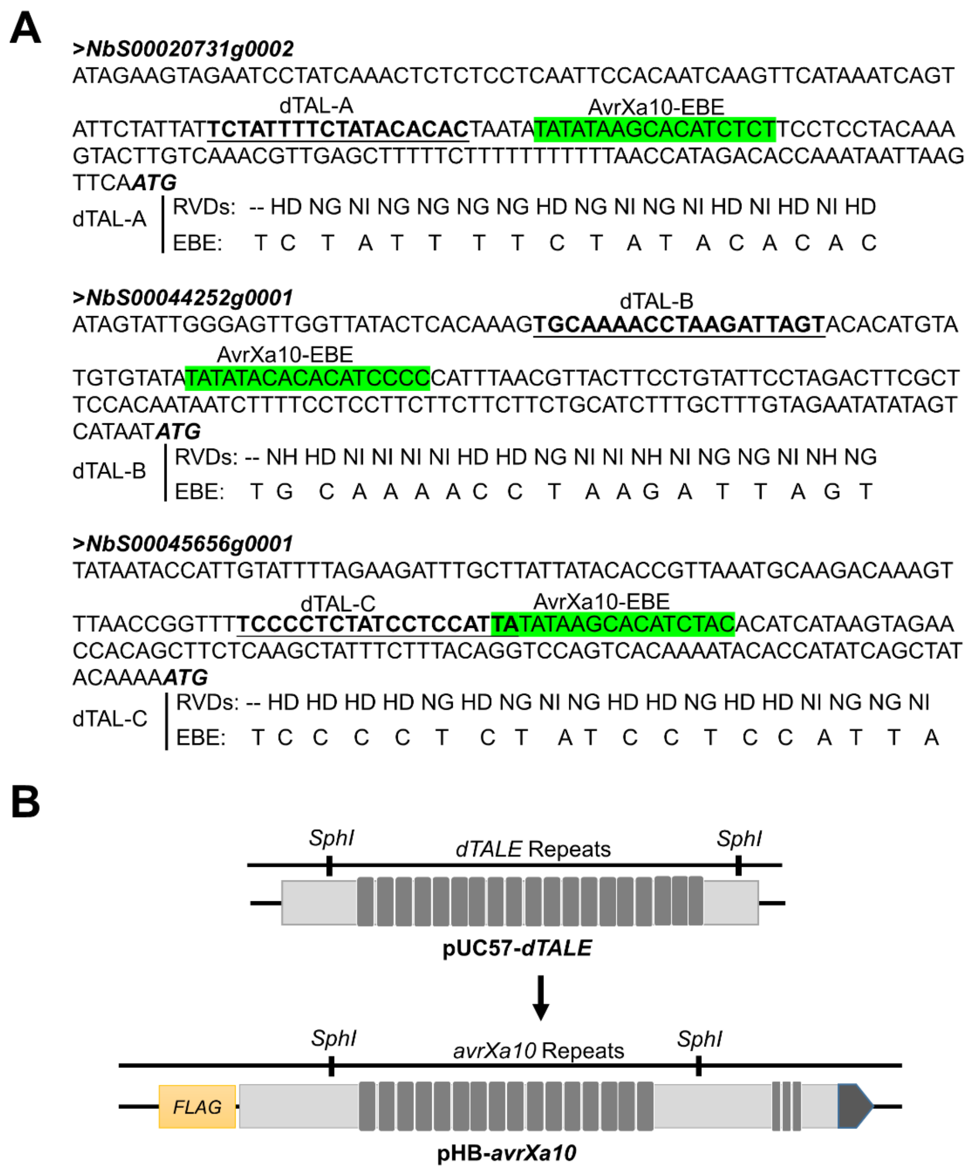

Figure S5. Construction of artificial dTALEs to activate the expression of *Nb20731g*, *Nb44252g* and *Nb45656g*. (A) Promoter sequences of *Nb20731g*, *Nb44252g* and *Nb45656g*. The bold underscored regions indicate sites for dTALE insertion. dTAL-A was designed to target an18-bp region upstream of AvrXa10-EBE (green-shaded nucleotides) in the promoter of *Nb20731g*. The dTAL-B and dTAL-C were designed to target 19-bp regions upstream of AvrXa10-EBE (green-shaded nucleotides) in the promoters of *Nb44252g* and *Nb45656g*, respectively. The repeat variable diresidues (RVDs) of each dTALE and their target EBE are shown. **(B)** Schematic map showing the cloning of dTALEs into pHB vector. The *SphI* fragment of each dTALE was replaced with the *Sph*I fragment of AvrXa10 in the pHB-AvrXa10 constructs to produce pHB-dTAL-A, pHB-dTAL-B, and pHB-dTAL-C.

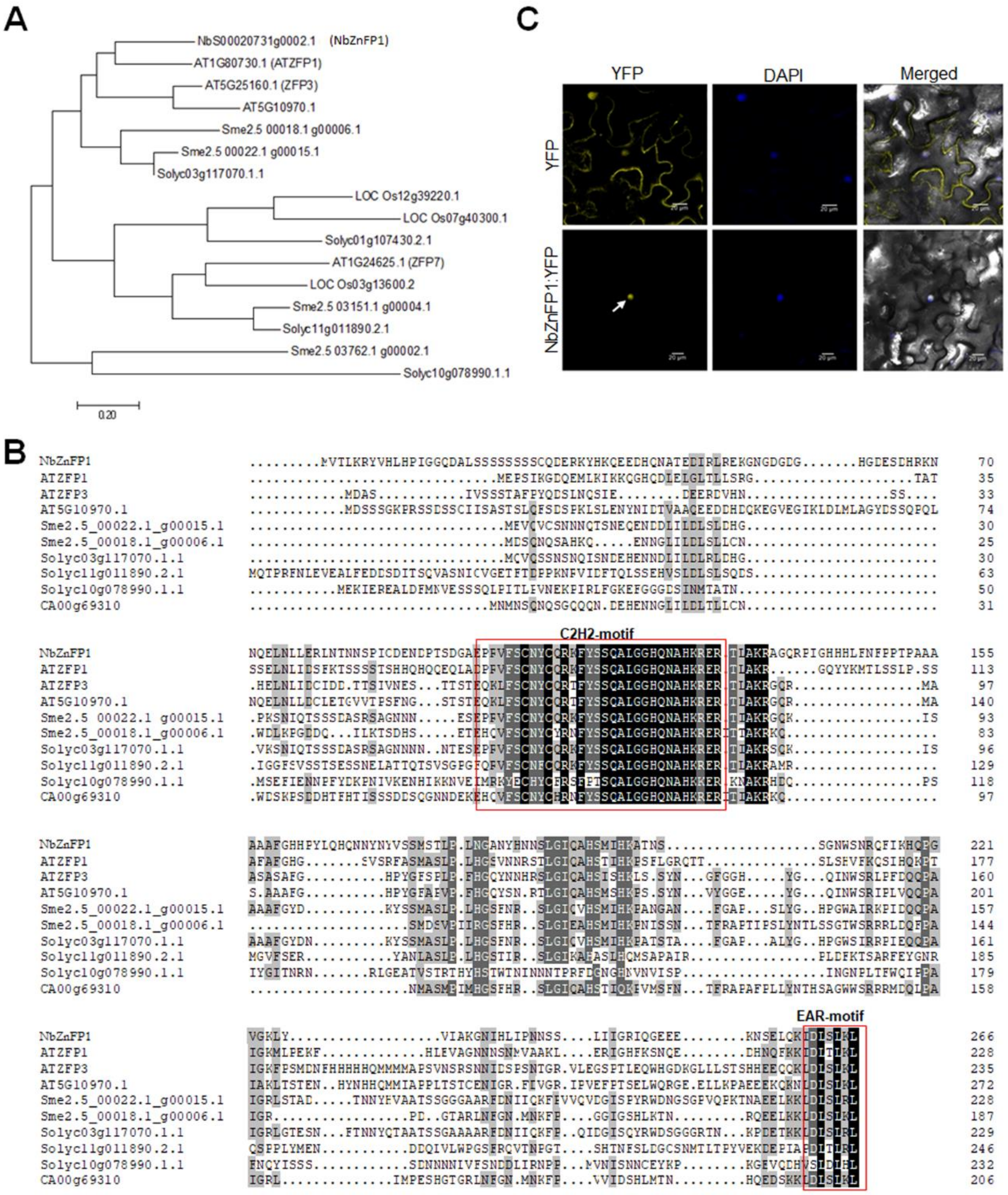

Figure S6. Phylogenetic relatedness of NbZnFP1 with related zinc finger proteins (ZnFPs) and localization of NbZnFP1 in *N. benthamiana*. (A) Relatedness of NbZnFP1 in *N. benthamiana* to ZnFPs in *Arabidopsis thaliana* (ATZFP1, ZFP3, ZFP7), *Solanum melongena*, *S. lycopersicum*, and *Capsicum annuum*. Phylogenetic relationships were based on full-length amino acid sequence data., and the tree was generated with MEGA 7.0.14. **(B)** Alignment of the NbZnFP1 sequence with orthologous proteins in *A. thaliana*, *S. melongena*, *S. lycopersicum,* and *C. annuum* with DNAMAN (https://www.lynnon.com/dnaman.html). Identical amino acids are indicated in white font with black background, and similar amino acids are shaded in dark or light gray. **(C)** Subcellular localization of NbZnFP1 in *N. benthamiana*. *Nb* leaves were infiltrated with *Agrobacterium* containing pYFP (control) or NbZnFP1::YFP, and images were captured at 48 hpi by confocal microscopy. Nuclei were stained with 4′,6-diamidino-2-phenylindole (DAPI). The white arrow indicates the nucleus. Scale bars: 20 µm.

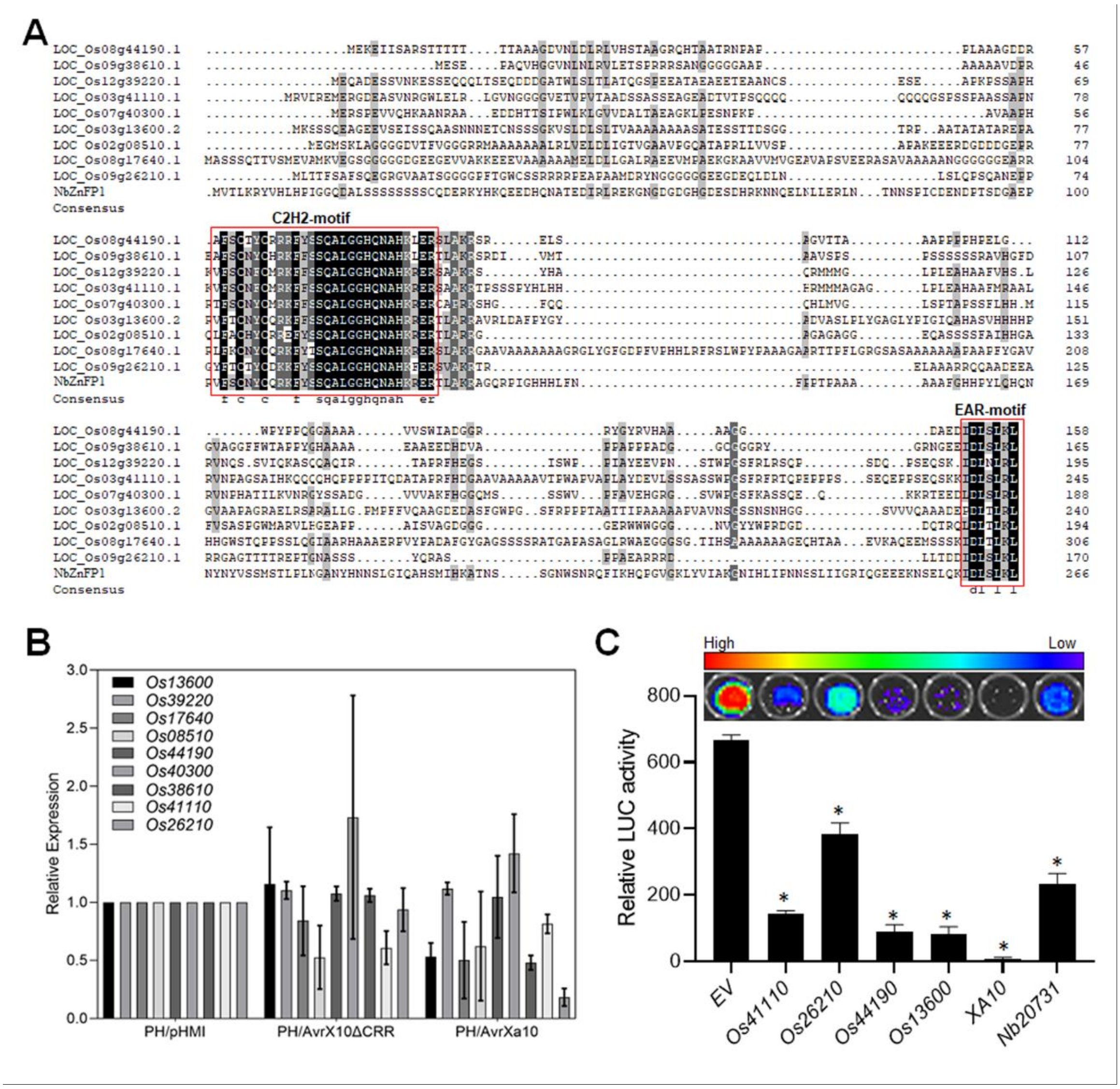

Figure S7. Alignment of NbZnFP1 orthologues in rice and expression assays. (A) Alignment of NbZnFP1 with nine orthologous proteins in rice cv. Nipponbare with DNAMAN. Proteins included: Os08g44190.1, Os9g38610.1, Os12g39220.1, Os03g41110.1, Os07g40300.1, Os03g13600.2, Os02g08510.1, Os08g17640.1, Os09g26210.1, and NbZnFP1. Identical amino acids are indicated in white font with black background; similar amino acids are shaded in dark or light gray. The C2H2 and EAR motifs are shown. **(B)** Expression of *NbZnFP1* homologs in rice inoculated with the derivatives of the *tal*-free strain, *Xoo* PH (Table S2). Leaves of three-week-old rice plants were infiltrated with PH strains carrying AvrXa10, AvrXa10ΔCRR, or pHM1 (empty vector), and collected at 24 hpi for RNA isolation. *OsActin* was used as an internal control. Error bars represent means and standard deviations (means ± SD) (n =3), and columns labeled with asterisks represent significant differences (P<0.05). **(C)** Transient expression of four *NbZnFP1* orthologue from rice cause cell death in rice protoplasts. Constructs pRTVcHA-Os41110, pRTVcHA-Os26210, pRTVcHA-Os44190, pRTVcHA-Os13600 and pRTVcHA (EV, empty vector) were co-expressed with the LUC reporter construct, pRTVcVC-LUC, in rice protoplasts. Co-transfection of pRTVcHA-Xa10 (Xa10) with the LUC construct was used as a positive, cell-death inducing control. LUC activity was measured after 24 h of transfection using Promega LUC assay system (Promega Corp.). The images on the top of the graph show microtiter plates containing protoplasts expressing the constructs. The image of LUC fluorescence was taken with CCD imaging (IVIS spectrum, PerkinElmer, USA). The graph below show the relative LUC activity measured with luminometer (Tecan, M200). Cell death in protoplasts was monitored by reduction in luciferase activity. Error bars represent means ± SD, and columns labeled with an asterisk (*) represent significance at P<0.01.

Supplemental Tables

Table S1. The list of candidate targets of AvrXa10.

Gene ID	EBE Score[a]	EBE sequence	EBE to ATG (bp)	$\log_2$FC[b] (vs. AvrXaΔCRR)		Expression[c] (vs. AvrXaΔCRR)		Description
				32 hpi	48 hpi	32 hpi	48 hpi	
NbS00029135g0002	-7.10287	TATATAAGCACATACCC	102	6.268363	8.359675	317.90	46.78	uncharacterized protein
NbS00036259g0008	-7.10287	TATATAAGCACATACCC	123	5.275453	Inf[d]	265.06	85.98	uncharacterized protein
NbS00020731g0002	-9.24073	TATATAAGCACATCTCT	77	Inf	9.240476	2.45	18.06	zinc finger protein 1-like
NbS00016355g0012	-10.0296	TATATAATCACGTGCCC	33	6.875339	8.508549	-	-	bidirectional sugar transporter SWEET6a-like
NbS00003692g0004	-10.7409	TATATAAGCACGTCTTC	16	6.282507	4.117477	1.98	0.36	transcription factor bHLH87-like
NbS00045656g0001	-11.0257	TATATAAGCACATCTAC	81	3.562645	4.422442	21.22	2.03	ethylene-responsive transcription factor 4-like
NbS00008774g0001	-11.2694	TATATAAGCATGTCTCT	115	4.509918	5.534762	1.20	0.14	transcription repressor OFP12-like
NbS00044252g0001	-11.4589	TATATACACACATCCCC	9	5.418556	8.997659	48.65	7.46	GDSL esterase/lipase At3g26430-like
NbS00004975g0004	-11.6268	TATATAAACACCAATCT	157	4.509345	6.526783	8.47	7.79	small auxin-up protein 58
NbS00036496g0001	-11.6283	TATATATACACATATCC	23	7.130504	8.262867	3.67	2.45	uncharacterized protein

[a]EBE score calculated via TALgetter tool; [b]$\log_2$-fold change values from RNA-seq data; [c]Expression values by qRT-PCR analysis; [d]Infinity value

Table S2. Bacterial strains and plasmids used in this study.

Strain or plasmid	Relevant characteristics	Source
**Strain**		
Escherichia coli		
DH5α	*F-*, *endA1*, *thi-1*, *recA1*, Φ80*lacZ*, Δ M15	Clontech
BL21	*F-*, *ompT*, *hsdS20*, *gal*, *dcm*(DE3)	Novagen
Agrobacterium tumefaciens		
EHA105	C58, pTiBo542DT-DNA, Rif^{r}	This lab
GV3101	C58, pTic58DT-DNA, Rif^{r}	This lab
Xanthomonas oryzae* pv. *oryzae		
PH	*tal*-free derivative of Xoo PXO99^{A}	(Ji et al., 2016)
**Plasmids**		
pHB	Binary vector, double 35S promoter, 3X N-terminal FLAG tag, Km^{r}	(Mao et al., 2005)
pUC57	Cloning vector, pUC19 derivative, Ap^{r}	ViewSolid Biotechnology, Beijing
pRTVcHA	*Ubi* promoter, c-terminal 4X-HA tag; used for transient expression in rice	(He et al., 2018)
pRTVcVC-LUC	*LUC* in pRTVcVC, mCherry, C-terminal cMyc-tag + mVenus-$C_{156\text{-}238}$	(Xu et al., 2021)
pET30a(+)	pBR322 origin, *lacI*, His-tag at C-terminus, Km^{r}	Novagen
pCAMBIA1381	Binary vector containing promoterless *gusA*; used for reporter assays, Km^{r}	
pTRV-RNA1 (pTRV1)	pTRV encoding replicase, movement protein and cysteine-rich protein, helper vector, Km^{r}	(Zhao et al., 2013)
pYL156-RNA2 (pTRV2)	TRV-based VIGS vector, Km^{r}	(Zhao et al., 2013)
pHM1	Broad-spectrum cosmid vector, Sp^{r}	(Hopkins et al., 1992)

pYFP	Binary vector with full-length YFP coding gene, c-Myc tag, Km^r	(Ma et al., 2018)
pZW-avrXa10	*avrXa10* in pBluescript II KS^+, contains FLAG epitope immediately downstream of the second *Sph*I site in the C-terminus of AvrXa10, Ap^r	(Zhu et al., 1998)
pHB-Hpa1	*hpa1* cloned in pHB	This study
pHB-hpa1	*hpa1* cloned in frame with N-terminal flag-tag, Km^r	(Ma et al., 2020)
pHB-AvrXa10	*avrXa10* cloned in frame with N-terminal flag-tag in pHB, Km^r	This study
pHB-AvrXa7	*avrXa7* cloned with N-terminal flag-tag in pHB, Km^r	This study
pHB-AvrXa27	*avrXa27* cloned with N-terminal flag-tag in pHB, Km^r	This study
pHB-PthXo1	*pthXo1* cloned with N-terminal flag-tag in pHB, Km^r	This study
pHB-pthXo7	*pthXo7* cloned with N-terminal flag-tag in pHB, Km^r	This study
pHB-tal2a	*tal2a* cloned with N-terminal flag-tag in pHB, Km^r	This study
pHB-tal4	*tal4* cloned with N-terminal flag-tag in pHB, Km^r	This study
pHB-tal5a	*tal5a* cloned with N-terminal flag-tag in pHB, Km^r	
pHB-pthXo6	*pthXo6* cloned with N-terminal flag-tag in pHB, Km^r	This study
pHB-tal6a	*tal6a* cloned with N-terminal flag-tag in pHB, Km^r	This study
pHB-tal7a	*tal7a* cloned with N-terminal flag-tag in pHB, Km^r	This study
pHB-tal7b	*tal7b* cloned with N-terminal flag-tag in pHB, Km^r	This study
pHB-tal8a	*tal8a* cloned with N-terminal flag-tag in pHB, Km^r	This study
pHB-tal8b	*tal8b* cloned with N-terminal flag-tag in pHB, Km^r	This study
pHB-pthXo8	*pthXo8* cloned with N-terminal flag-tag in pHB, Km^r	This study
pHB-avrXa23	*avrXa23* cloned with N-terminal flag-tag in pHB, Km^r	This study
pHB-tal9d	*tal9d* cloned with N-terminal flag-tag in pHB, Km^r	This study
pHB-tal9e	*tal9e* cloned with N-terminal flag-tag in pHB, Km^r	This study
pHB-AvrXa10ΔCRR	pHB containing N and C-terminal regions of AvrXa10; lacks *Sph*I fragment containing the CCR, Km^r	This study
pHB-AvrXa10ΔAD	pHB containing a truncated *avrXa10* that lacks the AD domain; Km^r	This study
pHB-dTALE$_{20731}$	*dTALE*$_{20731}$ cloned in pHB	This study
pHB-dTALE$_{44252}$	*dTALE*$_{44252}$ cloned in pHB	This study
pHB-dTALE$_{45656}$	*dTALE*$_{45656}$ cloned in pHB	This study
pHB-NbZnFP1	*NbZnFP1* cloned in pHB	This study

pET30a-pthXo1	*pthXo1* cloned in pET30a; contains His-tag at C-terminus, Km^r	(Ma et al., 2018)
pET30a-avrXa10	*avrXa10* cloned in pET30a; contains His-tag at C-terminus, Km^r	This study
pHZW-AvrXa10	pHM1 fused with pZWavrXa10 at *Hin*dIII, *lacZ* promoter upstream of *avrXa10*, Ap^r, Sp^r	(Zhu et al., 1998)
pZW- AvrXa10ΔCRR	*Sph*I central repeat region was deleted in pZW-avrXa10 and self-ligated, Ap^r	This study
pHZW-AvrXa10ΔCRR	pHM1 fused with pZWavrXa10ΔCRR at *Hin*dIII, *lacZ* promoter upstream of *avrXa10ΔCRR*, Ap^r, Sp^r	This study
p29135g::GUS	~1 kb promoter region of *29135g* cloned upstream of *gusA* in pCAMBIA1381	This study
p36259g::GUS	~1 kb promoter region of *36259g* cloned upstream of *gusA* in pCAMBIA1381	This study
p20731g::GUS	~1 kb promoter region of *20731g* cloned upstream of *gusA* in pCAMBIA1381	This study
p44252g::GUS	~1 kb promoter region of *44252g* cloned upstream of *gusA* in pCAMBIA1381	This study
p45656g::GUS	~1 kb promoter region of *45656g* cloned upstream of *gusA* in pCAMBIA1381	This study
p3692g::GUS	~1 kb promoter region of *3692g* cloned upstream of *gusA* in pCAMBIA1381	This study
pOs8N3::GUS	promoter region of *Os8N3* cloned upstream of *gusA* in pCAMBIA1381	(Cai et al., 2017)
pOs8N3::29135	*29135g* CDS in pCAMBIA1381, driven by *Os8N3* promoter, Km^r	This study
pOs8N3::36259g	*36259g* CDS in pCAMBIA1381, driven by *Os8N3* promoter, Km^r	This study
pOs8N3::20731g	*20731g* CDS in pCAMBIA1381, driven by *Os8N3* promoter, Km^r	This study
pOs8N3::44252g	*44252g* CDS in pCAMBIA1381, driven by *Os8N3* promoter, Km^r	This study
pOs8N3::45656g	*45656g* CDS in pCAMBIA1381, driven by *Os8N3* promoter, Km^r	This study

pOs8N3::3692g	*3692g* CDS in pCAMBIA1381, driven by *Os8N3* promoter, Km^r	This study
pOs8N3::hpa1	*hpa1* CDS in pCAMBIA1381, driven by *Os8N3* promoter, Km^r	This study
pOs8N3::EV	*Os8N3* promoter cloned in pCAMBIA1381, Km^r	This study
pYL156-NbZnFP1	Partial CDS of *NbZnFP1* (*Nb20731g*) cloned as a *Xba*I/*Bam*HI fragment in pYL156	This study
pGBKT7-20731g	*20731g* gene cloned in pGBKT7	This study
pRTVcHA-NbZnFP1	Tobacco gene *NbZnFP1* (*Nb20731g*) cloned as a *Bam*HI/*Not*I fragment in pRTVcHA	This study
pRTVcHA-Xa10	Rice gene *Xa10* cloned as a *Bam*HI/*Not*I fragment in pRTVcHA	This study
pRTVcHA-Os41110	Rice gene *LOC_Os03g41110* cloned as a *Bam*HI/*Not*I fragment in pRTVcHA	This study
pRTVcHA-Os26210	Rice gene *LOC_Os09g26210* cloned as a *Bam*HI/*Not*I fragment in pRTVcHA	This study
pRTVcHA-Os44190	Rice gene *LOC_Os08g44190* cloned as a *Bam*HI/*Not*I fragment in pRTVcHA	This study
pRTVcHA-Os13600	Rice gene *LOC_Os03g13600* cloned as a *Bam*HI/*Not*I fragment in pRTVcHA	This study
pB-Xa10	Rice *Xa10* gene in pHB vector	Lab collection
NbZnFP1-YFP	*NbZnFP1* in *Kpn*I/*Sma*I site of pYFP, c-myc tag, Km^r	This study

Abbreviations: Ap, ampicillin; Sp, spectinomycin; Km, Kanamycin; Rif, Rifampicin

Table S3. Primers used in this study.

Primer name	Sequence (5'-3'; restriction sites underlined)	Description
avrXa10-F	CCCAAGCTTATGGATCCCATTCGTT	Amplifies N-terminus of AvrXa10 for CRR mutant construction; contains *Hin*dIII/*Sph*I sites
avrXa10-N-R	TGCATTGCGCCATGCATGCACTG	
avrXa10-C-F	CGGATCAGGCGTCTTTGCATGCA	Amplifies C-terminus of AvrXa10 for CRR mutant; contains *Sph*I/*Xba*I sites
avrXa10-R	GCTCTAGATCAGATCGTCCCTCCG	
avrXa10-(ΔAD)-F	GCGTCGACCCAGCCCAATG	Amplifies 176 bp from C-terminus of AvrXa10; for AD mutant; contains *Sal*I/*Xba*I sites
avrXa10-C(ΔAD)-R	GCTCTAGATCAGGTACGCGGGCGTTTTAC	
NbPR1a-F	GGTGTAGAACCTTTGACCTGGG	qPCR primer for *NbPR1a*
NbPR1a-R	AAATCGCCACTTCCCTCAGC	
NbPR2-qRT-F	TAGAGAATACCTACCCGCCC	qPCR primer for *NbPR2*
NbPR2-qRT-R	GAGTGGAAGGTTATGTCGTGC	
NbPR4-qRT-F	GTGACGAACACAAGAACAGGAA	qPCR primer for *NbPR4*
NbPR4-qRT-R	CCACTCCATTTGTGTCCAAT	
NbPR5-qRT-F	TTATGGTGAGACTGGACTCCC	qPCR primer for *NbPR5*
NbPR5-qRT-R	CCACCAGATGCCTTCTTTG	
NbPR10-qRT-F	AGGAGTCAGGTGATGGTGGT	qPCR primer for *NbPR10*
NbPR10-qRT-R	TGAACATAGCCGAGACCTTC	
NbNPR1-qRT-F	TGAGATTCTGGAGCAAGCA	qPCR primer for *NbNPR1*
NbNPR1-qRT-R	GCTTCATACGCAAATCATCG	
NbPTI-qRT-F	AGGCGTAAGACGGAGACCAT	qPCR primer for *NbPTI*
NbPTI-qRT-R	CCTTAGCACCACGCATTCTA	
NbEDS1-qRT-F	TTGGGCAGAAGTGGAGGAAC	qPCR primer for *NbEDS1*
NbEDS1-qRT-R	AAACATCATCGCCCAGAAGGC	
NbEF1α-qRT-F	AGACCACCAAGTACTACTGCAC	qPCR primer for *NbEF1α*; used as a reference gene for qRT-PCR
NbEF1α-qRT-R	CCACCAATCTTGTACACATCC	
Xa10-F	CGGGATCCATGCAGCTGATGCTCACATTC	Amplifies *Xa10* for cloning into pRTVcHA vector; contains *Bam*HI/*Not*I sites
Xa10-R	TTGCGGCCGCGACGGGGGAAATCTCCTC	
29135g-qRT-F	GGACAACAAGTTCACTAGCCTATTTG	qPCR primer for *29135g*
29135g-qRT-R	AACTTCTCCAAAATAGTAACTCTGATCAAT	
36259g-qRT-F	GGACAACAAGTTCACTAGCCTATCTC	qPCR primer for *36259g*
36259g-qRT-R	CAACTTCTCCAATATAGTAACTCTGATCAAC	
20731g-qRT-F	ATCCAACATCAGATGGTGCAGAG	qPCR primer for *20731g*

20731g-qRT-R	CCAGCTCTTTTGGCAAGTGTTC	
3692g-qRT-F<br>3692g-qRT-R	CAACAAGGACAATATTGCAGTTTCAT<br>TGATCCGTCTCATTAATAGGGCAC	qPCR primer for *3692g*
45656g-qRT-F<br>45656g-qRT-R	GAGGAGTAAGAAAGAGGCCATGG<br>CGGCGACGTCATAAGCCC	qPCR primer for *45656g*
20731g-F<br>20731g-R	CGGGATCCATGGTAACCCTAAAAAGATATGTACATT<br>TTGCGGCCGCGAGTTTGAGAGAAAGATCGATCTTT	Amplifies *Nb20731g* for cloning into pRTVcHA vector; contains *Bam*HI/*Not*I sites
Os13600-F<br>Os13600-R	CGGGATCCATGAAGAGCAGCAGCCAAGAAG<br>TTGCGGCCGCGAGTCTAAGTGTGAGGTCGGGCT	Amplifies *LOC_Os03g13600* for cloning into pRTVcHA vector; contains *Bam*HI/*Not*I sites
Os41110-F<br>Os41110-R	CGGGATCCATGCGAGTCGATAGGGAGAT<br>TTGCGGCCGCCAGCCTGAGGCTCAAGTCTATC	Amplifies *LOC_Os03g41110* for cloning into pRTVcHA vector; contains *Bam*HI/*Not*I sites
Os26210-F<br>Os26210-R	CGGGATCCATGCTCACCACTTTCTCGG<br>TTGCGGCCGCTAGCTTCAGGGACAGGTCG	Amplifies *LOC_Os09g26210* for cloning into pRTVcHA vector; contains *Bam*HI/*Not*I sites
Os44190-F<br>Os44190-R	CGGGATCCATGGAGAAGGAGATCATCAGC<br>TTGCGGCCGCCAACTTGAGAGAAAGGTCGATGT	Amplifies *LOC_Os08g44190* for cloning into pRTVcHA vector; contains *Bam*HI/*Not*I sites
8774g-qRT-F<br>8774g-qRT-R	CTTCCCAAACTTTAAATGCCTTC<br>TTAGTGAAAGATTTAGAAGTGGTGGA	qPCR primer for *8774g*
44252g-qRT-F<br>44252g-qRT-R	TAAGAGCAAGTGTGATTTTCCGG<br>GGCAGGAGCACCAAAATATGTC	qPCR primer for *44252g*
4975g-qRT-F<br>4975g-qRT-R	AATTTCCATGTTCAAGTGCATCTAC<br>GACCTACAAATACTGGAAGACACCC	qPCR primer for *4975g*
36496g-qRT-F<br>36496g-qRT-R	CACCGAGAATGGTAGATCAAAGG<br>CAGCCTGAAAAAATCAACCCC	qPCR primer for *36496g*
p29135g-F<br>p29135g-R	GGAATTCCTAGTCTTAGCAAGTTCTTTCTTTTTG<br>CGGGATCCGAAATAAGTATAATTAAGTCTGTCGAGGAG	Amplifies ~1 kb promoter region of *29135g* for cloning in pCAMBIA1381; contains *Eco*RI/*BamH*I sites
p36259g-F<br>p36259g-R	GGAATTCGTGCTTACTTGTGTATTTTTCCCC<br>CGGGATCCGAAATAAGTATAATTAAGTTTGTCGATG	Amplifies ~1 kb promoter region of *36259g* for cloning into pCAMBIA1381; contains *Eco*RI/*Bam*HI sites
p44252g-F<br>p44252g-R	GGAATTCACTACATAGCTGGGGAATGGG<br>CGGGATCCATGACTATATATTCTACAAAGCAAAGATGC	Amplifies ~1 kb promoter region of *44252g* for cloning into pCAMBIA1381; contains *Eco*RI/*Bam*HI sites

p45656g-F<br>p45656g-R	GGAATTCGAAGGCAGCAATACGAATATTAGAAG<br>CGGGATCCTTTTGTATAGCTGATATGGTGTATTTTGTG	Amplifies ~1 kb promoter region of *45656g* for cloning into pCAMBIA1381; contains *Eco*RI/*Bam*HI sites
29135g-F<br>29135g-R	AACTGCAGATGGATCGCGCACTAATACTC<br>CGAGCTCTTATTCTTTCCATGGAAGTGCA	Amplifies *29135g* for cloning into pHB; contains *Pst*I/*Sac*I sites
36259g-F<br>36259g-R	CGAGCTCATGGCTCGCGCACTAGC<br>CGAGCTCTCAACCTTTTGCTGATGAAGAG	Amplifies *36259g* for cloning into pHB; contains *Sac*I sites
20731g-F<br>20731g-R	AACTGCAGATGGTAACCCTAAAAAGATATGTACATTTAC<br>CGAGCTCTTAGAGTTTGAGAGAAAGATCGATCTTT	Amplifies *20731g* for cloning into pHB; contains *Pst*I/*Sac*I sites
44252g-F<br>44252g-R	AACTGCAGATGGAGCTTAGTGTCTCAAAACTAG<br>CGAGCTCTCACTTGTGACATGCCATGTTC	Amplifies *44252g* for cloning into pHB; contains *Pst*I/*Sac*I sites
45656g-F<br>45656g-R	AACTGCAGATGGCTATGAAAGAAAAGCTAAATG<br>AACTGCAGTCAAGCTTCCATTGGTGGG	Amplifies *45656g* for cloning into pHB; contains *Pst*I sites
3692g-F<br>3692g-R	AACTGCAGATGGAATTTGGAGCTACATTTGAT<br>CGAGCTCTTAAGTATTCTTACCTGGTTGGAGC	Amplifies *3692g* for cloning into pHB; contains *Pst*I/*Sac*I sites
29135g-F<br>29135g-R	ttaagaggagtccacCATGGATCGCGCACTAATACTC<br>gtcaccaattcacacgtgatggtgatggtgatgTTCTTTCCATGGAAGTGCA	Amplifies *29135g* for cloning into pCAMBIA1381 by replacing *gusA*
36259g-F<br>36259g-R	ttaagaggagtccacCATGGCTCGCGCACTAGC<br>gtcaccaattcacacgtgatggtgatggtgatgTTCTTTCCATGGAAGTGCAA	Amplifies *36259g* for cloning into pCAMBIA1381 by replacing *gusA*
20731g-F<br>20731g-R	CATGCCATGGTAACCCTAAAAAGATATGTACATTTAC<br>ACCCACGTGatggtgatggtgatgGAGTTTGAGAGAAAGATCGATCTTT	Amplifies *20731g* for cloning into pCAMBIA1381 by replacing *gusA*
44252g-F<br>44252g-R	CATGCCATGGAGCTTAGTGTCTCAAAACTAG<br>ACCCACGTGatggtgatggtgatgCTTGTGACATGCCATGTTC	Amplifies *44252g* for cloning into pCAMBIA1381 by replacing *gusA*
45656g-F<br>45656g-R	ttaagaggagtccacCATGGCTATGAAAGAAAAGCTAAATG<br>gtcaccaattcacacgtgatggtgatggtgatgAGCTTCCATTGGTGGG	Amplifies *45656g* for cloning into pCAMBIA1381 by replacing *gusA*
3692g-F<br>3692g-R	ttaagaggagtccacCATGGAATTTGGAGCTACATTTGAT<br>gtcaccaattcacacgtgatggtgatggtgatgAGTATTCTTACCTGGTTGGAGC	Amplifies *3692g* for cloning into pCAMBIA1381 by replacing *gusA*

20731g(EBE)-F	CTAATATATATAAGCACATCTCTTCCTCC	EBE fragment for EMSA
20731g(EBE)-R	GGAGGAAGAGATGTGCTTATATATATTAG	
44252g(EBE)-F	GTATATATATACACACATCCCCCATTTAAC	EBE fragment for EMSA
44252g(EBE)-R	GTTAAATGGGGGATGTGTGTATATATATAC	
45656g(EBE)-F	CTCCATTATATAAGCACATCTACACATC	EBE fragment for EMSA
45656g(EBE)-R	GATGTGTAGATGTGCTTATATAATGGAG	
NbZnFP1-F	AACTGCAGATGGTAACCCTAAAAAGATATGTACATTTAC	Amplifies *NbZnFP1* for cloning into pHB vector; contains *Pst*I/*Sac*I sites
NbZnFP1-R	CGAGCTCTTAGAGTTTGAGAGAAAGATCGATCTTT	
NbZnFP1(VIGS)-F	GCTCTAGAACCCCATAGGTGGTCAAGAC	Used for on-spot silencing; amplifies 285 bp of *NbZnFP1* for VIGS construct; contains *Xba*I/*Bam*HI restriction sites
NbZnFP1(VIGS)-R	CGGGATCCTGCACGAGAATACTCTTGGC	
NbZnFP1-YFP-F	GGGGTACCATGGTAACCCTAAAAAGATATGTACATTTAC	Amplifies *NbZnFP1* for cloning into pYFP vector; contains *Kpn*I/*Sma*I sites
NbZnFP1-YFP-R	TCCCCCGGGGAGTTTGAGAGAAAGATCGATCTTTTG	
Solyc03g117070(qRT)-F	ATCTACGCCTCGATCACGG	qPCR primer for *Solyc03g117070*
Solyc03g117070(qRT)-R	GCGAACTGTAAAATTTCCTCTGG	
Solyc07g006880(qRT)-F	ATGAGTTATGAACCAAACACGGC	qPCR primer for *Solyc07g006880*
Solyc07g006880(qRT)-R	GCAAGAAAAAACACGTGGCTC	
Solyc00g014800(qRT)-F	TGTTTACGATTTCTGGAACCAGTC	qPCR primer for *Solyc00g014800*
Solyc00g014800(qRT)-R	AAACTTGCGAGAACAGTAGAGGC	
Solyc10g078990(qRT)-F	GAGTCCTCCTCTCAACTTCCTATTAC	qPCR primer for *Solyc10g078990*
Solyc10g078990(qRT)-R	TTTCCTCATGATTTCAACATTCTTC	
SolyEF1α(qRT)-F	GATTGACAGACGTTCTGGTAAGGA	qPCR primer for *SolyEF1α*
SolyEF1α(qRT)-R	ACCGGCATCACCATTCTTCA	
Os03g13600(qRT)-F	TGAAGAGCAGCAGCCAAGAAG	qPCR primer for *Os03g13600*
Os03g13600(qRT)-R	GTGAGCGACAGGTCGAGGC	
Os12g39220(qRT)-F	CGAGTCGTCGGTGAACAAAGAG	qPCR primer for *Os12g39220*

Os12g39220(qRT)-R	GTTTGCTGCTTCTGTTTCTTCTGC	
Os08g17640(qRT)-F	CTTCCAGCTCGCAGACCACC	qPCR primer for *Os08g17640*
Os08g17640(qRT)-R	CTCCTCCTCCTTCTTGGCCAC	
Os02g08510(qRT)-F	AGGAGGAGGCGACGTGACG	qPCR primer for *Os02g08510*
Os02g08510(qRT)-R	GACACGACGAGCAGACGAGG	
Os08g44190(qRT)-F	ATGGAGAAGGAGATCATCAGCG	qPCR primer for *Os08g44190*
Os08g44190(qRT)-R	AGCCGCAGGTCGAGGTTG	
Os07g40300(qRT)-F	AAGTGGTGCAACACAAGGCAG	qPCR primer for *Os07g40300*
Os07g40300(qRT)-R	GGCTTCGGATTCGACTCG	
Os09g38610(qRT)-F	GGCGGCGTCAACCTCAAC	qPCR primer for *Os09g38610*
Os09g38610(qRT)-R	GGCAGTAGTTGCAGGAGAAGGC	
Os03g41110(qRT)-F	CGATAGGGAGATGGAGAGAGGG	qPCR primer for *Os03g41110*
Os03g41110(qRT)-R	AAGGAGAACCCTGCTGCTGC	
Os09g26210(qRT)-F	ATGCTCACCACTTTCTCGGC	qPCR primer for *Os09g26210*
Os09g26210(qRT)-R	GAGGTCCAGCTGCTCATCCC	
OsActin(qRT)-F	GTTCCTGCTGTTTGTTCTGTTG	qPCR primer for *OsActin*
OsActin(qRT)-R	ATCTCACGCATTACCCTACCTT	

Supplemental Dataset 1. Script for promoter extraction from *N. benthamiana* genome

Supplemental Dataset 2: List of genes up-regulated by AvrXa10 in *Nb* and putative target genes containing EBEs recognized by AvrXa10.

Supplemental References

Cai, L., Cao, Y., Xu, Z., Ma, W., Zakria, M., Zou, L., Cheng, Z., and Chen, G. (2017). A transcription activator-like effector Tal7 of *Xanthomonas oryzae* pv. *oryzicola* activates rice gene *Os09g29100* to suppress rice immunity. Sci. Rep **7**:5089.

Haq, F., Xie, S., Huang, K., Shah, S.M.A., Ma, W., Cai, L., Xu, X., Xu, Z., Wang, S., and Zou, L. (2020). Identification of a virulence *tal* gene in the cotton pathogen, *Xanthomonas citri* pv. *malvacearum* strain Xss-V_2–18. BMC Microbiol. **20**:1-13.

He, F., Zhang, F., Sun, W., Ning, Y., and Wang, G. (2018). A versatile vector toolkit for functional analysis of rice genes. Rice **11**:1-10.

Hopkins, C.M., White, F., Choi, S., Guo, A., and Leach, J. (1992). Identification of a family of avirulence genes from *Xanthomonas oryzae* pv. *oryzae*. Mol. Plant-Microbe Interact. **5**:451-459.

Ji, Z., Ji, C., Liu, B., Zou, L., Chen, G., and Yang, B. (2016). Interfering TAL effectors of *Xanthomonas oryzae* neutralize R-gene-mediated plant disease resistance. Nat. Commun **7**:1-9.

Ma, W., Zou, L., Ji, Z., Xu, X., Xu, Z., Yang, Y., Alfano, J.R., and Chen, G. (2018). *Xanthomonas oryzae* pv. *oryzae* TALE proteins recruit OsTFIIAγ1 to compensate for the absence of OsTFIIAγ5 in bacterial blight in rice. Mol. Plant Pathol. **19**:2248-2262.

Ma, W., Xu, X., Cai, L., Cao, Y., Haq, F., Alfano, J.R., Zhu, B., Zou, L., and Chen, G. (2020). A *Xanthomonas oryzae* type III effector XopL causes cell death through mediating ferredoxin degradation in *Nicotiana benthamiana*. Phytopathology Res. **2**:1-12.

Mao, J., Zhang, Y., Sang, Y., Li, Q., and Yang, H. (2005). A role for *Arabidopsis* cryptochromes and COP1 in the regulation of stomatal opening. Proc. Natl. Acad. Sci. USA **102**:12270-12275.

Xu, X., Xu, Z., Ma, W., Haq, F., Li, Y., Shah, S.M.A., Zhu, B., Zhu, C., Zou, L., and Chen, G. (2021). TALE-triggered and iTALE-suppressed *Xa1* resistance to bacterial blight is independent of *OsTFIIAγ1* or *OsTFIIAγ5* in rice. J. Exp. Bot.

Zhao, Y., Liu, W., Xu, Y.-P., Cao, J.-Y., Braam, J., and Cai, X.-Z. (2013). Genome-wide identification and functional analyses of calmodulin genes in *Solanaceous* species. BMC Plant Biol. **13**:1-15.

Zhu, W., Yang, B., Chittoor, J.M., Johnson, L.B., and White, F.F. (1998). AvrXa10 contains an acidic transcriptional activation domain in the functionally conserved C terminus. Mol. Plant-Microbe Interact. **11**:824-832.
